# Tracing the Origin and Evolution of the Fungal Mycophenolic Acid Biosynthesis Pathway

**DOI:** 10.1093/gbe/evaf039

**Published:** 2025-03-07

**Authors:** Baptiste Bidon, Hajar Yaakoub, Arnaud Lanoue, Antoine Géry, Virginie Séguin, Florent Magot, Claire Hoffmann, Vincent Courdavault, Jean-Philippe Bouchara, Jean-Pierre Gangneux, Jens C Frisvad, Antonis Rokas, Gustavo H Goldman, Gilles Nevez, Solène Le Gal, Domenico Davolos, David Garon, Nicolas Papon

**Affiliations:** Univ Angers, Univ Brest, IRF, SFR ICAT, F-49000 Angers, France; Centre for Genomics and Precision Medicine, National Taiwan University, Taipei, Taiwan (R.O.C.); Univ Angers, Univ Brest, IRF, SFR ICAT, F-49000 Angers, France; Nantes Université, INRAE UMR-1280 PhAN, F-44000 Nantes, France; Université de Tours, BBV EA2106, Tours, France; ABTE EA 4651-ToxEMAC, Normandie Université, UNICAEN, UNIROUEN, Caen, France; ABTE EA 4651-ToxEMAC, Normandie Université, UNICAEN, UNIROUEN, Caen, France; Université de Tours, BBV EA2106, Tours, France; Univ Angers, Univ Brest, IRF, SFR ICAT, F-49000 Brest, France; Parasitology-Mycology Unit, Brest University Hospital, Brest, France; Université de Tours, BBV EA2106, Tours, France; Univ Angers, Univ Brest, IRF, SFR ICAT, F-49000 Angers, France; Univ Rennes, CHU Rennes, Inserm, EHESP, Irset (Institut de recherche en santé, environnement et travail)-UMR_S 1085, Rennes, France; Parasitology-Mycology Unit, Rennes University Hospital, European Excellence Center in Medical Mycology (ECMM EC), Centre National de Référence pour les mycoses et antifongiques-laboratoire associé Aspergilloses chroniques (CNRMA-LA AspC), Rennes, France; Department of Biotechnology and Biomedicine, Technical University of Denmark, Kongens Lyngby, Denmark; Department of Biological Sciences, Vanderbilt University, Nashville, TN, USA; Vanderbilt Evolutionary Studies Initiative, Vanderbilt University, Nashville, TN, USA; Faculdade de Ciências Farmacêuticas de Ribeirão Preto, Universidade de São Paulo, Ribeirão Preto, Brazil; National Institute of Science and Technology in Human Pathogenic Fungi, Universidade de São Paulo, Ribeirão Preto, Brazil; Univ Angers, Univ Brest, IRF, SFR ICAT, F-49000 Brest, France; Parasitology-Mycology Unit, Brest University Hospital, Brest, France; Univ Angers, Univ Brest, IRF, SFR ICAT, F-49000 Brest, France; Parasitology-Mycology Unit, Brest University Hospital, Brest, France; Department of Technological Innovations and Safety of Plants, Products and Anthropic Settlements (DIT), INAIL Research Area, Rome, Italy; ABTE EA 4651-ToxEMAC, Normandie Université, UNICAEN, UNIROUEN, Caen, France; Univ Angers, Univ Brest, IRF, SFR ICAT, F-49000 Angers, France

**Keywords:** secondary metabolism, biosynthetic pathway, antifungal, immunosuppressant, biosynthesis

## Abstract

Like bacteria and plants, fungi produce a remarkable diversity of small molecules with potent activities for human health known as natural products or secondary metabolites. One such example is mycophenolic acid, a powerful immunosuppressant drug that is administered daily to millions of transplant recipients worldwide. Production of mycophenolic acid is restricted to a very limited number of filamentous fungi, and little is known about its biosynthetic modalities. It is therefore a particular challenge to improve our knowledge of the biosynthesis of this valuable natural compound, as this would contribute to a better understanding of the specialized metabolism of fungi and could also lead to the identification of new fungal producers for the supply of immunosuppressants. Here, we were interested in deciphering the origin and evolution of the fungal mycophenolic acid biosynthetic pathway. Large-scale analyses of fungal genomic resources led us to identify several new species that harbor a gene cluster for mycophenolic acid biosynthesis. Phylogenomic analysis suggests that the mycophenolic acid biosynthetic gene cluster originated early in a common ancestor of the fungal family *Aspergillaceae* but was repeatedly lost and it is now present in a narrow but diverse set of filamentous fungi. Moreover, a comparison of the inosine 5′-monophosphate dehydrogenase protein sequences that are the target of the mycophenolic acid drug as well as analysis of mycophenolic acid production and susceptibility suggest that all mycophenolic acid fungal producers are resistant to this toxic compound, but that this resistance is likely to be based on different molecular mechanisms. Our study provides new insight into the evolution of the biosynthesis of the important secondary metabolite mycophenolic acid in fungi.

**Significance**

Although previously described in a few *Penicillium* species, the molecular basis of mycophenolic acid production has never been studied in depth across the entire fungal kingdom. By sequencing the genome of 4 new fungal species and performing a phylogenomic analysis of 479 mold genomes, we show that mycophenolic acid biosynthesis was maintained in a restricted number of molds belonging to the genera *Penicillium, Aspergillus*, and *Paecilomyces*. This study thus provides unprecedented insight into the origin, distribution, and evolution of the biosynthesis of a major secondary metabolite in fungi.

## Introduction

Secondary metabolites were once perceived as trivial by-products of the primary metabolism of living organisms (Hartmann 2007). Intensified research in analytical chemistry has progressively led to reconsidering these secondary metabolites as diverse and multifunctional compounds, often produced by complex biosynthetic pathways, and used as competitive weapons during the everlasting chemical warfare occurring in nature (Demain and Fang 2000). Among competing organisms, plants, fungi, and bacteria constitute the major producers of the expanding inventory of known secondary metabolites, of which many have found their way into the pharmaceutical and agrochemical industries (Anwar et al. 2019; Devi et al. 2020; Ramírez-Rendon et al. 2022). In many of these organisms, genes encoding enzymes that catalyze the successive steps of secondary metabolite biosynthesis are often arranged in biosynthetic gene clusters (BGCs) (Cimermancic et al. 2014; Nützmann et al. 2016; Rokas et al. 2018, 2020). While the organization, regulation, and evolution of a broad range of bacterial secondary metabolic pathways are now fairly well established, those of plant and fungal origin are still fragmentary (Keller 2019; Méteignier et al. 2023). Investigating the divergence of fungal BGCs may help to identify the ecological roles of such metabolites and understand the evolution of fungal chemodiversity. Such research is also advantageous for a deeper understanding of fungal biology, particularly that related to virulence and resistance to chemical agents since more active roles in fungal physiology are being attributed to secondary metabolites (Macheleidt et al. 2016; Keller 2019; Avalos and Limón 2022).

Fungal secondary metabolites are numerous and classified into various molecular families (Robey et al. 2021), belonging to three major biosynthetic classes: polyketides (PK), nonribosomal peptides (NRP), and terpenes (Keller et al. 2005). The importance of these organic molecules in biotechnology and medicine has taken hold since the advent of penicillin G, the NRP molecule that stood for a long time as the mainstay of the antibiotic arsenal (Devi et al. 2020; Yuan et al. 2020; Galindo-Solís and Fernandez 2022). Other examples of fungi-derived secondary metabolites with great value in the medicinal arena include the NRP antifungal echinocandins (Hüttel 2017), the cholesterol-lowering PK agent lovastatin (Goswami et al. 2013), and the terpene-PK hybrid mycophenolic acid (MPA) (Bentley 2000). MPA has multiple applications in human therapy, primarily used as an immunosuppressant, and represents a global market size of US 1.5 billion annually (Abd Rahman et al. 2013). Structurally, MPA is a meroterpenoid consisting of a terpenoid side chain coupled with a nonterpenoid moiety of phthalide nature. The immunosuppression mechanism of action of MPA is the noncompetitive inhibition of inosine 5′-monophosphate dehydrogenase (IMPDH), which is the rate-limiting enzyme in de novo guanine nucleotide biosynthesis indispensable for lymphocyte proliferation (Hedstrom 2009).

To date, MPA production by fungi has been demonstrated for a restricted number of species belonging to the genera *Penicillium* and *Aspergillus* (Chen et al. 2017; Mouhamadou et al. 2017; Sklenář et al. 2017; Houbraken et al. 2020). Most of the well-known MPA producers were isolated from agricultural soil, silage, fruit, cereals, and dairy products (Puel et al. 2005; Vinokurova et al. 2005; Séguin et al. 2014; Mouhamadou et al. 2017; Mahmoudian et al. 2021; Ammar et al. 2023). This likely suggests that MPA production confers fitness advantages for survival in particular ecological niches, which is not unusual for fungal secondary metabolites as similar roles have been assigned to many of them (Keller 2019). However, it is premature to make any deduction since data on MPA production in fungi is almost based on the activity-guided approach. This is problematic since it lacks reliability when considering the context of strain- and culture-dependent production of MPA (Fontaine et al. 2015). So, any unbiased survey for MPA production and exploration of its role in the publicly available genome sequences is of great importance.

**Fig. 1.**
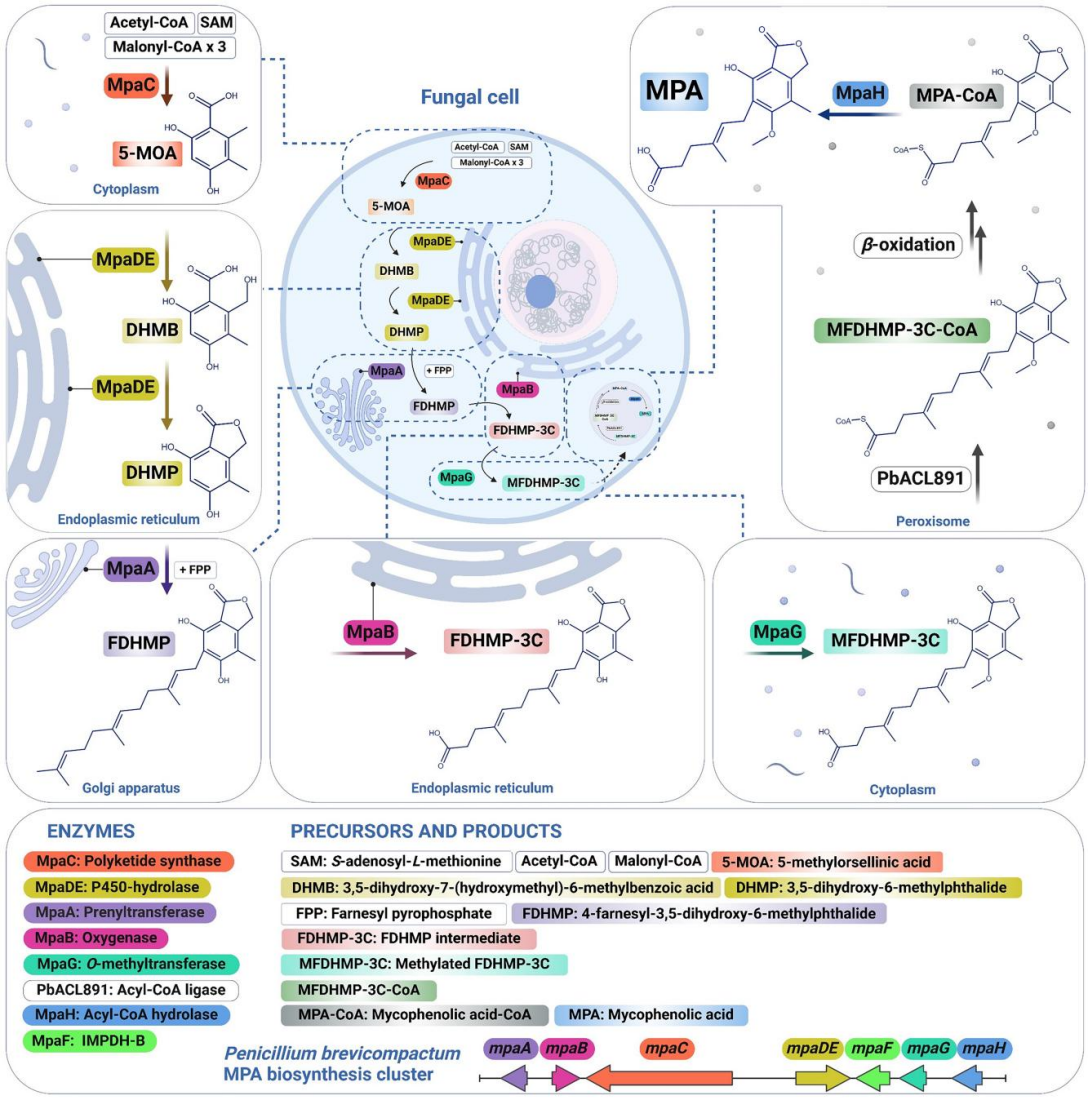
MPA biosynthetic pathway in *Penicillium brevicompactum*. The first step resides in the assembly of 5-methylorsellinic acid (5-MOA) from one acetyl-CoA, three malonyl-CoA, and one *S*-adenosyl-*L*-methionine molecule, which is mediated by the cytosolic PK synthase MpaC. This step is cardinal for the process so that the encoding gene is considered a molecular tool to screen for MPA production (Gillot et al. 2017; Mouhamadou et al. 2017). In the next step of MPA production, 5-MOA is converted to the phthalide intermediate 3,5-dihydroxy-6-methylphthalide (DHMP) through hydroxylation and lactonization mediated by the endoplasmic reticulum-bound P450-hydrolase fusion enzyme MpaDE (Hansen et al. 2012a). Then, the Golgi apparatus-associated farnesyl-transferase MpaA catalyzes the farnesylation of DHMP into 4-farnesyl-3,5-dihydroxy-6-methylphthalide (FDHMP). The latter is next subjected to oxidative cleavage by the ER-bound oxygenase MpaB, which yields a mycophenolic aldehyde (FDHMP-3C). Subsequently, a methylation step mediated by the cytosolic *O*-methyltransferase MpaG gives MFDHMP-3C (Zhang et al. 2015), which in turn is transformed to MFDHMP-3C-CoA by the peroxisomal acyl-CoA ligase PbACL891 (encoded by an MPA cluster-independent gene) (Zhang et al. 2019). Successive β-oxidation steps of MFDHMP-3C-CoA within the peroxisome result in the formation of MPA-CoA, which is finally hydrolyzed by the peroxisomal acyl-CoA hydrolase MpaH to give the final product MPA (You et al. 2021). The scheme was adapted from Zhang et al. (2019) and designed using Biorender.

Our knowledge concerning the molecular basis of MPA production stems from a couple of *Penicillium* species, namely *P. brevicompactum* (Hansen et al. 2011b; Regueira et al. 2011; Zhang et al. 2015, 2019) and *P. roqueforti* (Del-Cid et al. 2016; Gillot et al. 2017). In both species, MPA biosynthetic pathway enzymes are encoded by a 7-gene cluster (*mpaA*, *mpaB*, *mpaC*, *mpaDE*, *mpaF*, *mpaG*, and *mpaH*) (Fig. 1). There are currently no studies that have addressed the modalities of MPA production across the genus *Aspergillus.*

**Fig. 2.**
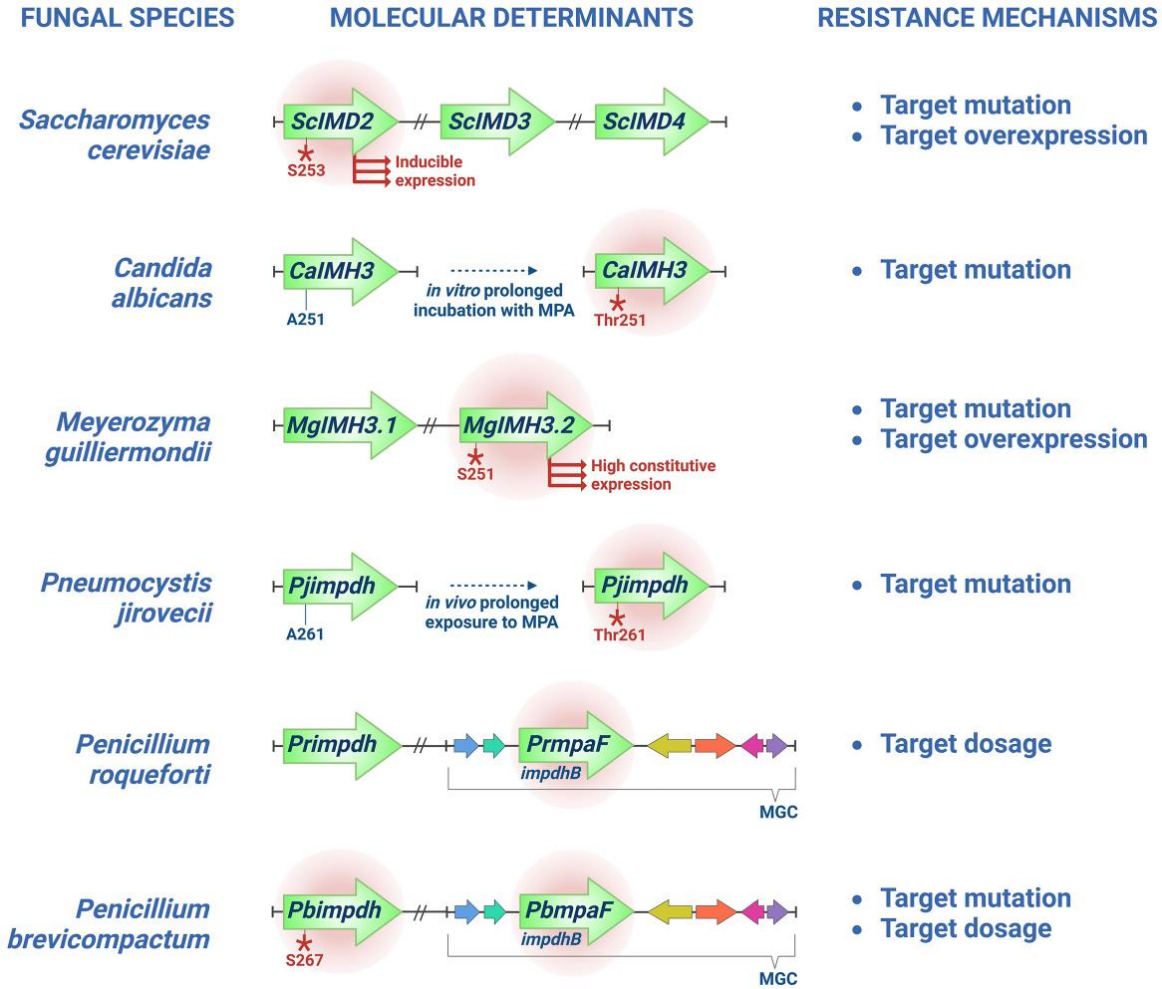
MPA resistance mechanisms in fungi. Among the three genes encoding functional IMPDHs (*imd2*, *imd3*, and *imd4*) in *Saccharomyces cerevisiae*, only *imd2* is responsible for MPA resistance. The gene encodes an MPA-resistant IMPDH due to an Ala253Ser substitution, and it is overexpressed in an MPA-dependent manner (Shaw and Reines 2000; Escobar-Henriques and Daignan-Fornier 2001; Shaw et al. 2001; Hyle et al. 2003; McPhillips et al. 2004; Jenks and Reines 2005). The expression of multiple copies of *Candida albicans* IMPDH-encoding gene (referred to as *IMH3*) or prolonged MPA exposure-induced Ala251Thr substitution (equivalent to *S. cerevisiae* 253 position) results in decreased susceptibility to MPA (Köhler et al. 1997, 2005). In *Meyerozyma guilliermondii*, MPA resistance is due to the presence of an additional IMPDH-encoding gene (*MgIMH3.2*) of which expression is constitutively high and encodes an Imh3.2 protein with Ala251Ser substitution (Defosse et al. 2016). MPA resistance in *Pneumocystis jirovecii* is also due to an Ala261Ser substitution (equivalent to *S. cerevisiae* 253 position) in the predicted IMPDH protein (encoded by *Pjimpdh*), which is likely the result of long-term exposure to MPA of transplant patients colonized by this fungus (Hoffmann et al. 2021). In MPA-producing species like *Penicillium roqueforti* and *P. brevicompactum*, self-resistance is provided by *the mpaF* gene (*PrmpaF* and *PbmpaF*, respectively) located in the MPA gene cluster (MGC). The gene encodes an additional but atypical variant of IMPDH enzyme (type B), in which determinants of resistance are still unknown. In addition to the *PbmpaF* gene, the Ala267Ser substitution (equivalent to *S. cerevisiae* 253 position) in the typical IMPDH protein (encoded by *Pbimpdh*) also lags behind resistance to MPA in *P. brevicompactum* (Hansen et al. 2011a, 2012b; Freedman et al. 2020). *S. cerevisiae*, *C. albicans*, and *M. guilliermondii* are part of the *Saccharomycotina* clade, *P. jirovecii* is part of the *Taphrinomycotina*, and *P. roqueforti and P. brevicompactum* are part of the *Pezizomycotina.*

Importantly, *mpaF* encodes an additional copy of the MPA target protein (IMPDH), which confers self-protection to the producing organism against the drug (Hansen et al. 2012b; Del-Cid et al. 2016; Fig. 2). Resistance to MPA has also been reported in nonproducing species, for which it has been associated with exposure to the molecules either in nature or human host (Defosse et al. 2016; Freedman et al. 2020; Hoffmann et al. 2021). In light of the high vulnerability of immunosuppressed populations to life-threatening mycoses (Denning 2024), depicting mechanisms of resistance to MPA in environmental fungi may be advantageous in carefully preventing the emergence of MPA exposure-induced resistant strains (Hoffmann et al. 2021; Papon et al. 2021). Resistance to MPA in fungal species can result from at least three distinct molecular mechanisms that may or may not occur at the same time: i.e. (i) transcriptional upregulation of target gene *IMPDH*, (ii) IMPDH gene dosage, (iii) and IMPDH substitutions that confer resistance (Fig. 2). Pioneering studies on *Saccharomyces cerevisiae* showed that among the three genes encoding functional IMPDHs (*imd2*, *imd3*, and *imd4*), *imd2* is structurally and transcriptionally distinct. The *imd2* gene indeed encodes an MPA-resistant IMPDH due to an Ala253Ser substitution and features a GRE (guanine-responsive element) in the promoter believed to allow MPA-induced overexpression (Shaw and Reines 2000; Escobar-Henriques and Daignan-Fornier 2001; Shaw et al. 2001; Hyle et al. 2003; McPhillips et al. 2004; Jenks and Reines 2005; Fig. 2). Similar findings in *Candida albicans* reported that resistance stems from the expression of multiple copies of the IMPDH-encoding gene *IMH3* or from the acquisition of an Ala251Thr substitution in cells exposed to MPA (Köhler et al. 1997, 2005; Fig. 2). In *Meyerozyma guilliermondii*, MPA resistance relies on the constitutive expression of an additional IMPDH-encoding gene (*MgIMH3.2*) with the Ala251Ser substitution in the predicted Imh3.2 protein (Defosse et al. 2016; Fig. 2). A recent paper published by our group proved that long-term exposure of transplant patients to MPA drives the selection of *Pneumocystis jirovecii* strains that harbor an Ala261Thr substitution in the predicted PjIMPDH (Hoffmann et al. 2021; Fig. 2). Consistently, swapping the corresponding residue of IMPDH in *A. nidulans* and *P. brevicompactum* (i.e. Ala222Ser and Ser273Ala substitutions, respectively) renders AnIMPDH MPA-resistant while PbIMPDH becomes more sensitive to the drug (Freedman et al. 2020). On the other hand, and as noted above, the self-resistance to MPA in the MPA-producing molds stems from the *mpaF* gene that resides within the BGC. The *mpaF* gene encodes a second isoform of the MPA target protein (referred to as IMPDH-B) that is cladistically different from the typical copy shared by all fungal species (i.e. IMPDH-A) (Hansen et al. 2011a, 2012b). How *mpaF* provides resistance to MPA is not completely understood—at least from a structural point of view. The fact that several species of *Penicillium* subgen. *Penicillium* identified as nonproducers harbor both IMPDH isoforms which could be the consequence of a gene duplication event or the result of gene retention after the cluster degeneration. However, how the two events are linked or influenced each other is still uncertain, highlighting the importance of reconstructing the evolution of MPA production across fungi.

As more fungal genomes become available, their analysis may help us to better understand the co-evolution of MPA biosynthesis and cognate resistance across fungi. In this study, we aimed to (i) map the BGC for MPA production in four newly sequenced genomes of *Aspergillus* species and in the publicly available genomes of filamentous fungi and (ii) analyze the production of MPA and the associated mechanisms of resistance in a set of mold species.

## Results

### Whole-Genome Sequencing of Xerophilic *Aspergillus* Species

We selected four strains (E2, E25, E30, and E42) belonging to different series of xerophilic *Aspergillus* and previously analyzed for MPA production by Mouhamadou et al. (2017) for whole-genome sequencing. The sampling attempted to include strains from as distinct as possible niches and, importantly, Mouhamadou et al. detected MPA production in E42 but not in E2, E25, and E30. We report here the first record of complete-genome sequencing of two species from series *Rubri* including *Aspergillus ruber* strain E2 and *Aspergillus pseudoglaucus* strain E42, one species from series *Chevalierorum* i.e. *A. intermedius* strain E25, but also one species from series *Aspergillus* (*A. proliferans* strain E30). Sequencing was performed using regular Illumina paired-end short-reads technology, except for E42 which also required Nanopore long-reads technology to accurately cover the MPA BGC. Strain origins and genome assembly outputs, including genome sizes, scaffold numbers, and hypothetical coding genes, are compiled in Table 1.

**Table 1:** Genome assembly features of sequenced xerophilic *Aspergilli*

Species	Strain	Size (Mb)	GC (%)	Scaffolds	N50	Genes	Source	Accession ID
*Aspergillus pseudoglaucus*	E42	26.592	49.08	174	599,006	8,473	Air, France	SAMN25554604
*Aspergillus proliferans*	E30	28.932	49.32	927	79,997	9,551	Jam, France	SAMN25554603
*Aspergillus intermedius*	E25	26.817	49.22	565	233,773	8,641	Soil, Israel	SAMN25554602
*Aspergillus ruber*	E2	26.250	48.81	956	84,791	8,451	Leather, China	SAMN25554601

### Identification of BGC Putatively Involved in MPA Biosynthesis in *Aspergillus* Species

**Fig. 3.**
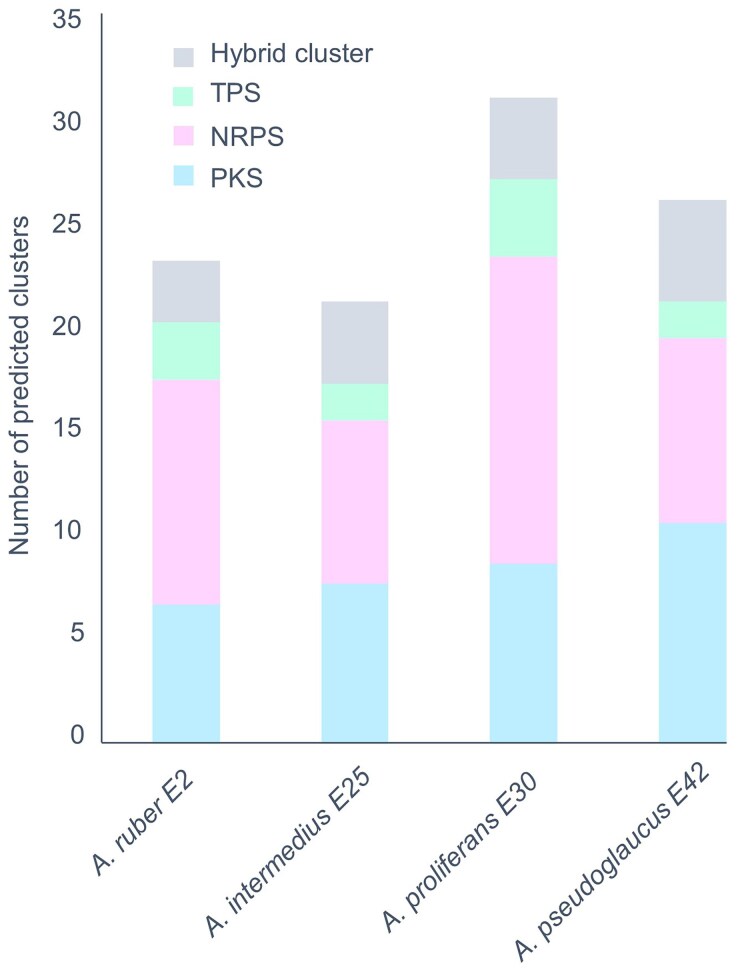
Inference of BGCs in xerophilic *Aspergillus* species. The inference of BGCs was performed using antiSMASH fungi version, on whole de novo assembled genome sequences for *A. ruber* E2, *A. intermedius* E25, *A. proliferans* E30, and *A. pseudoglaucus* (E42). Terpene synthase (TPS), polyketide synthase (PKS), nonribosomal peptide synthetase (NRPS). The number of BGC varies between ∼20 and 30 per genome in the four newly sequenced xerophilic fungi.

Whole-genome sequences of the four newly sequenced *A. ruber* E2, *A. intermedius* E25, *A. proliferans* E30, and *A. pseudoglaucus* E42 were first browsed to analyze for the presence of BGCs. As shown in Fig. 3, the number of BGC varies between ∼20 and 30 per genome, globally aligning with that previously revealed in *Aspergillus* species (Robey et al. 2021). The distribution of BGC biosynthetic mechanisms within the strain genomes shows similar patterns in which nonribosomal peptide synthetases stand out as the most abundant family of secondary metabolites, followed by PKSs, hybrid BGC, and terpene synthases.

**Fig. 4.**
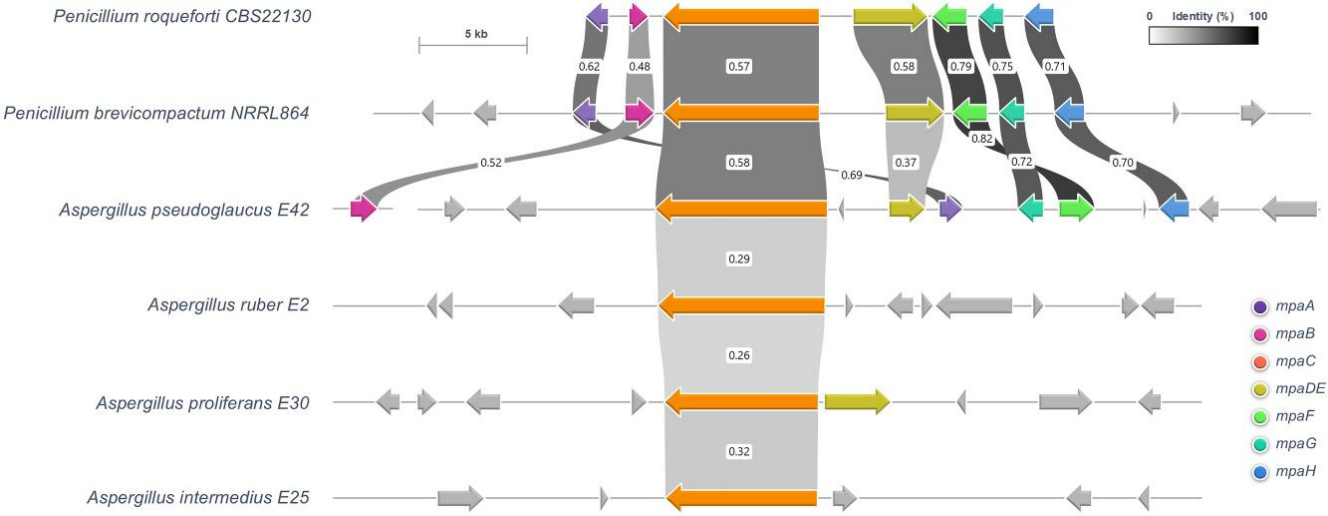
Identification of BGCs putatively involved in MPA biosynthesis in xerophilic *Aspergillus* species. Cluster was identified by blast using *P. brevicompactum* NRRL864 and *P. roqueforti* CBS 22130 BGC sequences as references. Sequences were then extracted with bedtools and synteny was constructed with clinker v0.0.23. *A. pseudoglaucus* E42 possesses a BGC composed of six genes displaying high sequence identity scores with *mpaA*, *mpaC*, *mpaDE*, *mpaF*, *mpaG*, and *mpaH*. The *mpaB* gene is located out of this cluster. A partial BGC including *mpaC* and *mpaDE* genes was found in *A. proliferans* E30. Only a *mpaC* homolog was detected in *A. intermedius* E25 and *A. ruber* E2. Gray arrows surrounding the highlighted BGC correspond to strain-specific genes without any orthology between species. Identity scores are indicated for each pair of closest homologs.

We then examined more specifically the presence of the MPA BGC in the four newly sequenced *Aspergillus* genomes using the MPA BGCs of *P. brevicompactum* and *P. roqueforti* as queries. BLAST analysis shows that only *A. pseudoglaucus* E42 possesses a BGC composed of six genes; these six genes display significant sequence identity scores with *P. brevicompactum* and *P. roqueforti mpaA*, *mpaC*, *mpaDE*, *mpaF*, *mpaG*, and *mpaH.* Surprisingly, the gene organization of the cluster detected in *A. pseudoglaucus* E42 differs from the MPA BGC previously characterized in *P. brevicompactum* and *P. roqueforti* (Fig. 4). In addition, the seventh gene *mpaB* is located out of this BGC. The other three species examined contain only fragments of the MPA BGC; the *A. proliferans* E30 genome contains only the *mpaC* and *mpaDE* genes, while we only detected a *mpaC* homolog in *A. intermedius* E25 and *A. ruber* E2 (Fig. 4).

### Large-Scale Genomic Analysis of MPA BGCs in *Eurotiales*

**Fig. 5.**
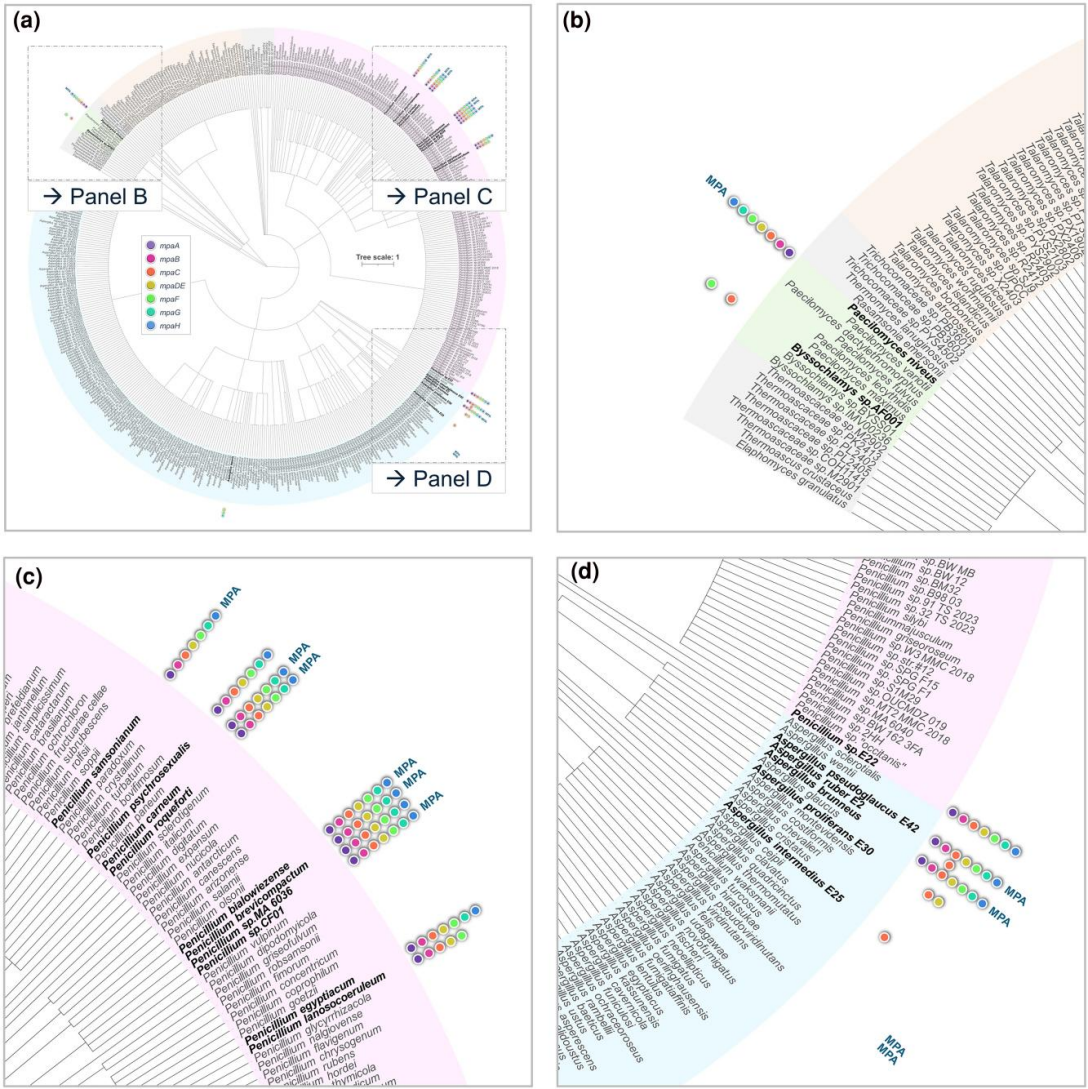
Large-scale genomic analysis of MPA BGCs in *Eurotiales.* Cladogram representing the phylogenetic organization of 479 *Eurotiales* (family, genus, species, sections, and series) according to the data published by Houbraken et al. (2020). Color circles represent identified ORF coding for MPA cluster genes in their reference genomes. The whole cladogram is shown in (a). Panels B, C, and D provide an enlargement of specific frames of the cladogram, and are shown in (b), (c), and (d), respectively. The previously reported or the hereafter production of MPA is indicated next to the BGC color circles. Strain names and genome information are compiled in supplementary table S1, Supplementary Material online and genomic coordinates of complete MPA BGCs are provided in supplementary table S2, Supplementary Material online.

In addition to *P. brevicompactum*, *P. roqueforti*, and some xerophilic *Aspergilli*, several species belonging to the *Eurotiales* order were reported in the past decades to be able to produce MPA (Houbraken et al. 2020). However, no study has ever systematically addressed the presence and distribution of MPA BGCs in this order comprising nearly 500 available genome sequences. We thus mapped the presence of putative MPA BGCs across the *Eurotiales* order by analyzing 479 publicly available genomes to which we added the data obtained in this study concerning *A. ruber* E2, *A. intermedius* E25, *A. proliferans* E30, and *A. pseudoglaucus* E42 (strain names and genome information are compiled in supplementary table S1, Supplementary Material online). As depicted in Fig. 5, and in addition to *P. brevicompactum*, *P. roqueforti*, and *A. pseudoglaucus* E42, we detected the presence of a putative MPA BGC in a total of ten species/strains including *Paecilomyces niveus* (formerly *Byssochlamys nivea*), *P. carneum* (*Roquefortorum* section), *P. swiecickii* 182_6C1 (*Osmophila* section), *Penicillium* sp. MA6036, *Penicillium* sp. CF01 of section *Brevicompacta*, *P. psychrosexualis*, *P. bialowiezense*, *P. egyptiacum*, *Penicillium* sp. E22, and *A. brunneus*.

**Fig. 6.**
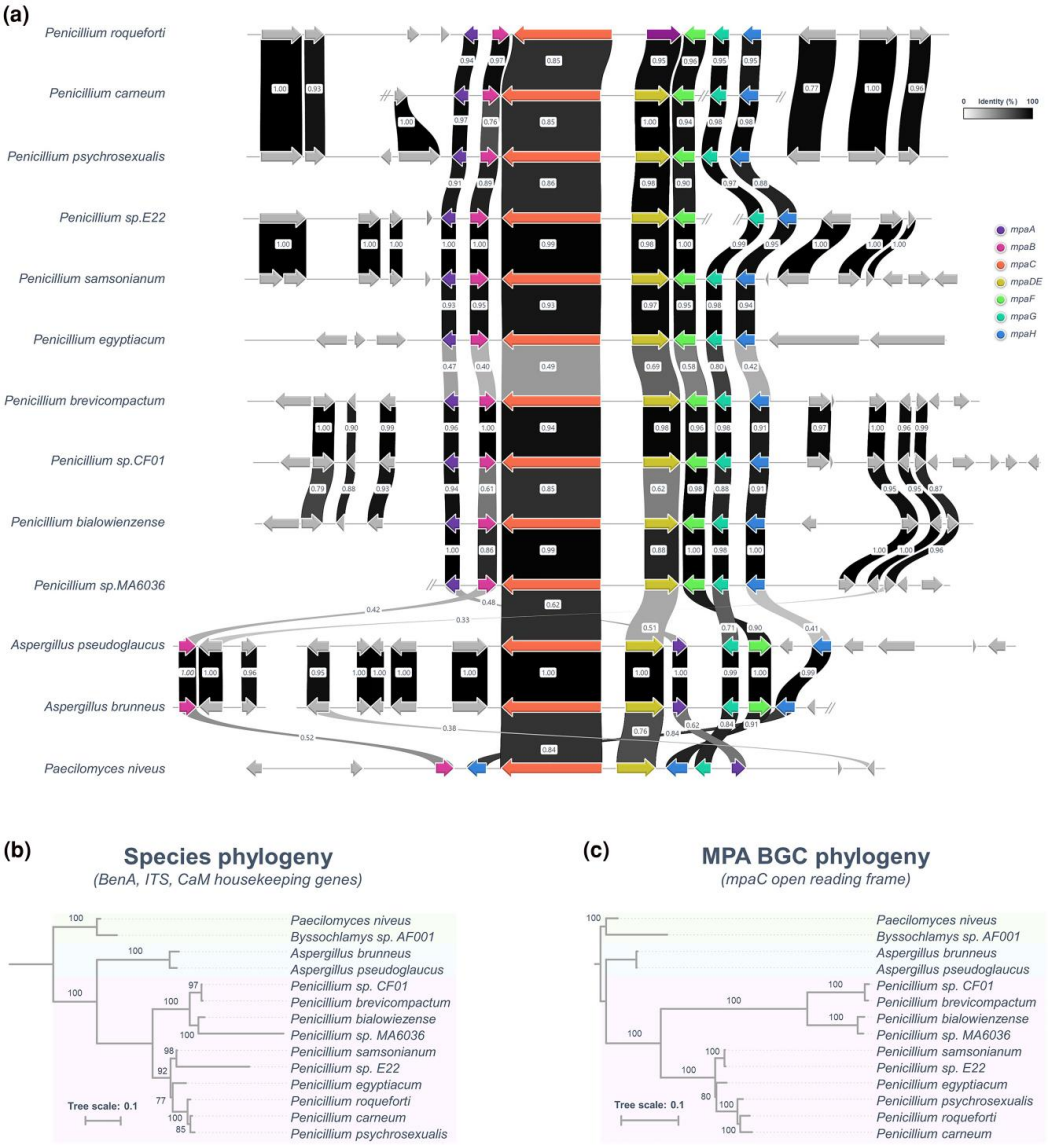
Differential genomic organization of MPA BGCs in *Eurotiales*. a) Syntenic analysis of cluster organization in MPA-producing species, performed as described in Fig. 4. *P. brevicompactum* BGC sequence KM595305 was used as a reference. Sequences were then extracted with bedtools and synteny was constructed with clinker v0.0.23. The figure depicts cluster organization conservation between different species with publicly available sequence data. Identity scores are indicated for each pair of closest homologs. b) Topology of the species tree based on BenA, CaM, and ITS housekeeping genes. c) Tree generated after multiple alignments of *mpaC* open reading frame nucleotide sequence (from start to stop codon including both intron and exon sequences). Values on branches represent bootstrap percentages. Coordinates of cluster sequences in each mold genome are provided in supplementary table S2, Supplementary Material online.

Further phylogenetic analyses based on β-tubulin, RPB2, and BenA sequences provided evidence that *Penicillium* sp. CF01 as well as *Penicillium* sp. MA6036 clusters together with *P. brevicompactum* of section *Brevicompacta* (supplementary fig. S1, Supplementary Material online). Remarkably, our analysis also revealed that *Penicillium* sp. strain 182_6C1 initially attributed to *P. swiecickii* 182_6C1 belongs to *P. samsonianum* (supplementary fig. S1, Supplementary Material online). Importantly, as already observed with *P. brevicompactum* and *P. roqueforti*, the genomic organization of the MPA BGC of *A. pseudoglaucus* E42 differs markedly from those of *Paecilomyces* and *Penicillium* sp. but remains quite similar to that of *A. brunneus* (Fig. 6a).

**Fig. 7.**
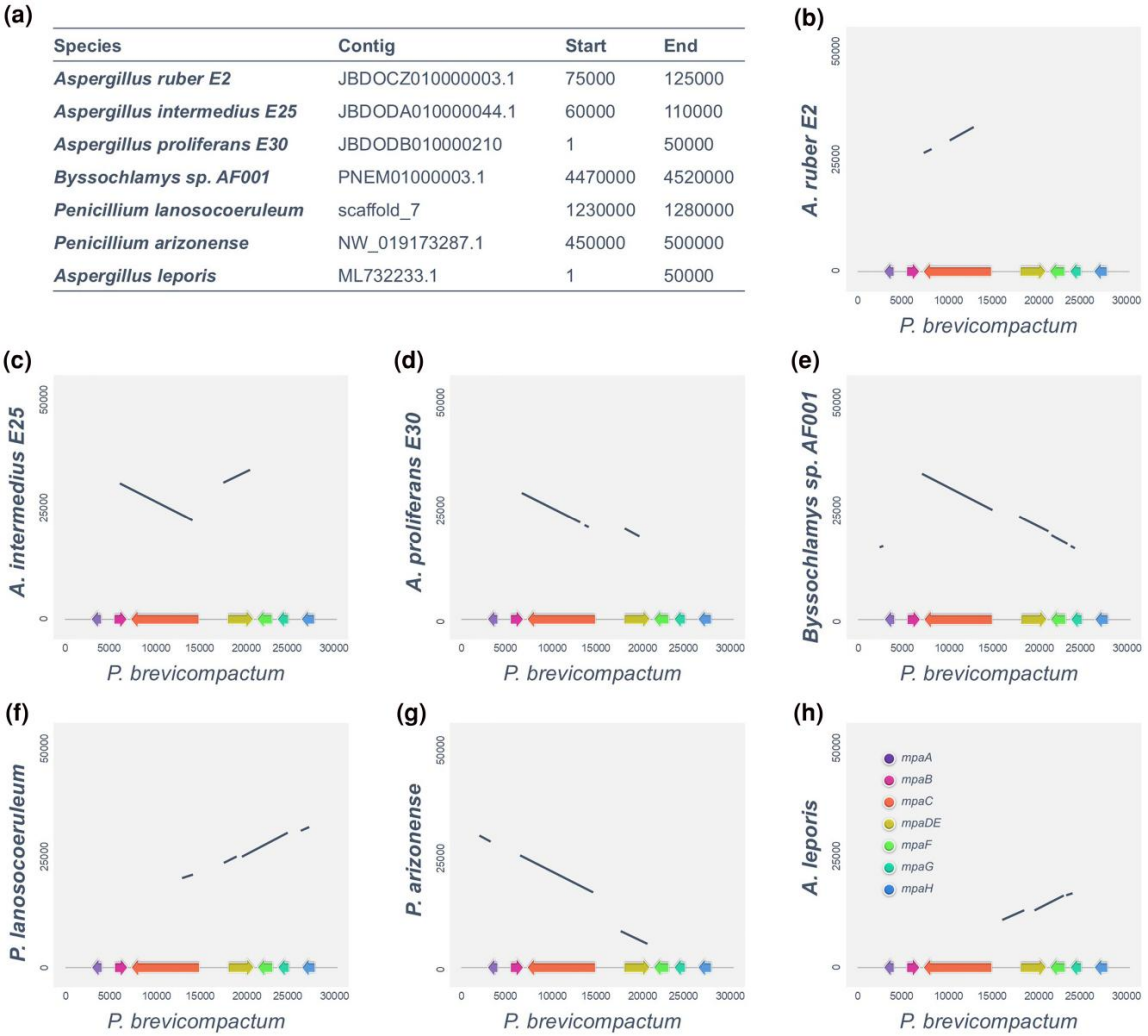
Sequence identity detection between *P. brevicompactum* MPA BGCs and genomic sequences from *Eurotiales* species harboring partial MPA BGCs. a) Start and end coordinates of 50,000 base-analysed sequences (plot ordinate) from the genome of each species. b) *Aspergillus ruber* E2, c) *Aspergillus intermedius* E25, d) *Aspergillus proliferans* E30, e) *Byssochlamys* sp. AF001, f) *Peniciullium lanosocoeruleum*, g) *Penicillium arizonense*, and h) *Aspergillus leporis*. Sequence similarity searches were performed using the KM595305 sequence of *P. brevicompactum* and genomic sequences of species that do not contain an obvious MPA BGC.

Interestingly, we also detected similarity with some genes present in the MPA BGC from *P. brevicompactum* and *P. roqueforti* MPA BGCs in a few other *Eurotiales* species of our dataset (hereafter referred to as “partial” MPA BGCs) (Fig. 7). Indeed, in addition to the herein sequenced species *A. proliferans* E30, a partial MPA BGC was detected in *A. leporis* subgen. *Circumdati* (including *mpaDE*, *mpaF*, and *mpaG*), *P. lanosocoeruleum* (including *mpaA*, *mpaB*, *mpaC*, *mpaDE*, and *mpaF*), *P. arizonense* (including *mpaA*, *mpaC*, and *mpaDE*) but also in *Byssochlamys* sp. AF001 (including *mpaC*, *mpaDE*, *mpaF*, and *mpaG*). However, complementary analyses indicated that these partial BGCs are in fact mostly composed of pseudogenes lacking translation initiation codons and/or introns (Fig. 7).

To eventually gain insight into the evolutionary scenario that could have guided the distribution of MPA BGC in *Eurotiales*, we compared the topologies of the *mpaC* gene genealogies to that of the species tree. As shown in Fig. 6b and c, the topology of the species tree based on BenA, CaM, and ITS housekeeping genes resembles that of the tree generated after multiple alignments of *mpaC* open reading frame nucleotide sequence (from start to stop codon including both intron and exon sequences). This gene tree–species tree reconciliation analyses thus suggest that the history of homologs in the MPA BGC across the *Eurotiales* likely involved vertical gene transfers and multiple losses but no horizontal gene transfer.

### MPA Production in *Eurotiales* Species Harboring Partial or Complete BGC

**Fig. 8.**
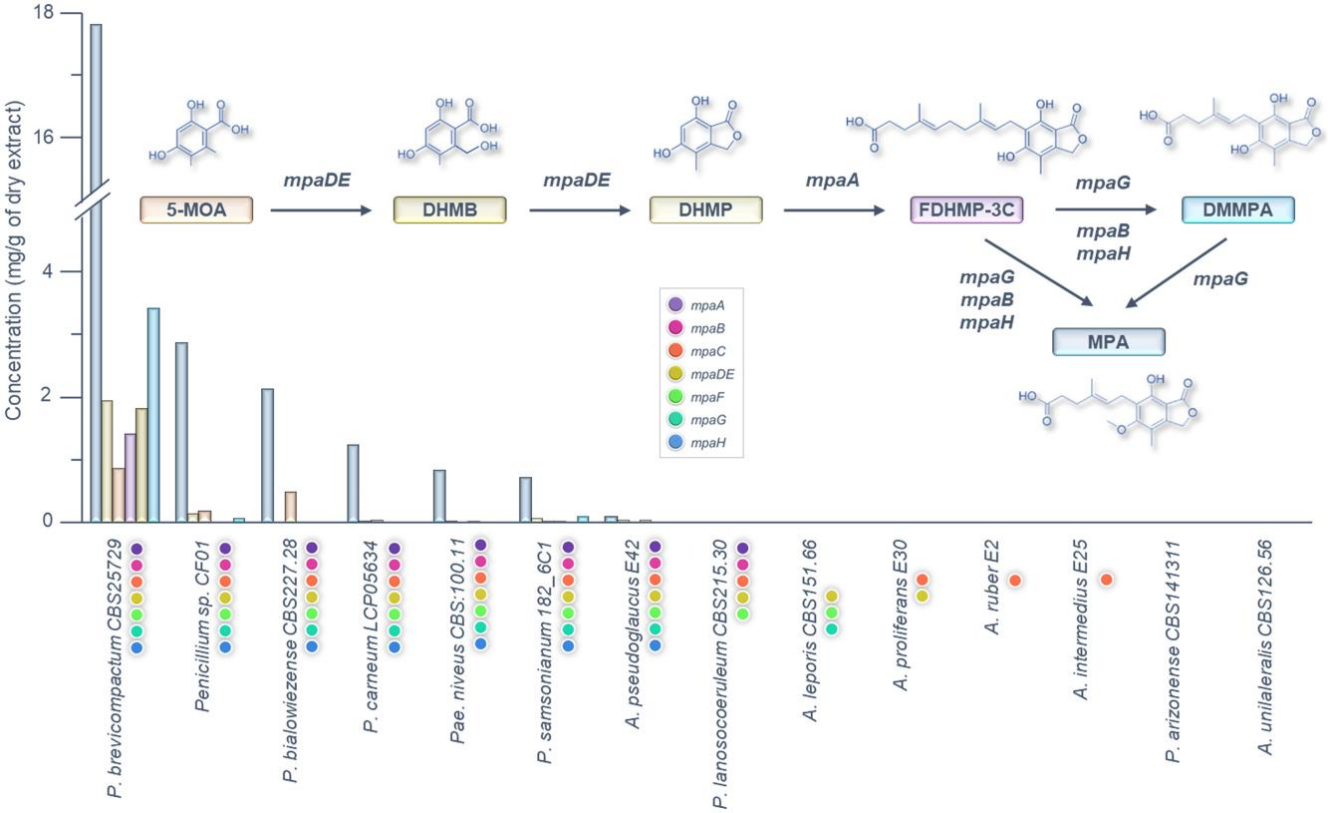
MPA and intermediate compounds production in *Eurotiales* species. MPA and biosynthesis metabolites quantification by UPLC-MS from fungal extracts. Data are expressed in mg/g of dry extract.

We next examined the capacity of some available species/strains harboring a predicted partial or complete BGC to be effective MPA or MPA biosynthetic intermediate producers. Results shown in Fig. 8 demonstrate that species displaying a complete BGC actually accumulate MPA and some intermediates. This includes species previously identified as MPA producers like *P. brevicompactum*, *P. carneum*, *P. bialowiezense*, *Paecilomyces niveus*, and *A. pseudoglaucus* E42 (Puel et al. 2005; Hansen et al. 2011a; Mouhamadou et al. 2017; Houbraken et al. 2020; Garello et al. 2024) as well as the two strains, *Penicillium* sp. CF01 and *P. samsonianum* 182_6C1, described in this study and reported for the first time, as MPA producers. In our experimental conditions, the best MPA producer was *P. brevicompactum*, while the lowest MPA production was detected in *A. pseudoglaucus* E42. These results thus demonstrate that MPA BGCs detected sporadically in some *Eurotiales* species are all functional. As expected, we never detected any MPA traces or intermediates in the tested strains harboring partial MPA BGCs.

### Correlating MPA Production and Resistance in *Eurotiales*

Plants, algae, fungi, and bacteria producing toxic compounds involved in interspecies chemical warfare have concomitantly evolved adapted defence mechanisms for preventing self-intoxication (Demain and Fang 2000). In this respect, *P. brevicompactum* remains indeed a prominent example since the mold both produces MPA and is strongly resistant to this drug. To gauge the relationship between MPA production and resistance in *Eurotiales*, we determined the MPA susceptibility in a panel of species including both MPA producers and nonproducers. We calculated the percentage of growth inhibition by measuring radial growth over a 7-d period (supplementary fig. S2, Supplementary Material online). As indicated in Table 2, all MPA producers including *P. brevicompactum*, *P. roqueforti*, *P. carneum*, *P. samsonianum*, *Penicillium* sp. CF01, *Paecilomyces niveus*, and *A. pseudoglaucus* E42 were found resistant to MPA. As initially observed in *P. brevicompactum,* all these species harbor two distinct copies of the gene encoding the MPA target protein (IMPDH) i.e. IMPDH-A shared by all fungi and IMPDH-B (MpaF) located within the BGC (Hansen et al. 2012b). A phylogenetic tree of the distinct IMPDH-encoding genes present in the genomes of the tested mold species is provided in supplementary fig. S3, Supplementary Material online. Noteworthy, three out of the six MPA-producing species display a hydroxylated amino acid (serine or threonine) at position 267 of the predicted IMPDH-A protein (supplementary fig. S4, Supplementary Material online), a feature previously demonstrated to participate in MPA resistance in *S. cerevisiae*, *P. brevicompactum*, *C. albicans*, and *M. guilliermondii* (Jenks and Reines 2005; Köhler et al. 2005; Defosse et al. 2016; Freedman et al. 2020). Otherwise, most of the tested nonproducing-MPA *Eurotiales* species were found to be susceptible to MPA (e.g. *A. ruber* E2, *A. intermedius* E25, *A. nidulans*, *A. fumigatus*, *A. flavus*, *A. oryzae*, *A. sydowii*, *A. terreus*, *A. glaucus*, *P. chrysogenum*, and *A. candidus*) while a restricted number of molds was shown to be resistant (*A. proliferans* E30, *A. clavatus*, and *A. versicolor*) (Table 2). The underlying mechanisms of resistance in nonproducing species did not follow any identifiable pattern. For instance, nonproducing and MPA-susceptible species display either one (e.g. *A. ruber* E2, *A. intermedius* E25, *A. nidulans*, *A. fumigatus*, *A. flavus*, *A. oryzae*, *A. sydowii*, *A. terreus*, and *A. glaucus*) or two identical copies (*P. chrysogenum* and *A. candidus*) of IMPDH-A protein with either an alanine or a hydroxylated amino acid at position 267 (Table 2). Besides, nonproducing and MPA-resistant species (*A. proliferans* E30, *A. clavatus*, and *A. versicolor*) harbor a single copy of the *impdh* gene with a hydroxylated amino acid at position 267 (Table 2). Taken together, these results suggest that (i) the presence of the atypical copy of IMPDH gene (*impdh-B* or *mpaF*, which always harbors an alanine at position 267 of the predicted protein) is associated with MPA resistance, (ii) no clear correlation between MPA susceptibility profiles and *impdh-A* copy number, (iii) the absence of links between resistance to MPA and the substitution from alanine to a hydroxylated amino acid at position 267 in the predicted IMPDH-A sequence. Based on the current and previous findings, we expect that MPA resistance is due to a combination of molecular mechanisms likely involving *impdh* gene(s) dosage, *impdh* gene(s) expression level(s), and the nature of the amino acid at position 267 in the IMPDH-A protein (Hansen et al. 2011a, 2012b; Freedman et al. 2020).

**Table 2:** MPA production, IMPDH genes, and MPA resistance in a selection of *Eurotiales* molds

Species	MPA prod.	IMPDH-A a.a. 267	IMPDH-B a.a. 267	Growth % (7 d)
*Penicillium brevicompactum*	+	S	A	78.79 (R)
*Penicillium* sp. CF01	+	S	A	70.59 (R)
*Penicillium carneum*	+	A	A	85.96 (R)
*Paecilomyces niveus*	+	A	A	91.67 (R)
*Penicillium samsonianum*	+	A	A	68.97 (R)
*Aspergillus pseudoglaucus* E42	+	T	A	60 (R)
*Aspergillus proliferans* E30	−	T	−	64.52 (R)
*Aspergillus ruber* E2	−	T	−	41.38 (S)
*Aspergillus intermedius* E25	−	A	−	0 (S)
*Aspergillus nidulans*	−	A	−	49.18 (S)
*Aspergillus fumigatus*	−	A	−	36.92 (S)
*Aspergillus flavus*	−	A	−	32.94 (S)
*Aspergillus oryzae*	−	A	−	25 (S)
*Aspergillus clavatus*	−	S	−	70.59 (R)
*Aspergillus versicolor*	−	S	−	67.86 (R)
*Aspergillus sydowii*	−	S	−	47.37 (S)
*Aspergillus terreus*	−	S	−	16.67 (S)
*Aspergillus glaucus*	−	T	−	0 (S)
*Penicillium chrysogenum*	−	A	A	37.14 (S)
*Aspergillus candidus*	−	A	A	33.85 (S)

MPA production referred to results shown in Fig. 8. Categorization of IMPDH versions was determined following a phylogenetic analysis shown in supplementary fig. S3, Supplementary Material online. Amino acid at position 267 was determined with a multiple alignment shown in supplementary fig. S4, Supplementary Material online. Growth % was determined after 168 h in the presence of 200 µg/mL MPA. The growth kinetic curves are provided in supplementary fig. S2, Supplementary Material online. Strains considered resistant are indicated with (R) and susceptible with (S) in the growth % column.

A, alanine; S, serine; T, threonine.

## Discussion

Fungi play pivotal roles in ecosystems and have garnered attention for their ability to produce bioactive secondary metabolites with potential applications in medicine, agriculture, and industry. Cumulative evidence in the field revealed that genes encoding enzymes catalyzing the production of these secondary metabolites are organized in BGCs (Rokas et al. 2018). However, our understanding of the biology and evolution of secondary metabolic pathways, particularly those encoding less well-studied compounds, remains limited. MPA is a terpene-PK hybrid molecule that has been widely used for decades in human therapy as an immunosuppressant (Bentley 2000). In this study, we aimed to understand the evolution and distribution of fungal MPA BGCs. We also sought to unravel the genetic basis of fungal resistance to MPA, thereby contributing to a broader understanding of the ecological significance of fungal secondary metabolites.

### Some Filamentous Fungi Belonging to the *Aspergillus* Genus Have Functional MPA BGCs

Up to now, MPA production has been detected in a few *Aspergillus* species, but no studies have addressed the underlying modalities in this genus. By sequencing the genome of four *Aspergillus* strains, we revealed a unique and functional MPA BGC in *A. pseudoglaucus*. The genomic organization of the newly identified MPA BGC in *A. pseudoglaucus* markedly differed from that of *P. brevicompactum* and *P. roqueforti* (Hansen et al. 2011b; Regueira et al. 2011; Del-Cid et al. 2016; Gillot et al. 2017). The presence of a partial MPA BGC in several species closely related to *A. pseudoglaucus*, such as *A. proliferans*, *A. intermedius*, and *A. ruber*, suggests that the MPA BGC originated once in an *Aspergillus* ancestor and was subsequently retained in some species but lost in others. Such patterns of BGC rearrangement and repeated loss are often observed in the fungal kingdom (Kjærbølling et al. 2020). For example, recent analyses of synteny across genomes of bioluminescent mushroom species have revealed how the luciferase BGC was frequently rearranged and lost (Ke et al. 2020). Similar patterns have also been observed in bacterial and plant BGCs (Cimermancic et al. 2014; Liu et al. 2020a, 2020b; Yang et al. 2021).

### Only a Few Filamentous Fungi Have Functional MPA BGCs

Despite the availability of thousands of fungal genomes, the presence of MPA BGCs has never been systematically analyzed in the fungal kingdom. Our phylogenomic analysis of 479 *Eurotiales* genomes revealed the presence of complete and functional MPA BGCs only in a few *Penicillium* species and related taxa. We found that the evolutionary history of a key biosynthetic gene (i.e. *mpaC*) is consistent with the species phylogeny. Thus, horizontal gene transfer and/or introgression are unlikely and the pattern of distribution of the MPA BGC is best explained by repeated losses of the cluster in the *Penicillium* genus.

### Some Filamentous Fungi Have Partial MPA BGCs

Our analysis also sheds light on the presence of partial MPA BGCs in several *Eurotiales* species. Indeed, in addition to the herein sequenced species *A. proliferans*, *A. intermedius*, and *A. ruber*, sequence similarity was detected between *P. brevicompactum* MPA BGC and some genomic regions in *A. leporis*, *P. lanosocoeruleum*, *P. arizonense*, and *Byssochlamys* sp. Additional analysis revealed that these partial BGCs are in fact mostly composed of genes that are in the process of pseudogenization, suggesting that MPA biosynthesis is likely not occurring in these species. Of note, based on homology to known MPA BGCs, a predicted partial BGC in the genome of *P. arizonense* CBS 141311 was connected to austalides (Grijseels et al. 2016; Awakawa et al. 2023), which are a family of related meroterpenoid metabolites. The genomic and chemical similarity of the MPA and austalides biosynthetic pathways raises the hypothesis that they have a common origin. Therefore, another, not mutually exclusive, possibility is that these partial BGCs may reflect metabolic diversification and the emergence of new biosynthetic pathways for secondary metabolites, as observed in plants (Lichman et al. 2020; Liu et al. 2020a).

### MPA Biosynthesis Likely Appeared Early in the Evolution of Filamentous Fungi

Interestingly, this study also revealed that a 7-gene BGC for MPA biosynthesis occurs and is functional in *Paecilomyces niveus*, a species that is distantly related to the *Aspergillus* and *Penicillium* genera. Gene organization of the MPA BGC of *P. niveus* also differs from those of *P. brevicompactum, P. roqueforti,* and *A. pseudoglaucus*. The presence of a complete and functional MPA BGC in *P. niveus* suggests that MPA biosynthesis likely appeared early in the evolution of *Eurotiales* and was then repeatedly lost in most of the species but retained in a few sections. As mentioned previously, this pattern of repeated loss has been observed in several other fungal BGCs (Robey et al. 2021; Balamurugan et al. 2024) and plant secondary metabolic pathways (Liu et al. 2020a; Wang et al. 2023; Cuello et al. 2024).

### MPA Resistance Likely Involves Different Molecular Determinants in Filamentous Fungi

In the second part of this study, we examined the link between MPA production and resistance in *Eurotiales*. Our analysis revealed that the *mpaF* resistance gene exists in the majority of species belonging to *Penicillium* subgen. *Penicillium*. These findings reinforce the hypothesis made by Hansen et al. (2011a) stating that duplication of *impdh* gene occurred at or near the origin of *Penicillium* subgen. *Penicillium*. Above all, our analysis suggests that (i) the presence of a unique copy *impdh-B* gene harboring an alanine at position 267 of the predicted protein is associated with MPA susceptibility, (ii) no clear correlation between MPA susceptibility profiles and *impdh* copy number, and (iii) the absence of correlation between resistance to MPA and the presence of a hydroxylated amino acid at position 267 in the sequence of the predicted IMPDH. Thus, as recently revealed in *P. brevicompactum*, MPA resistance likely stems from a combination of molecular determinants involving *impdh* copy number, *impdh* gene(s) expression level(s), and the nature of the amino acid at position 267 in the IMPDH-A deduced protein (Hansen et al. 2011a, 2012b; Freedman et al. 2020).

## Concluding Remarks

In conclusion, this study provides insight into the origin and evolution of the biosynthesis of the important secondary metabolite MPA in filamentous fungi. A complete and functional MPA BGC was detected not only in a few *Penicillium* and *Aspergillus* species but also in other more distant branches of the *Eurotiales* phylogeny. This may indicate that a few different fungal species in diverse lineages use MPA for interspecies communication/chemical warfare. Collectively, our work strengthens the need for continuing and broadening efforts to evaluate the evolution of fungal MPA biosynthesis. While the origin of this gene cluster is still enigmatic, future studies across *Eurotiales* could shed light on the nature of the evolutionary forces that drove the formation, maintenance, and decay of the MPA BGC (Rokas et al. 2018).

## Material and Methods

### Strains and Culture

The strains of xerophilic *Aspergillus* were collected from different environments as described in Mouhamadou et al. (2017). These strains are preserved in the fungal collection of Caen University (CFU). *Penicillium carneum* LCP05634 from the collection of the French National Museum of Natural History was kindly provided by Pr. J. Dupont. *Penicillium samsonianum* strain 182_6C1 (see below) was kindly provided by Dr. Olivia L. Romero Olivares from New Mexico State University, USA. *Paecilomyces niveus* CBS100.11 was obtained from the culture collection of Université de Bretagne Occidentale, France. *Penicillium* sp. CF01 (see below) of section *Brevicompacta* was isolated from a plastic waste recycling plant in Colfelice, Italy. The strain is preserved in the public mycological collection of Mycotheca Universitatis Taurinensis (as MUT 6482). All the other strains were available in-house (Angers University Hospital Centre, France). All strains were cultivated on CY20S medium (Czapek Yeast autolysate Agar supplemented with 20% sucrose).

### Sequencing and Genome Assembly

For the production of short-read sequences of E2, E25, E30, and E42 strains, the plate scrapped material was grounded in liquid nitrogen using mortar and pestle. Genomic DNA was then extracted using the Nucleospin Plant II Kit from Machery-Nagel following the manufacturer's instructions. DNA was sequenced by Eurofins genomics on the Illumina MiSeq platform using 300 base pairs paired-end flow cell after standard genomic library preparation. For E42 long-read sequence production, 200 mL of liquid CY20S (Czapek Yeast autolysate supplemented with 20% sucrose) was inoculated with 2 × 10^7^ spores for 4 h (allowing their germination). Nascent filaments were harvested by Miracloth filtration (Merck-Millipore). Germ tubes were incubated with cell-wall degrading enzyme Glucanex (Sigma-Aldrich) for 4 h at 37 °C and 120 rpm. Sorbitol-Tris buffer allows retrieval of protoplast. High molecular weight DNA was then purified from protoplast using the Monarch HMW DNA extraction kit (NEB). Singleplex DNA library was prepared with the Ligation Sequencing Kit SQK-LSK110 (Oxford-Nanopore) and sequenced on FLO-MIN 106D flow cell (Oxford-Nanopore).

Short reads were analyzed with FastQC (Andrews S. 2010). Trimmomatic_0.39 (Bolger et al. 2014) was used to trim reads with TrueSeq3-PE.fa file for Illumina adapter removal (2:30:10). Quality score set at 30 was used for leading and trailing; 5 nucleotides window size with 20 required quality was used for filtering. Finally, reads shorter than 50 nucleotides were removed. E2, E25, and E30 genomes were then assembled with Spades_3.13 (Prjibelski et al. 2020) using both pairing and unpairing filtered short reads, with default parameters. E42 was assembled with Spades_3.13 (Antipov et al. 2016) using both pairing and unpairing filtered short reads plus nanopore long reads with *-careful* option and the following kmer length 21, 33, 55, 77, 99, and 127, as suggested by Spades developers.

### Bioinformatic Analysis

E2, E25, E30, and E42 genome annotations were performed by Augustus 3.5.0 with the closest available species (i.e. *Aspergillus fumigatus*) as reference species (Stanke et al. 2008). *Eurotiales* genomes were retrieved from the NCBI/Assembly database or from the JGI/Genomeportal, including the genome assembly ASM525074v2 of *Penicillium* sp. CF01 (bioproject PRJNA427105, NCBI). Global genome analysis for BGC was performed with antiSMASH 6.0 (Blin et al. 2021). The MPA BGC was identified via Blast_2.5.0 analysis, using HQ731031 from *Penicillium brevicompactum* and KU234530 from *Penicillium roqueforti* as query sequences (Zhang et al. 2000). After identification, clusters from *P. niveus* and *A. pseudoglaucus* were also used as a query sequence to enlarge the analysis. BGC synteny was analyzed with the clinker software (Gilchrist and Chooi 2021). IMPDH-A and IMPDH-B alignments were performed using CLUSTAL O (1.2.4) (Madeira et al. 2022). We inferred the maximum-likelihood tree using IQtree with 1000 bootstraps (-nt AUTO -m TEST -bb1000) (Nguyen et al. 2015). The cladogram and phylogenetic tree were graphed using iTOL (Letunic and Bork 2021). Sequence conservation on degenerated BGCs was analyzed with LASTZ_1.04.15. (Harris 2007).

### MPA Growth Inhibition

Spores reaching maturity were harvested from culture plates and resuspended in sterile water before counting in a Neubauer cell chamber. For every species considered, 5 µL of a 10^5^ spores/mL solution was seeded at the center of CY20S medium Petri dish, containing 200 µg/mL of MPA. Plates were incubated at 25 °C for 7 d. Radial growth was calculated by the measurement of colony diameter at 72, 96, 120, 144, and 168 h.

### MPA and MPA Intermediate Detection

The production of MPA was investigated using ultra-high performance liquid chromatography coupled with mass spectrometry (UPLC-MS). The extracts were resuspended in a solution of water-acetonitrile (9:1, v/v). The UPLC-MS method was adapted from Zhang et al. (2019). The UPLC-MS system was composed of an ACQUITY UPLC system coupled to a photodiode array detector (PDA) and a Xevo TQD mass spectrometer (Waters, Milford, MA) equipped with an electrospray ionization (ESI) source controlled by Masslynx 4.1 software (Waters, Milford, MA). A Waters Acquity HSS T3 C18 column (150 × 2.1 mm, 1.8 μm) achieved the analytes separation with a flow rate of 0.4 mL/min at 55 °C. The injection volume was 5 μL. The mobile phase was composed of 0.1% formic acid in water (solvent A) and 0.1% formic acid in acetonitrile (solvent B). The chromatographic separation was performed using an 18-min linear gradient from 5% to 60% solvent B, followed by washing and column reconditioning in 8 min. MS detection was performed in both positive and negative modes in full scan mode. The capillary voltage was 3,000 V, and sample cone voltages were 30 and 60 V. The cone and desolvation gas flow rates were 60 and 800 L/h, respectively. For relative quantification, UPLC-MS analyses were performed in selected ion monitoring (SIM) mode for the following compounds: DHMP [M-H]^−^ at *m/z* 179 (RT = 6.28 min), 5-MOA [M-H]^−^ at *m/z* 181 (RT = 8.37), DMMPA [M-H]^−^ at *m/z* 305 (RT = 11.88 min), MPA [M-H]^−^ at *m/z* 319 (RT = 14.15 min), and FDHMP-3C [M-H]^−^ at *m/z* 373 (RT = 16.72 min). The resulting SIM chromatograms were integrated using the ApexTrack algorithm with a mass window of 0.1 Da and relative retention time window of 0.2 min followed by Savitzky-Golay smoothing (iteration = 1, width = 1) using Targetlynx software. The resulting peak integrations and retention times were finally visually examined.

## Supplementary Material

evaf039_Supplementary_Data

## Data Availability

The data underlying this article are available in NCBI, at GenBank database (National Center for Biotechnology Information, NCBI, https://www.ncbi.nlm.nih.gov/). Genome data for *A. ruber* E2, *A. intermedius* E25, *A. proliferans* E30, and *A. pseudoglaucus* E42 relate to the bioproject PRJNA802395. Raw reads files have been deposited on NCBI/SRA, and assembled genomes are available on NCBI/Assembly.
