# Supplementary material for: Tracing the Origin and Evolution of the Fungal Mycophenolic Acid Biosynthesis Pathway
Source: Genome Biol Evol. 2025 Mar 7;17(3):evaf039. doi: 10.1093/gbe/evaf039 (PMC11934065; doi:10.1093/gbe/evaf039)
Supplement: evaf039_Supplementary_Data [file evaf039_supplementary_data.docx]

**Supplementary materials**

_______________________________________________________________________________

**Tracing the Origin and Evolution of the Fungal Mycophenolic Acid Biosynthesis Pathway**

Baptiste Bidon^1,2^, Hajar Yaakoub^1,3^, Arnaud Lanoue^4^, Antoine Géry^5^, Virginie Séguin^5^, Florent Magot^4^, Claire Hoffmann^6,7^, Vincent Courdavault^4^, Jean-Philippe Bouchara^1^, Jean-Pierre Gangneux^8,9^, Jens C. Frisvad^10^, Antonis Rokas^11,12^, Gustavo H. Goldman^13^, Solène Le Gal^6,7^, Gilles Nevez^6,7^, Domenico Davolos^14^, David Garon^5,*^, Nicolas Papon^1,*^

^1^ Univ Angers, Univ Brest, IRF, SFR ICAT, F-49000, Angers, France.

^2^ Centre for Genomics and Precision Medicine, National Taiwan University, Taipei, Taiwan (R.O.C.).

^3^ Nantes Université, INRAE UMR-1280 PhAN, F-44000, Nantes, France.

^4^ Université de Tours, BBV EA2106, Tours, France.

^5^ ABTE EA 4651-ToxEMAC, Normandie Université, UNICAEN, UNIROUEN, Caen, France.

^6^ Univ Brest, Univ Angers, IRF, SFR ICAT, F-49000, Brest, France.

^7^ Parasitology-Mycology Unit, Brest University Hospital, Brest, France.

^8^ Univ Rennes, CHU Rennes, Inserm, EHESP, Irset (Institut de recherche en santé, environnement et travail)-UMR_S 1085, Rennes, France.

^9^ Parasitology-Mycology Unit, Rennes University Hospital, European Excellence Center in Medical Mycology (ECMM EC), Centre National de Référence pour les mycoses et antifongiques-laboratoire associé Aspergilloses chroniques (CNRMA- LA AspC), Rennes, France.

^10^ Department of Biotechnology and Biomedicine, Technical University of Denmark, Kongens Lyngby, Denmark

^11^ Department of Biological Sciences, Vanderbilt University, Nashville, TN, USA.

^12^ Vanderbilt Evolutionary Studies Initiative, Vanderbilt University, Nashville, TN, USA.

^13^ Faculdade de Ciências Farmacêuticas de Ribeirão Preto, Universidade de São Paulo, Ribeirão Preto ,and National Institute of Science and Technology in Human Pathogenic Fungi, Brazil.

^14^ Department of Technological Innovations and Safety of Plants, Products and Anthropic Settlements (DIT), INAIL, Research Area, Rome, Italy.

* Correspondence: [david.garon@unicaen.fr](mailto:david.garon@unicaen.fr) ; [nicolas.papon@univ-angers.fr](mailto:nicolas.papon@univ-angers.fr)


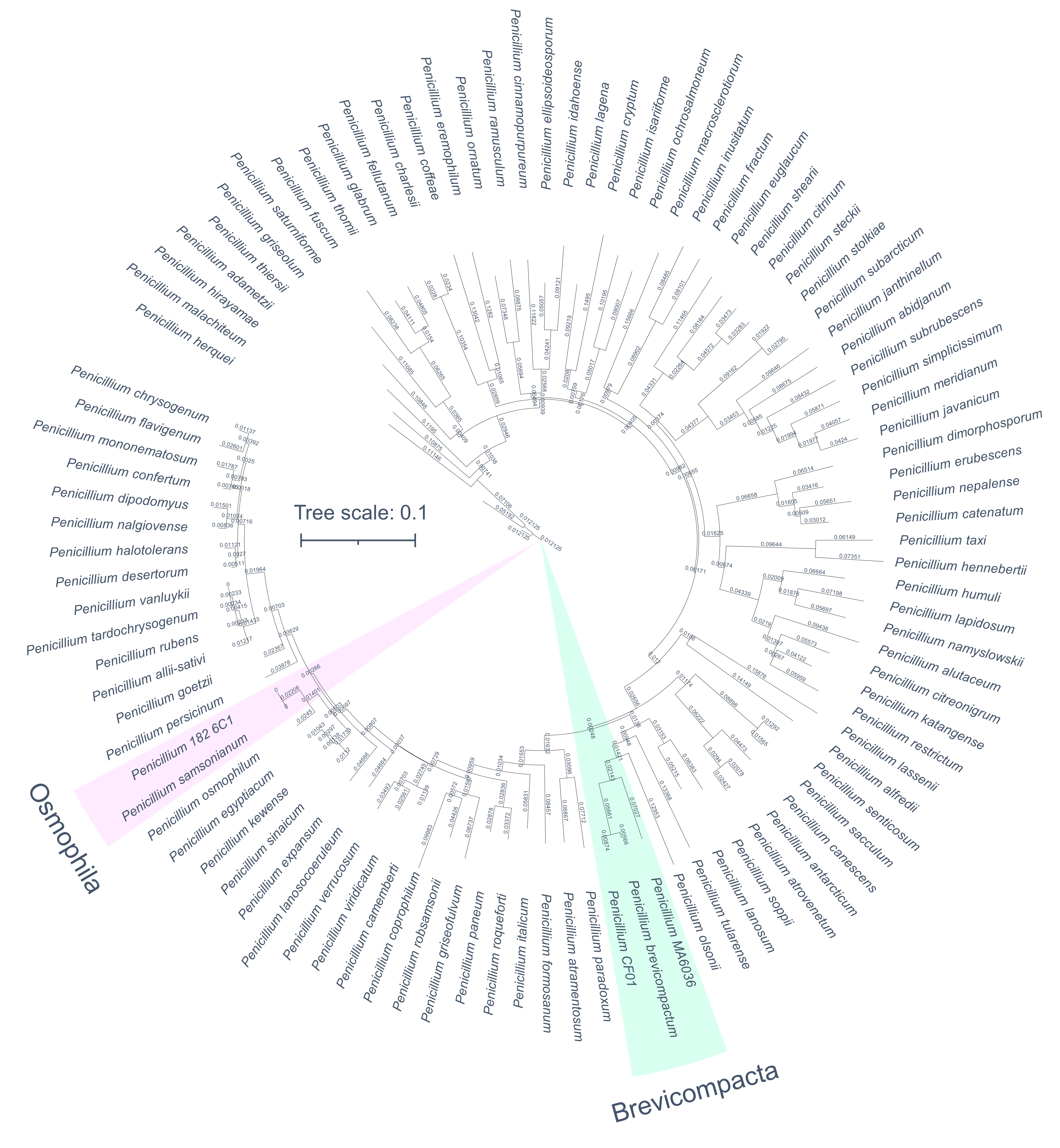


**Supplemental Figure S1. Phylogeny of *Penicillium* sp. based on sequence analysis of β-tubulin, RPB2, and BenA genes.** This suggests that *Penicillium* sp. CF01 as well as *Penicillium* sp. MA6036 cluster together with *P. brevicompactum* and that *Penicillium* strain 182_6C1 first ascribed as *P. swiecickii* belongs to *Penicillium* *samsonianum.* Values represent phylogenetic branch lengths.


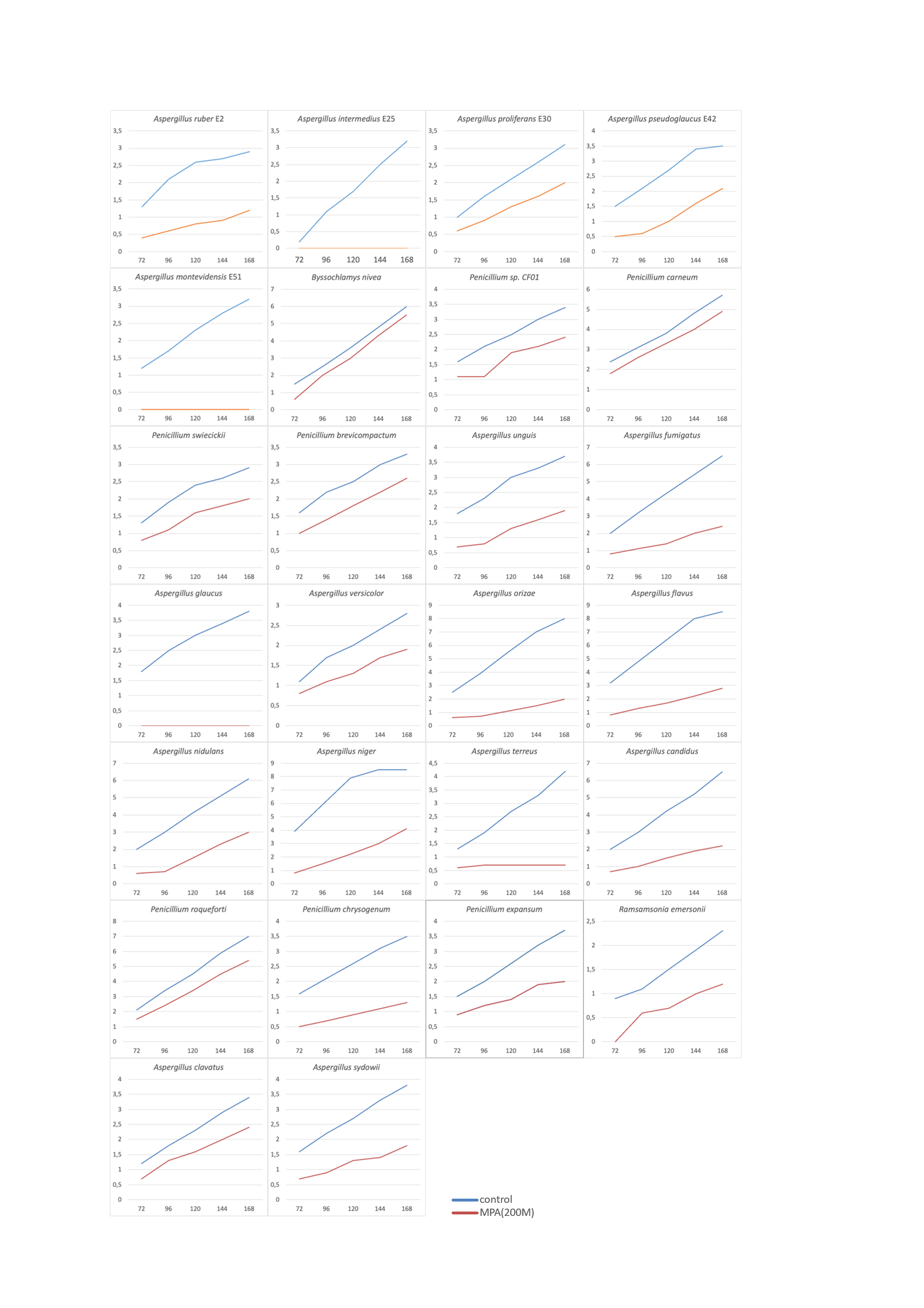


**Supplemental Figure S2. Mycophenolic acid (MPA) susceptibility of a panel of molds.** Spores from culture plates were harvested and suspended in sterile water before counting in a Neubauer cell chamber. For each species, 5 µL of a 10^5^ spores/mL solution was cultivated on CY20S medium, containing 200 µg/mL of mycophenolic acid. Plates were incubated at 25°C for 7 days. Colony diameter was measured at 72h, 96h, 120h, 144h, and 168h.

*
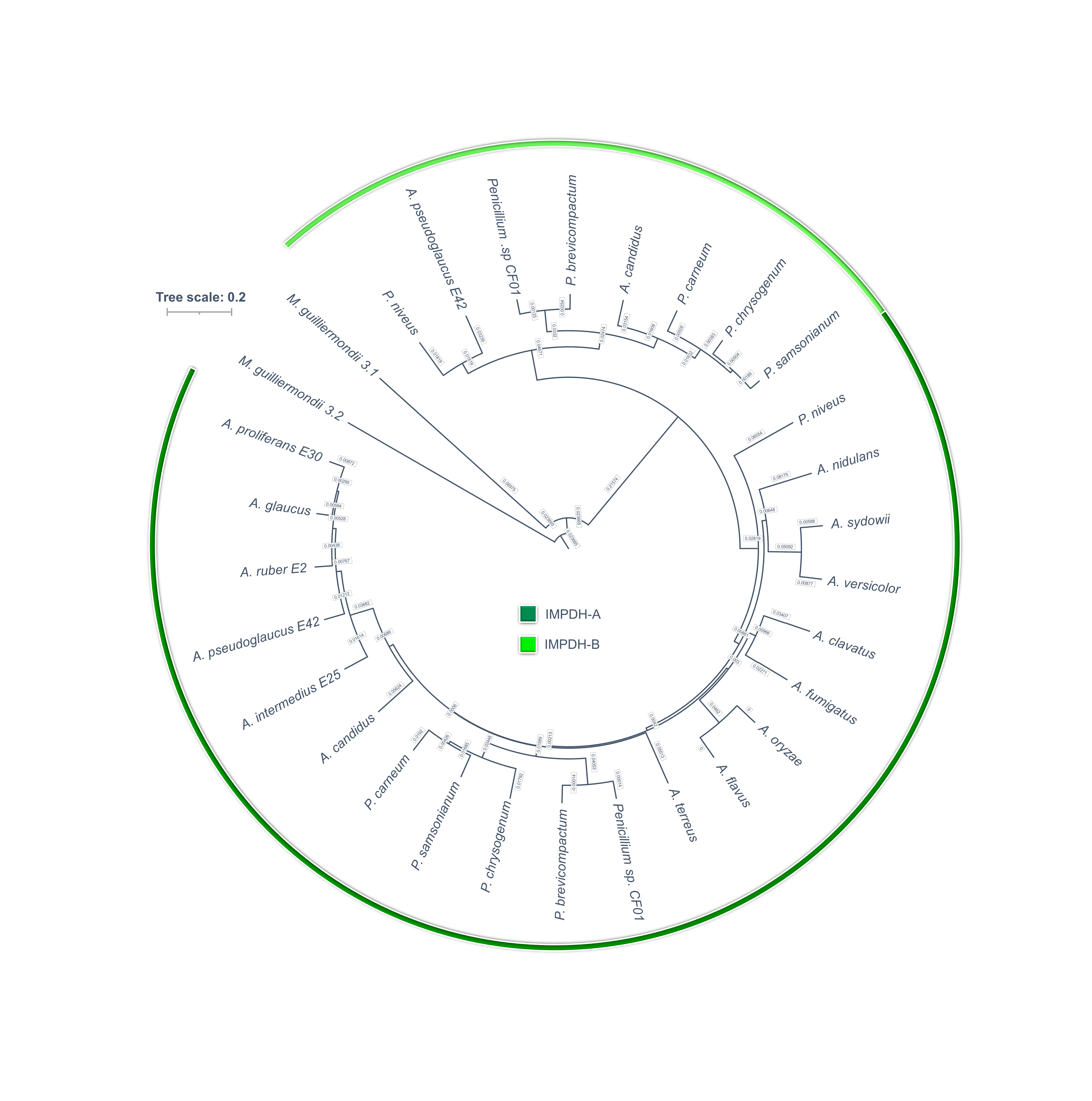
*

**Supplemental Figure S3. Phylogeny of the distinct inosine monophosphate dehydrogenase (IMPDH)-encoding genes found in the selection of *Eurotiales* molds.** Maximum likelihood tree of IMPDH protein sequences from species analysed in Table 2. The two copies characterised in the yeast *M. guilliermondii* are used as outgroup sequences to root the tree (Defosse et al., 2016). IMPDH-A (regular) IMPDH-B (mpaF) form two distinct branches. Values represent phylogenetic branch length.


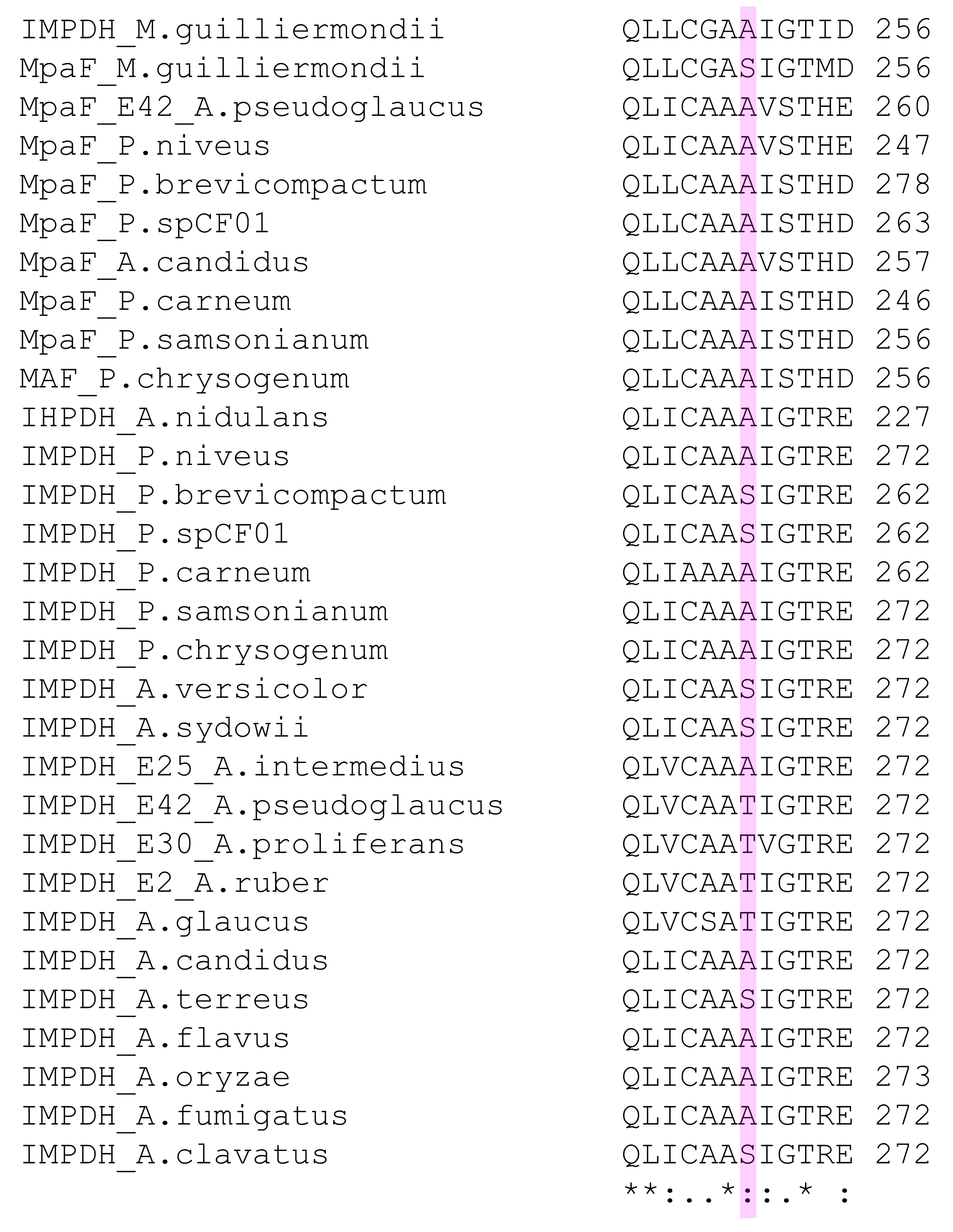


**Supplemental Figure S4. Multiple alignment of partial inosine monophosphate dehydrogenase (IMPDH) sequences retrieved from various fungal species.** The specific residue involved in MPA susceptibility is indicated in pink.

**Supplemental Table 1. List of genomes analysed in this study.**

| *Organism Name* | Assembly Accession | Assembly Name | Organism Taxonomic ID | Organism Infraspecific Names Strain | Organism Infraspecific Names Ecotype | Organism Infraspecific Names Isolate | Annotation Name | Assembly Stats Total Sequence Length | Assembly Stats Total Number of Chromosomes | Assembly Level | Assembly Release Date | WGS project accession | Assembly Stats Contig N50 | Assembly Stats Scaffold N50 | Assembly Stats Number of Scaffolds | Assembly Sequencing Tech | Assembly Submitter | Assembly BioProject Accession | Assembly BioSample Accession | Annotation Count Gene Total | Annotation Count Gene Protein-coding | Annotation Count Gene Pseudogene |
| --- | --- | --- | --- | --- | --- | --- | --- | --- | --- | --- | --- | --- | --- | --- | --- | --- | --- | --- | --- | --- | --- | --- |
| *Aspergillaceae sp. M1327* | GCA_023626435.1 | ASM2362643v1 | 2940351 | PF1803 |  |  |  | 27695660 |  | Scaffold | 01/06/2022 | JAMAGX01 | 378393 | 556291 | 309 | Illumina HiSeq | Dalian Medical University | PRJNA833221 | SAMN27963490 |  |  |  |
| *Aspergillaceae sp. PF1803* | GCA_023626575.1 | ASM2362657v1 | 2940349 | PT4103 |  |  |  | 40239996 |  | Scaffold | 01/06/2022 | JAMAGZ01 | 145836 | 196499 | 1741 | Illumina HiSeq | Dalian Medical University | PRJNA833221 | SAMN27963486 |  |  |  |
| *Aspergillaceae sp. S1602* | GCA_023626355.1 | ASM2362635v1 | 2940352 | S1701 |  |  |  | 33728083 |  | Scaffold | 01/06/2022 | JAMAGW01 | 510485 | 715071 | 478 | Illumina HiSeq | Dalian Medical University | PRJNA833221 | SAMN27963491 |  |  |  |
| *Aspergillaceae sp. S1701* | GCA_023626415.1 | ASM2362641v1 | 2940350 | PF2412A |  |  |  | 35144504 |  | Scaffold | 01/06/2022 | JAMAGY01 | 413082 | 618233 | 226 | Illumina HiSeq | Dalian Medical University | PRJNA833221 | SAMN27963489 |  |  |  |
| *Aspergillus aculeatinus CBS 121060* | GCF_003184765.1 | Aspacu1 | 1448322 | CBS 121060 |  |  | Annotation submitted by DOE Joint Genome Institute | 36471649 |  | Scaffold | 04/06/2018 | PSTE01 | 200731 | 493913 | 121 | Illumina | DOE Joint Genome Institute | PRJNA235093 | SAMN05660730 | 12354 | 12028 | 3 |
| *Aspergillus aculeatus ATCC 16872* | GCF_001890905.1 | Aspac1 | 690307 | ATCC 16872 |  |  | Annotation submitted by JGI | 35424414 |  | Scaffold | 08/12/2016 | MRCK01 | 256127 | 1634707 | 660 | 454; Sanger dideoxy sequencing | JGI | PRJNA82765 | SAMN00788628 | 11152 | 10830 | 1 |
| *Aspergillus aff. floccosus IMV 01167* | GCA_001931935.1 | ASM193193v1 | 2562445 | IMV 01167 |  |  |  | 31588614 |  | Scaffold | 04/01/2017 | MSJE01 | 318858 | 482632 | 331 | Illumina HiSeq | Jet Propulsion Laboratory, California Institute of Technology | PRJNA355122 | SAMN06076679 |  |  |  |
| *Aspergillus affinis* | GCF_023601865.1 | ASM2360186v2 | 1070780 | CMG 70 |  |  | Annotation submitted by Universidade de Aveiro | 37565501 |  | Scaffold | 03/10/2022 | JAGXNN02 | 216796 | 216796 | 421 | Illumina HiSeq | Universidade de Aveiro | PRJNA723818 | SAMN18830867 | 11661 | 11661 |  |
| *Aspergillus aflatoxiformans* | GCA_037043995.1 | ASM3704399v1 | 2059437 | LH_A394 |  |  |  | 37779239 |  | Contig | 06/03/2024 | JAWLSX01 | 746223 |  |  | Illumina | Qingdao Agricultural University | PRJNA1025352 | SAMN37865000 |  |  |  |
| *Aspergillus alliaceus* | GCF_009176365.1 | Aspalli1 | 209559 | CBS 536.65 |  |  | Annotation submitted by DOE Joint Genome Institute | 40168528 |  | Scaffold | 18/10/2019 | SWAS01 | 358234 | 499171 | 331 | Illumina | DOE Joint Genome Institute | PRJNA334014 | SAMN05446797 | 13336 | 13098 | 1 |
| *Aspergillus amoenus* | GCA_023624915.1 | ASM2362491v1 | 1220191 | S2904 |  |  |  | 33040702 |  | Scaffold | 01/06/2022 | JAMABK01 | 669357 | 950527 | 147 | Illumina HiSeq | Dalian Medical University | PRJNA833221 | SAMN27963363 |  |  |  |
| *Aspergillus amstelodami* | GCA_027569315.1 | ASM2756931v1 | 5054 | NRRL 89 |  |  |  | 25735522 |  | Contig | 04/01/2023 | JAPDEV01 | 182041 |  |  | Illumina NovaSeq | Northwestern University | PRJNA852164 | SAMN31355053 |  |  |  |
| *Aspergillus arachidicola* | GCA_009193545.1 | Aspara19utr | 656916 | CBS 117612 |  |  | Annotation submitted by DOE Joint Genome Institute | 39764385 |  | Scaffold | 18/10/2019 | STFM01 | 291223 | 349923 | 451 | Illumina | DOE Joint Genome Institute | PRJNA333896 | SAMN05446840 | 14144 | 13894 | 7 |
| *Aspergillus asperescens* | GCA_027569075.1 | ASM2756907v1 | 176163 | NRRL 4770 |  |  |  | 28016043 |  | Contig | 04/01/2023 | JAPDEH01 | 205464 |  |  | Illumina NovaSeq | Northwestern University | PRJNA852164 | SAMN31355035 |  |  |  |
| *Aspergillus austwickii* | GCA_037043805.1 | ASM3704380v1 | 2059439 | LH_A515 |  |  |  | 37479617 |  | Contig | 06/03/2024 | JAWLSO01 | 585932 |  |  | Illumina | Qingdao Agricultural University | PRJNA1025352 | SAMN37865009 |  |  |  |
| *Aspergillus avenaceus* | GCA_009193465.1 | Aspave1 | 36643 | IBT 18842 |  |  | Annotation submitted by DOE Joint Genome Institute | 33765472 |  | Scaffold | 18/10/2019 | STFI01 | 65043 | 79000 | 1528 | Illumina | DOE Joint Genome Institute | PRJNA333897 | SAMN05446841 | 11552 | 11292 | 1 |
| *Aspergillus awamori* | GCA_003850985.1 | Aawm_assembly01 | 105351 | IFM 58123 |  |  | Annotation submitted by Medical Mycology Research Center | 38597812 |  | Contig | 15/10/2018 | BDHI01 | 4298649 |  |  | PacBio RSII | Medical Mycology Research Center | PRJDB4986 | SAMD00056021 | 11669 | 11224 |  |
| *Aspergillus baeticus* | GCA_027569055.1 | ASM2756905v1 | 1194636 | NRRL 62501 |  |  |  | 39786874 |  | Contig | 04/01/2023 | JAPDEI01 | 567911 |  |  | Illumina NovaSeq | Northwestern University | PRJNA852164 | SAMN31355036 |  |  |  |
| *Aspergillus bertholletiae* | GCA_009193595.1 | Aspber1 | 1226010 | IBT 29228 |  |  | Annotation submitted by DOE Joint Genome Institute | 37014000 |  | Scaffold | 18/10/2019 | STFP01 | 127941 | 173958 | 443 | Illumina | DOE Joint Genome Institute | PRJNA333898 | SAMN05446843 | 13190 | 12947 | 1 |
| *Aspergillus biplanus* | GCA_027569045.1 | ASM2756904v1 | 176164 | NRRL 5071 |  |  |  | 42311670 |  | Contig | 04/01/2023 | JAPDEJ01 | 744605 |  |  | Illumina NovaSeq | Northwestern University | PRJNA852164 | SAMN31355037 |  |  |  |
| *Aspergillus bombycis* | GCF_001792695.1 | ASM179269v1 | 109264 | NRRL26010 |  |  | Annotation submitted by USDA-ARS-SRRC | 37474605 |  | Contig | 19/10/2016 | LYCR01 | 240792 |  |  | IonTorrent | USDA-ARS-SRRC | PRJNA320549 | SAMN04942831 | 12263 | 12263 |  |
| *Aspergillus brasiliensis CBS 101740* | GCA_001889945.1 | Aspbr1 | 767769 | CBS 101740 |  |  | Annotation submitted by DOE Joint Genome Institute | 35804080 |  | Scaffold | 08/12/2016 | LJXV01 | 501428 | 2027382 | 103 | Illumina | DOE Joint Genome Institute | PRJNA207691 | SAMN00203938 | 13263 | 12989 | 5 |
| *Aspergillus bridgeri* | GCA_027569665.1 | ASM2756966v1 | 138275 | NRRL 13000 |  |  |  | 40908724 |  | Contig | 04/01/2023 | JAPDKQ01 | 253853 |  |  | Illumina NovaSeq | Northwestern University | PRJNA852164 | SAMN31355038 |  |  |  |
| *Aspergillus brunneoviolaceus CBS 621.78* | GCF_003184695.1 | Aspbru1 | 1450534 | CBS 621.78 |  |  | Annotation submitted by DOE Joint Genome Institute | 37477200 |  | Scaffold | 04/06/2018 | PSTC01 | 253676 | 411666 | 153 | Illumina | DOE Joint Genome Institute | PRJNA235064 | SAMN05660981 | 12391 | 12073 | 2 |
| *Aspergillus brunneus* | GCA_020284055.1 | ASM2028405v1 | 41418 | CBS 112.26 |  |  |  | 25637640 |  | Scaffold | 05/10/2021 | JAILXB01 | 129205 | 183811 | 449 | Illumina | Jet Propulsion Laboratory, California Institute of Technology | PRJNA644637 | SAMN15469713 |  |  |  |
| *Aspergillus caelatus* | GCF_009193585.1 | Aspcae1 | 61420 | CBS 763.97 |  |  | Annotation submitted by DOE Joint Genome Institute | 40016351 |  | Scaffold | 18/10/2019 | STFO01 | 108564 | 115961 | 729 | Illumina | DOE Joint Genome Institute | PRJNA333899 | SAMN05445973 | 14158 | 13914 |  |
| *Aspergillus calidoustus* | GCA_001511075.1 | Acal_Allpaths_LG | 454130 |  |  |  | Annotation submitted by HKI JENA | 41102685 |  | Scaffold | 07/01/2016 | CDMC01 | 981393 | 3207549 | 78 |  | HKI JENA | PRJEB7718 | SAMEA3134252 | 15139 | 15139 |  |
| *Aspergillus campestris IBT 28561* | GCF_002847485.1 | Aspcam1 | 1392248 | IBT 28561 |  |  | Annotation submitted by DOE Joint Genome Institute | 28257496 |  | Contig | 27/12/2017 | MSFM01 | 1703432 |  |  | PacBio | DOE Joint Genome Institute | PRJNA217206 | SAMN02744593 | 9824 | 9655 | 6 |
| *Aspergillus candidus* | GCF_002847045.1 | Aspcand1 | 41067 | CBS 102.13 |  |  | Annotation submitted by DOE Joint Genome Institute | 27317772 |  | Scaffold | 21/12/2017 | PKFS01 | 285163 | 391998 | 268 | Illumina | DOE Joint Genome Institute | PRJNA334018 | SAMN05446799 | 9803 | 9639 | 1 |
| *Aspergillus carbonarius ITEM 5010* | GCA_001990825.1 | Aspca3 | 602072 | ITEM 5010 |  |  | Annotation submitted by DOE Joint Genome Institute | 36149134 |  | Scaffold | 15/02/2017 | AHIG01 | 118896 | 1827541 | 829 | 454; Sanger dideoxy sequencing | DOE Joint Genome Institute | PRJNA34741 | SAMN00727628 | 11735 | 11475 | 4 |
| *Aspergillus carlsbadensis* | GCA_025688815.1 | Aspcar1 | 1810906 | CBS 123894 |  |  | Annotation submitted by DOE Joint Genome Institute | 39460077 |  | Scaffold | 13/10/2022 | SWKC01 | 249132 | 329086 | 254 | Illumina | DOE Joint Genome Institute | PRJNA345930 | SAMN05878104 | 14458 | 14289 | 1 |
| *Aspergillus cavernicola* | GCA_027569065.1 | ASM2756906v1 | 176166 | NRRL 6327 |  |  |  | 36796528 |  | Contig | 04/01/2023 | JAPDEK01 | 274623 |  |  | Illumina NovaSeq | Northwestern University | PRJNA852164 | SAMN31355039 |  |  |  |
| *Aspergillus cejpii* | GCA_004769165.1 | ASM476916v1 | 1884262 | FS110 | China |  |  | 23887422 |  | Contig | 10/04/2019 | SMSW01 | 3505643 |  |  | Illumina HiSeq | Guangdong Institute of Microbiology | PRJNA526604 | SAMN11104596 |  |  |  |
| *Aspergillus chevalieri* | GCF_016861735.1 | AchevalieriM1_assembly01 | 182096 | M1 |  |  | Annotation submitted by Fermentation Microbiology, Education and Research Center for Fermentation Studies, Faculty of Agriculture, Kagoshima University | 29697343 | 8 | Complete Genome | 30/01/2021 |  | 3787471 | 3787471 | 8 | MinION; Illumina NovaSeq 6000 | Fermentation Microbiology, Education and Research Center for Fermentation Studies, Faculty of Agriculture, Kagoshima University | PRJDB10979 | SAMD00269937 | 10518 | 10331 |  |
| *Aspergillus clavatus NRRL 1* | GCF_000002715.2 | ASM271v1 | 344612 | NRRL 1 |  |  | Annotation submitted by J. Craig Venter Institute | 27859441 |  | Scaffold | 14/12/2006 | AAKD03 | 749511 | 2493640 | 143 |  | J. Craig Venter Institute | PRJNA15664 | SAMN02953636 | 9376 | 9118 | 23 |
| *Aspergillus conjunctus* | GCA_027569095.1 | ASM2756909v1 | 41746 | NRRL 5080 |  |  |  | 34028019 |  | Contig | 04/01/2023 | JAPDEL01 | 287669 |  |  | Illumina NovaSeq | Northwestern University | PRJNA852164 | SAMN31355040 |  |  |  |
| *Aspergillus coremiiformis* | GCA_009193565.1 | Aspcor1 | 138285 | CBS 553.77 |  |  | Annotation submitted by DOE Joint Genome Institute | 30132285 |  | Scaffold | 18/10/2019 | STFN01 | 53539 | 58658 | 2728 | Illumina | DOE Joint Genome Institute | PRJNA333900 | SAMN05446844 | 9301 | 9078 | 2 |
| *Aspergillus costaricaensis CBS 115574* | GCF_003184835.1 | Aspcos1 | 1448317 | CBS 115574 |  |  | Annotation submitted by DOE Joint Genome Institute | 36947918 |  | Scaffold | 04/06/2018 | PSTH01 | 426603 | 817474 | 86 | Illumina | DOE Joint Genome Institute | PRJNA235080 | SAMN05660767 | 12238 | 11966 | 5 |
| *Aspergillus costiformis* | GCA_037044115.1 | ASM3704411v1 | 1032661 | LH_A226 |  |  |  | 36665996 |  | Contig | 06/03/2024 | JAWLTF01 | 840845 |  |  | Illumina | Qingdao Agricultural University | PRJNA1025352 | SAMN37864992 |  |  |  |
| *Aspergillus creber* | GCA_021436935.1 | ASM2143693v1 | 1220201 | HOSP050413_5_135 |  |  |  | 34897705 |  | Contig | 11/01/2022 | JAJAEB01 | 710608 |  |  | Illumina NovaSeq | Normandie University | PRJNA768996 | SAMN22074031 |  |  |  |
| *Aspergillus cristatus* | GCA_034509305.1 | ASM3450930v1 | 573508 | 2e |  |  |  | 27754207 |  | Contig | 21/12/2023 | WJBV01 | 3539799 |  |  | PacBio | Tianjin university of commerce | PRJNA587303 | SAMN13186251 |  |  |  |
| *Aspergillus croceus* | GCA_019828945.1 | ASM1982894v1 | 1695224 | JS3-P1 |  |  |  | 22867324 |  | Scaffold | 31/08/2021 | JACVQN01 | 3562 | 3746 | 7378 | Illumina | Jet Propulsion Laboratory, California Institute of Technology | PRJNA644637 | SAMN15793549 |  |  |  |
| *Aspergillus cvjetkovicii* | GCA_027569695.1 | ASM2756969v1 | 1220204 | NRRL 227 |  |  |  | 33734259 |  | Contig | 04/01/2023 | JAPDKS01 | 1010380 |  |  | Illumina NovaSeq | Northwestern University | PRJNA852164 | SAMN31355042 |  |  |  |
| *Aspergillus diversus* | GCA_027569885.1 | ASM2756988v1 | 176167 | NRRL 5074 |  |  |  | 42244320 |  | Contig | 04/01/2023 | JAPDKZ01 | 791273 |  |  | Illumina NovaSeq | Northwestern University | PRJNA852164 | SAMN31427397 |  |  |  |
| *Aspergillus egyptiacus* | GCA_025688805.1 | Aspegy1 | 41743 | CBS 656.73 |  |  | Annotation submitted by DOE Joint Genome Institute | 28335649 |  | Scaffold | 13/10/2022 | SWKD01 | 81542 | 89309 | 682 | Illumina | DOE Joint Genome Institute | PRJNA345891 | SAMN05878059 | 10050 | 9890 |  |
| *Aspergillus elegans* | GCA_016097275.1 | ASM1609727v1 | 93033 | IMI 345568 |  |  |  | 36777498 |  | Contig | 16/12/2020 | JADWDI01 | 4601396 |  |  | PacBio RSII; Illumina | University Complutense of Madrid | PRJNA679179 | SAMN16825489 |  |  |  |
| *Aspergillus ellipticus CBS 707.79* | GCA_003184645.1 | Aspell1 | 1448320 | CBS 707.79 |  |  | Annotation submitted by DOE Joint Genome Institute | 42866077 |  | Scaffold | 04/06/2018 | PSSY01 | 80447 | 158998 | 518 | Illumina | DOE Joint Genome Institute | PRJNA235088 | SAMN05661108 | 13179 | 12882 | 4 |
| *Aspergillus eucalypticola CBS 122712* | GCF_003184535.1 | Aspeuc1 | 1448314 | CBS 122712 |  |  | Annotation submitted by DOE Joint Genome Institute | 34792412 |  | Contig | 04/06/2018 | MSFU01 | 865111 |  |  | PacBio | DOE Joint Genome Institute | PRJNA235076 | SAMN05660444 | 12127 | 11853 | 4 |
| *Aspergillus felis* | GCA_016413765.1 | ASM1641376v1 | 1287682 | FM324 |  |  |  | 33030366 | 8 | Complete Genome | 28/12/2020 |  | 4505747 | 4505747 | 8 | PacBio Sequel | ZHEJIANG UNIVERSITY | PRJNA687106 | SAMN17132962 |  |  |  |
| *Aspergillus fijiensis CBS 313.89* | GCF_003184825.1 | Aspfij1 | 1448319 | CBS 313.89 |  |  | Annotation submitted by DOE Joint Genome Institute | 36513906 |  | Scaffold | 04/06/2018 | PSTG01 | 290429 | 411512 | 149 | Illumina | DOE Joint Genome Institute | PRJNA235087 | SAMN05660285 | 12334 | 12016 | 3 |
| *Aspergillus fischeri NRRL 181* | GCF_000149645.3 | ASM14964v4 | 331117 | NRRL 181 |  |  | Annotation submitted by TIGR | 31377515 |  | Scaffold | 14/12/2006 | AAKE03 | 1240190 | 2929192 | 163 |  | TIGR | PRJNA15672 | SAMN02953637 | 10665 | 10388 | 26 |
| *Aspergillus flavus NRRL3357* | GCF_014117465.1 | ASM1411746v1 | 332952 | NRRL3357 |  |  | Annotation submitted by University of Georgia | 36995582 | 8 | Chromosome | 09/08/2020 |  | 2397812 | 4658713 | 8 | PacBio RSII; Bionano Saphyr | University of Georgia | PRJNA606291 | SAMN14089210 | 12323 | 12009 |  |
| *Aspergillus floridensis* | GCA_012184565.1 | ASM1218456v1 | 1072270 | IHEM 21069 |  |  |  | 35394120 |  | Scaffold | 08/04/2020 | WOUI01 | 72408 | 72599 | 1686 | Illumina HiSeq | Belgian Coordinated Collection of Microorganisms, BCCM | PRJNA574013 | SAMN12838280 |  |  |  |
| *Aspergillus foetidus* | GCA_037044355.1 | ASM3704435v1 | 63131 | LH_A311 |  |  |  | 32951643 |  | Contig | 06/03/2024 | JAWLTP01 | 601137 |  |  | Illumina | Qingdao Agricultural University | PRJNA1025352 | SAMN37864982 |  |  |  |
| *Aspergillus fructus* | GCA_027569115.1 | ASM2756911v1 | 1220205 | NRRL 239 |  |  |  | 33631114 |  | Contig | 04/01/2023 | JAPDEM01 | 1677664 |  |  | Illumina NovaSeq | Northwestern University | PRJNA852164 | SAMN31355043 |  |  |  |
| *Aspergillus fumigatiaffinis* | GCA_012656285.1 | ASM1265628v1 | 340414 | CNM-CM6805 |  |  | Annotation submitted by UNICAMP | 33470809 |  | Scaffold | 22/04/2020 | JAAAPX01 | 154394 | 161182 | 1055 | Illumina NextSeq500 | UNICAMP | PRJNA592352 | SAMN13422803 | 10468 | 10468 |  |
| *Aspergillus fumigatus Af293* | GCF_000002655.1 | ASM265v1 | 330879 | Af293 |  |  | Annotation submitted by J. Craig Venter Institute | 29384958 | 8 | Chromosome | 10/06/2005 | AAHF01 | 2460113 | 3948441 | 8 |  | J. Craig Venter Institute | PRJNA131 | SAMN00115746 | 9915 | 9630 | 57 |
| *Aspergillus funiculosus* | GCA_027569145.1 | ASM2756914v1 | 176168 | NRRL 4744 |  |  |  | 25696535 |  | Contig | 04/01/2023 | JAPDEN01 | 789211 |  |  | Illumina NovaSeq | Northwestern University | PRJNA852164 | SAMN31355044 |  |  |  |
| *Aspergillus glaucus CBS 516.65* | GCF_001890805.1 | Aspgl1 | 1160497 | CBS 516.65 |  |  | Annotation submitted by DOE Joint Genome Institute | 27993362 |  | Scaffold | 08/12/2016 | LSTL01 | 150165 | 1040687 | 82 | Illumina | DOE Joint Genome Institute | PRJNA169684 | SAMN02194406 | 11429 | 11254 | 3 |
| *Aspergillus haitiensis* | GCA_027569155.1 | ASM2756915v1 | 1810914 | NRRL 4568 |  |  |  | 36338520 |  | Contig | 04/01/2023 | JAPDEO01 | 455323 |  |  | Illumina NovaSeq | Northwestern University | PRJNA852164 | SAMN31355045 |  |  |  |
| *Aspergillus hancockii* | GCA_001696595.2 | ASM169659v2 | 1873369 | FRR 3425 |  |  | Annotation submitted by CSIRO | 39691269 |  | Scaffold | 18/09/2020 | MBFL02 | 77984 | 83265 | 1226 | Illumina HiSeq | CSIRO | PRJNA328536 | SAMN05375032 | 12812 | 12583 |  |
| *Aspergillus heteromorphus CBS 117.55* | GCF_003184545.1 | Asphet1 | 1448321 | CBS 117.55 |  |  | Annotation submitted by DOE Joint Genome Institute | 35607567 |  | Contig | 04/06/2018 | MSFL01 | 657941 |  |  | PacBio | DOE Joint Genome Institute | PRJNA235089 | SAMN05660217 | 11089 | 10783 | 1 |
| *Aspergillus hiratsukae* | GCA_014281905.1 | ASM1428190v1 | 1194566 |  |  | CNM-CM5793 | Annotation submitted by UNICAMP | 30071213 |  | Scaffold | 24/08/2020 | JACBAD01 | 99825 | 100545 | 2130 | Illumina NextSeq | UNICAMP | PRJNA633131 | SAMN14933834 | 9685 | 9685 |  |
| *Aspergillus homomorphus CBS 101889* | GCF_003184865.1 | Asphom1 | 1450537 | CBS 101889 |  |  | Annotation submitted by DOE Joint Genome Institute | 34054866 |  | Scaffold | 04/06/2018 | PSTJ01 | 295779 | 535630 | 152 | Illumina | DOE Joint Genome Institute | PRJNA235062 | SAMN05660655 | 11663 | 11361 | 1 |
| *Aspergillus hortae* | GCA_032158085.1 | CIB_AhMCA8_1.0 | 1107321 | MCA8 |  |  |  | 31114527 |  | Scaffold | 28/09/2023 | JASVVY01 | 1070508 | 1301367 | 159 | Illumina | Corporacion para Investigaciones Biologicas | PRJNA975750 | SAMN35344991 |  |  |  |
| *Aspergillus ibericus CBS 121593* | GCF_003184845.1 | Aspibe1 | 1448316 | CBS 121593 |  |  | Annotation submitted by DOE Joint Genome Institute | 33437983 |  | Scaffold | 04/06/2018 | PSTI01 | 283478 | 622403 | 116 | Illumina | DOE Joint Genome Institute | PRJNA235079 | SAMN05660294 | 11989 | 11680 | 5 |
| *Aspergillus incahuasiensis* | GCA_003719405.1 | ASM371940v1 | 2484740 | NRRL 66825 |  |  |  | 31006665 |  | Scaffold | 09/11/2018 | RHLP01 | 41769 | 41769 | 1939 | Illumina MiSeq | National Center For Agricultural Utilization Research | PRJNA498066 | SAMN10280326 |  |  |  |
| *Aspergillus indologenus CBS 114.80* | GCA_003184685.1 | Aspind1 | 1450541 | CBS 114.80 |  |  | Annotation submitted by DOE Joint Genome Institute | 38592838 |  | Scaffold | 04/06/2018 | PSTB01 | 139187 | 235731 | 334 | Illumina | DOE Joint Genome Institute | PRJNA235059 | SAMN05661047 | 12485 | 12161 | 8 |
| *Aspergillus intermedius* | GCA_039880815.1 | ASM3988081v1 | 41412 | E25 |  |  |  | 26777181 |  | Scaffold | 28/05/2024 | JBDODA01 | 219895 | 236062 | 496 | Illumina MiSeq | IRSET | PRJNA802395 | SAMN25554602 |  |  |  |
| *Aspergillus japonicus CBS 114.51* | GCF_003184785.1 | Aspjap1 | 1448312 | CBS 114.51 |  |  | Annotation submitted by DOE Joint Genome Institute | 36099954 |  | Scaffold | 04/06/2018 | PSTF01 | 226011 | 412690 | 163 | Illumina | DOE Joint Genome Institute | PRJNA235073 | SAMN05660717 | 12345 | 12022 | 6 |
| *Aspergillus jensenii* | GCA_021437015.1 | ASM2143701v1 | 1220206 | C4_18042019 |  |  |  | 35429369 |  | Contig | 11/01/2022 | JAJAED01 | 866901 |  |  | Illumina NovaSeq | Normandie University | PRJNA769000 | SAMN22074134 |  |  |  |
| *Aspergillus kassunensis* | GCA_027569205.1 | ASM2756920v1 | 469275 | NRRL 3752 |  |  |  | 34487739 |  | Contig | 04/01/2023 | JAPDEQ01 | 506921 |  |  | Illumina NovaSeq | Northwestern University | PRJNA852164 | SAMN31355047 |  |  |  |
| *Aspergillus latus* | GCA_013306195.1 | ASM1330619v1 | 41734 | MM151978 |  |  |  | 63570296 |  | Scaffold | 09/06/2020 | VCRL01 | 172899 | 395084 | 2405 | Illumina | Vanderbilt University | PRJNA542678 | SAMN11615378 |  |  |  |
| *Aspergillus lentulus* | GCF_010724455.1 | ASM1072445v1 | 293939 | IFM 58399 |  |  | Annotation submitted by Medical Mycology Research Center | 30229310 |  | Scaffold | 04/02/2020 | BLKH01 | 247240 | 315630 | 760 | HiSeq 1500 | Medical Mycology Research Center | PRJDB8156 | SAMD00166943 | 10506 | 10293 |  |
| *Aspergillus leporis* | GCA_009176345.1 | Asplep1 | 41062 | CBS 151.66 |  |  | Annotation submitted by DOE Joint Genome Institute | 39425861 |  | Scaffold | 18/10/2019 | SWBU01 | 134759 | 157399 | 615 | Illumina | DOE Joint Genome Institute | PRJNA333901 | SAMN05446746 | 12986 | 12743 |  |
| *Aspergillus luchuensis* | GCF_016861625.1 | AkawachiiIFO4308_assembly01 | 1069201 | IFO 4308 |  |  | Annotation submitted by Fermentation Microbiology, Education and Research Center for Fermentation Studies, Faculty of Agriculture, Kagoshima University | 37256495 | 8 | Complete Genome | 30/01/2021 |  | 4830482 | 4830482 | 8 | MinION; Illumina NovaSeq 6000 | Fermentation Microbiology, Education and Research Center for Fermentation Studies, Faculty of Agriculture, Kagoshima University | PRJDB10968 | SAMD00269936 | 12941 | 12662 |  |
| *Aspergillus melleus* | GCF_016097325.1 | ASM1609732v1 | 138277 | CBS 546.65 |  |  | Annotation submitted by University Complutense of Madrid | 38850827 |  | Contig | 16/12/2020 | JADPPX01 | 5594283 |  |  | PacBio RSII; Illumina | University Complutense of Madrid | PRJNA679179 | SAMN16825490 | 13060 | 12848 | 1 |
| *Aspergillus micronesiensis* | GCA_039763685.1 | ASM3976368v1 | 1507543 | H39 |  |  |  | 32328115 |  | Contig | 22/05/2024 | JASHHO01 | 594468 |  |  | PacBio | Biology Institute, Qilu University of Technology (Shandong Academy of Sciences) | PRJNA939002 | SAMN33453546 |  |  |  |
| *Aspergillus minisclerotigenes* | GCA_028505775.1 | ASM2850577v1 | 656917 | MRI390 |  |  |  | 38025472 | 8 | Chromosome | 21/02/2023 | JAHXGP01 | 360475 | 3823310 | 28 | PacBio Sequel; Illumina MiniSeq | Max Rubner-Institut | PRJNA742918 | SAMN19989186 |  |  |  |
| *Aspergillus montevidensis* | GCA_020826735.1 | ASM2082673v1 | 1173306 | ZYD4 |  |  |  | 26622312 |  | Contig | 10/11/2021 | JAJFZZ01 | 3879884 |  |  | Illumina NovaSeq; PacBio Sequel | Shaanxi university of science and technology | PRJNA775823 | SAMN22627947 |  |  |  |
| *Aspergillus mulundensis* | GCF_003369625.1 | ASM336962v1 | 1810919 | DSM 5745 |  |  | Annotation submitted by The University of Texas Health Science Center at Houston | 45341896 |  | Scaffold | 10/08/2018 | PVWQ01 | 218918 | 2829575 | 160 | Illumina HiSeq | The University of Texas Health Science Center at Houston | PRJNA389331 | SAMN07195632 | 11603 | 11603 |  |
| *Aspergillus nanangensis* | GCA_015844325.1 | ASM1584432v1 | 2582783 | MST-FP2251 |  |  | Annotation submitted by The University of Western Australia | 36245875 |  | Scaffold | 08/12/2020 | VCAU01 | 155554 | 231700 | 438 | Illumina NextSeq | The University of Western Australia | PRJNA543384 | SAMN11665376 | 13097 | 12935 | 1 |
| *Aspergillus neoniger CBS 115656* | GCF_003184625.1 | Aspneo1 | 1448310 | CBS 115656 |  |  | Annotation submitted by DOE Joint Genome Institute | 35416444 |  | Scaffold | 04/06/2018 | MSFP01 | 343923 | 707186 | 169 | Illumina | DOE Joint Genome Institute | PRJNA235072 | SAMN05660318 | 12223 | 11939 | 4 |
| *Aspergillus neotritici* | GCA_023619815.1 | ASM2361981v1 | 2932460 | S2209 |  |  |  | 28548381 |  | Scaffold | 01/06/2022 | JALZYL01 | 347985 | 574990 | 480 | Illumina HiSeq | Dalian Medical University | PRJNA833221 | SAMN27963249 |  |  |  |
| *Aspergillus nidulans FGSC A4* | GCF_000011425.1 | ASM1142v1 | 227321 | FGSC A4 |  |  | Annotation submitted by Eurofungbase (Eurofung) | 29828291 | 8 | Chromosome | 24/09/2009 |  | 679860 | 3704807 | 8 |  | Eurofungbase (Eurofung) | PRJEA40559 | SAMEA2272224 | 10518 | 10455 | 63 |
| *Aspergillus niger* | GCF_000002855.4 | ASM285v2 | 5061 |  |  |  | Annotation submitted by DSM, The Netherlands | 33975768 | 8 | Scaffold | 11/04/2007 |  | 114002 | 2525243 | 19 |  | DSM, The Netherlands | PRJNA19275 | SAMEA3283178 | 14404 | 14058 | 17 |
| *Aspergillus nomiae NRRL 13137* | GCF_001204775.2 | ASM120477v2 | 1509407 | NRRL 13137 |  |  | Annotation submitted by USDA-ARS-SRRC | 36137300 |  | Contig | 04/08/2015 | JNOM01 | 66657 |  |  | IonTorrent | USDA-ARS-SRRC | PRJNA246595 | SAMN02768702 | 11904 | 11904 |  |
| *Aspergillus novofumigatus IBT 16806* | GCF_002847465.1 | Aspnov1 | 1392255 | IBT 16806 |  |  | Annotation submitted by DOE Joint Genome Institute | 32440129 |  | Contig | 27/12/2017 | MSZS01 | 3768347 |  |  | PacBio | DOE Joint Genome Institute | PRJNA217212 | SAMN02744592 | 11620 | 11426 | 12 |
| *Aspergillus novoparasiticus* | GCA_009176405.1 | Aspnovo1 | 986946 | CBS 126849 |  |  | Annotation submitted by DOE Joint Genome Institute | 40857493 |  | Scaffold | 18/10/2019 | SWDA01 | 241198 | 276844 | 870 | Illumina | DOE Joint Genome Institute | PRJNA333905 | SAMN05446749 | 14431 | 14182 | 5 |
| *Aspergillus ochraceoroseus IBT 24754* | GCF_002846915.1 | Aspergillus ochraceoroseus IBT 24754 v1.1 | 1392256 | IBT 24754 |  |  | Annotation submitted by DOE Joint Genome Institute | 27718211 |  | Contig | 16/04/2018 | MSFN02 | 2489623 |  |  | PacBio | DOE Joint Genome Institute | PRJNA217213 | SAMN02744596 | 8974 | 8825 | 3 |
| *Aspergillus ochraceus* | GCA_004849945.1 | AoFc | 40380 | fc-1 |  |  |  | 37017470 |  | Scaffold | 24/04/2019 | SSOS01 | 1342807 | 3843998 | 21 | Illumina | Institute of Agro-Products Processing Science and Technology, Chinese Academy of Agricultural Sciences (CAAS) | PRJNA264608 | SAMN03140103 |  |  |  |
| *Aspergillus oerlinghausenensis* | GCA_014250555.1 | ASM1425055v1 | 1774284 | CBS 139183 |  |  |  | 32321563 |  | Scaffold | 19/08/2020 | JAAKEQ01 | 395561 | 508616 | 1460 | Illumina HiSeq | Vanderbilt University | PRJNA577646 | SAMN13030901 |  |  |  |
| *Aspergillus olivimuriae* | GCA_003719415.1 | ASM371941v1 | 2184023 | NRRL 66783 |  |  |  | 30085639 |  | Scaffold | 09/11/2018 | RHLO01 | 27036 | 27163 | 2603 | Illumina MiSeq | National Center For Agricultural Utilization Research | PRJNA498048 | SAMN10279383 |  |  |  |
| *Aspergillus oryzae RIB40* | GCF_000184455.2 | ASM18445v3 | 510516 | RIB40 |  |  | Annotation submitted by National Institute of Technology and Evaluation (NITE), Japan | 37882812 | 8 | Chromosome | 24/06/2011 |  | 2324132 | 4887096 | 11 |  | National Institute of Technology and Evaluation (NITE), Japan | PRJNA20809 | SAMD00067075 | 12347 | 12074 | 3 |
| *Aspergillus ostianus* | GCA_025592935.1 | ASM2559293v1 | 138279 | IFST Aos1 |  |  |  | 35685113 |  | Scaffold | 05/10/2022 | JANFQY01 | 88477 | 94331 | 1290 | Illumina HiSeq | Institute of Food Science and Technology, CAAS | PRJNA765789 | SAMN29827629 |  |  |  |
| *Aspergillus panamensis* | GCA_027569225.1 | ASM2756922v1 | 41747 | NRRL 1786 |  |  |  | 31672359 |  | Contig | 04/01/2023 | JAPDER01 | 377292 |  |  | Illumina NovaSeq | Northwestern University | PRJNA852164 | SAMN31355049 |  |  |  |
| *Aspergillus parasiticus* | GCA_028505765.1 | ASM2850576v1 | 5067 |  |  | MRI410 |  | 38682576 | 8 | Scaffold | 21/02/2023 | JAMRJN01 | 2119486 | 2119486 | 60 | Illumina MiSeq; Oxford Nanopore MinION | Max Rubner-Institut | PRJNA835319 | SAMN28100224 |  |  |  |
| *Aspergillus persii* | GCA_002215965.1 | ASM221596v1 | 306094 | NIBRFGC000004109 |  |  |  | 38414373 |  | Scaffold | 07/07/2017 | NGZO01 | 279982 | 4596147 | 12 | Illumina HiSeq; PacBio | National Institute of Biological Resources | PRJNA368784 | SAMN06272962 |  |  |  |
| *Aspergillus phoenicis ATCC 13157* | GCA_003344505.1 | Aspph1 | 1353007 | ATCC 13157 |  |  | Annotation submitted by DOE Joint Genome Institute | 35753538 |  | Scaffold | 27/07/2018 | QQUR01 | 695580 | 1344691 | 55 | Illumina | DOE Joint Genome Institute | PRJNA209548 | SAMN02744683 | 12265 | 11972 | 10 |
| *Aspergillus piperis CBS 112811* | GCF_003184755.1 | Asppip1 | 1448313 | CBS 112811 |  |  | Annotation submitted by DOE Joint Genome Institute | 35280331 |  | Scaffold | 04/06/2018 | PSTD01 | 469711 | 1121342 | 47 | Illumina | DOE Joint Genome Institute | PRJNA235074 | SAMN05660207 | 12360 | 12071 | 5 |
| *Aspergillus pragensis* | GCA_037044195.1 | ASM3704419v1 | 1458898 | LH_A260 |  |  |  | 27565195 |  | Contig | 06/03/2024 | JAWLTI01 | 350522 |  |  | Illumina | Qingdao Agricultural University | PRJNA1025352 | SAMN37864989 |  |  |  |
| *Aspergillus proliferans* | GCA_039880865.1 | ASM3988086v1 | 41414 | E30 |  |  |  | 28912890 |  | Scaffold | 28/05/2024 | JBDODB01 | 78218 | 79997 | 918 | Illumina MiSeq | IRSET | PRJNA802395 | SAMN25554603 |  |  |  |
| *Aspergillus protuberus* | GCA_021436995.1 | ASM2143699v1 | 469277 | HOSP050413_4_129 |  |  |  | 34621709 |  | Scaffold | 11/01/2022 | JAJAEC01 | 678257 | 1006248 | 200 | Illumina NovaSeq | Normandie University | PRJNA768998 | SAMN22074035 |  |  |  |
| *Aspergillus pseudocaelatus* | GCA_009193665.1 | Asppsec1 | 1825620 | CBS 117616 |  |  | Annotation submitted by DOE Joint Genome Institute | 39651556 |  | Scaffold | 18/10/2019 | STFS01 | 151791 | 210129 | 466 | Illumina | DOE Joint Genome Institute | PRJNA333908 | SAMN05445982 | 14138 | 13895 |  |
| *Aspergillus pseudoglaucus* | GCA_039880875.1 | ASM3988087v1 | 1405805 | E42 |  |  |  | 26537594 |  | Scaffold | 28/05/2024 | JBDODC01 | 530501 | 599006 | 165 | Oxford Nanopore MinION; Illumina MiSeq | IRSET | PRJNA802395 | SAMN25554604 |  |  |  |
| *Aspergillus pseudonomiae* | GCF_009193645.1 | Asppsen1 | 1506151 | CBS 119388 |  |  | Annotation submitted by DOE Joint Genome Institute | 37780514 |  | Scaffold | 18/10/2019 | STFR01 | 188686 | 271263 | 374 | Illumina | DOE Joint Genome Institute | PRJNA333909 | SAMN05446751 | 13621 | 13384 | 4 |
| *Aspergillus pseudotamarii* | GCF_009193445.1 | Asppset1 | 132259 | CBS 117625 |  |  | Annotation submitted by DOE Joint Genome Institute | 38243929 |  | Scaffold | 18/10/2019 | STFH01 | 260127 | 410003 | 249 | Illumina | DOE Joint Genome Institute | PRJNA333910 | SAMN05446752 | 13663 | 13428 | 1 |
| *Aspergillus pseudoterreus* | GCA_002927005.1 | ASM292700v1 | 1565506 | ATCC 32359 |  |  |  | 29514308 |  | Scaffold | 14/02/2018 | PIJX01 | 554745 | 678262 | 270 | Illumina MiSeq | Pacific Northwest National Lab | PRJNA420104 | SAMN08106046 |  |  |  |
| *Aspergillus pseudoviridinutans* | GCF_018340605.1 | Asppvi_assembly01 | 1517512 | IFM 55266 |  |  | Annotation submitted by Medical Mycology Research Center | 33330675 |  | Scaffold | 12/03/2021 | BHVY01 | 1135465 | 4753445 | 24 | Illumina HiSeq X; Illumina HiSeq 2500 | Medical Mycology Research Center | PRJDB7449 | SAMD00140677 | 11495 | 11280 |  |
| *Aspergillus puulaauensis* | GCF_016861865.1 | ApuulaauensisMK2_assembly01 | 1220207 | MK2 |  |  | Annotation submitted by Fermentation Microbiology, Education and Research Center for Fermentation Studies, Faculty of Agriculture, Kagoshima University | 34318862 | 8 | Complete Genome | 30/01/2021 |  | 4388637 | 4388637 | 8 | MinION; Illumina NovaSeq 6000 | Fermentation Microbiology, Education and Research Center for Fermentation Studies, Faculty of Agriculture, Kagoshima University | PRJDB10980 | SAMD00269938 | 13749 | 13606 |  |
| *Aspergillus quadricinctus* | GCA_022814375.1 | ASM2281437v1 | 41053 | FKII-L3-BK-DRAB1 |  |  |  | 34430349 |  | Scaffold | 04/04/2022 | JAKLNT01 | 293441 | 359761 | 649 | Illumina NovaSeq | Jet Propulsion Laboratory, California Institute of Technology | PRJNA800051 | SAMN25226826 |  |  |  |
| *Aspergillus quadrilineatus* | GCA_013305525.1 | ASM1330552v1 | 41735 | NRRL 201 |  | USDA |  | 32173992 |  | Scaffold | 09/06/2020 | JAAXYA01 | 266750 | 293960 | 368 | Illumina HiSeq | Vanderbilt University | PRJNA623402 | SAMN14546090 |  |  |  |
| *Aspergillus rambellii* | GCA_000986645.1 | ASM98664v1 | 308745 | SRRC1468 |  |  | Annotation submitted by USDA-ARS-SRRC | 26435630 |  | Contig | 30/04/2015 | JZBS01 | 37599 |  |  | IonTorrent | USDA-ARS-SRRC | PRJNA275129 | SAMN03339590 | 7761 | 7761 |  |
| *Aspergillus ruber CBS 135680* | GCF_000600275.1 | Eurhe1 | 1388766 | CBS 135680 |  |  | Annotation submitted by DOE Joint Genome Institute | 26209327 |  | Scaffold | 27/03/2014 | AWRT01 | 155194 | 623674 | 110 | Illumina | DOE Joint Genome Institute | PRJNA215335 | SAMN02744096 | 10239 | 10064 | 2 |
| *Aspergillus saccharolyticus JOP 1030-1* | GCF_003184585.1 | Aspsac1 | 1450539 | JOP 1030-1 |  |  | Annotation submitted by DOE Joint Genome Institute | 31096189 |  | Scaffold | 04/06/2018 | MSFQ01 | 268948 | 591538 | 120 | Illumina | DOE Joint Genome Institute | PRJNA235058 | SAMN05660449 | 10381 | 10064 | 3 |
| *Aspergillus sclerotialis* | GCA_003589665.1 | phiScl 1.0 | 2070753 | CBS 366.77 |  |  | Annotation submitted by University of Natural Resources and Life Sciences, Vienna | 27975818 |  | Contig | 21/09/2018 | MVGC01 | 40988 |  |  | IonTorrent | University of Natural Resources and Life Sciences, Vienna | PRJNA369220 | SAMN06279797 | 11307 | 11307 |  |
| *Aspergillus sclerotiicarbonarius CBS 121057* | GCA_003184635.1 | Aspscle1 | 1448318 | CBS 121057 |  |  | Annotation submitted by DOE Joint Genome Institute | 37621264 |  | Scaffold | 04/06/2018 | PSSZ01 | 225934 | 362770 | 166 | Illumina | DOE Joint Genome Institute | PRJNA235081 | SAMN05660331 | 12893 | 12569 | 4 |
| *Aspergillus sclerotioniger CBS 115572* | GCF_003184525.1 | Aspscl1 | 1450535 | CBS 115572 |  |  | Annotation submitted by DOE Joint Genome Institute | 36720365 |  | Contig | 04/06/2018 | MSFK01 | 930588 |  |  | PacBio | DOE Joint Genome Institute | PRJNA235065 | SAMN05661070 | 12650 | 12338 | 2 |
| *Aspergillus sclerotiorum* | GCA_025592505.1 | ASM2559250v1 | 138282 | IFST Ascl1 |  |  |  | 35883475 |  | Scaffold | 05/10/2022 | JANFQX01 | 121237 | 134171 | 1261 | Illumina HiSeq | Institute of Food Science and Technology, CAAS | PRJNA765789 | SAMN29827630 |  |  |  |
| *Aspergillus sergii* | GCA_009193525.1 | Aspser1 | 1034303 | CBS 130017 |  |  | Annotation submitted by DOE Joint Genome Institute | 38325693 |  | Scaffold | 18/10/2019 | STFL01 | 306428 | 382883 | 262 | Illumina | DOE Joint Genome Institute | PRJNA333911 | SAMN05443096 | 13964 | 13713 | 7 |
| *Aspergillus sojae* | GCA_008274985.1 | ASM827498v1 | 41058 | SMF134 |  |  |  | 40106795 | 8 | Complete Genome | 05/09/2019 |  | 5060128 | 5060128 | 8 | PacBio RS | Kookmin University | PRJNA517748 | SAMN10844115 |  |  |  |
| *Aspergillus sp. 'subgen. Cremei'* | GCA_019976455.1 | ASM1997645v1 | 2894562 | IBT 35662 |  |  |  | 32156665 |  | Contig | 14/09/2021 | JAFBMR01 | 4532342 |  |  | Oxford Nanopore MinION | Aalborg university | PRJNA698796 | SAMN17736651 |  |  |  |
| *Aspergillus sp. 2663* | GCA_016880755.1 | ASM1688075v1 | 2681688 | 2663 |  |  |  | 34304860 |  | Scaffold | 12/02/2021 | WOYA01 | 879760 | 999076 | 124 | Illumina MiSeq; PacBio Sequel | Seqomics Ltd. | PRJNA592895 | SAMN13442836 |  |  |  |
| *Aspergillus sp. 2NF922* | GCA_023625275.1 | ASM2362527v1 | 2940428 | 2NF922 |  |  |  | 33287624 |  | Scaffold | 01/06/2022 | JAMAAT01 | 823382 | 1224473 | 363 | Illumina HiSeq | Dalian Medical University | PRJNA833221 | SAMN27963345 |  |  |  |
| *Aspergillus sp. 2NF923* | GCA_023625115.1 | ASM2362511v1 | 2940435 | 2NF923 |  |  |  | 33243559 |  | Scaffold | 01/06/2022 | JAMABA01 | 783089 | 1541680 | 351 | Illumina HiSeq | Dalian Medical University | PRJNA833221 | SAMN27963353 |  |  |  |
| *Aspergillus sp. 2NF925* | GCA_023625195.1 | ASM2362519v1 | 2940433 | 2NF925 |  |  |  | 33298868 |  | Scaffold | 01/06/2022 | JAMAAY01 | 789314 | 1453397 | 359 | Illumina HiSeq | Dalian Medical University | PRJNA833221 | SAMN27963351 |  |  |  |
| *Aspergillus sp. A31* | GCA_016162245.1 | ASM1616224v1 | 2609453 | A31 |  |  |  | 28603976 |  | Contig | 21/12/2020 | JACBKQ01 | 417712 |  |  | Illumina HiSeq | UFMT | PRJNA564986 | SAMN15373983 |  |  |  |
| *Aspergillus sp. ADI1* | GCA_020615375.1 | CIT_ADI_1 | 2862157 | ADI1 |  |  |  | 38902458 |  | Contig | 26/10/2021 | JAHXQV01 | 773491 |  |  | Illumina; PacBio | California Institute of Technology | PRJNA748778 | SAMN20344550 |  |  |  |
| *Aspergillus sp. AS01* | GCA_036986335.1 | ASM3698633v1 | 3095026 | AS01 |  |  |  | 37733012 |  | Contig | 05/01/2024 | BTWD01 | 339095 |  |  | Illumina MiSeq | Shubun University | PRJDB16793 | SAMD00649585 |  |  |  |
| *Aspergillus sp. ATCC 12892* | GCA_002894705.1 | ASM289470v1 | 2715684 | ATCC 12892 |  |  |  | 41166126 |  | Scaffold | 22/01/2018 | NVQI01 | 469295 | 665651 | 715 | Illumina MiSeq | Pacific Northwest National Lab | PRJNA407216 | SAMN07638907 |  |  |  |
| *Aspergillus sp. B102-05B* | GCA_037042565.1 | ASM3704256v1 | 3092684 | B102-05B |  |  |  | 34739543 |  | Scaffold | 06/03/2024 | JAXQKO01 | 722785 | 1055112 | 377 | Illumina HiSeq | Dalian Medical University | PRJNA833221 | SAMN38094937 |  |  |  |
| *Aspergillus sp. B3601* | GCA_023625155.1 | ASM2362515v1 | 2940430 | B3601 |  |  |  | 35630518 |  | Scaffold | 01/06/2022 | JAMAAV01 | 614786 | 1064522 | 529 | Illumina HiSeq | Dalian Medical University | PRJNA833221 | SAMN27963348 |  |  |  |
| *Aspergillus sp. B3605* | GCA_023625035.1 | ASM2362503v1 | 2940439 | B3605 |  |  |  | 34721428 |  | Scaffold | 01/06/2022 | JAMABE01 | 748844 | 1406545 | 450 | Illumina HiSeq | Dalian Medical University | PRJNA833221 | SAMN27963357 |  |  |  |
| *Aspergillus sp. DX121-18F* | GCA_037042555.1 | ASM3704255v1 | 3092690 | DX121-18F |  |  |  | 34052985 |  | Contig | 06/03/2024 | JAXQKQ01 | 14357 |  |  | Illumina HiSeq | Dalian Medical University | PRJNA833221 | SAMN38094948 |  |  |  |
| *Aspergillus sp. DX7217* | GCA_037042595.1 | ASM3704259v1 | 3092694 | DX7217 |  |  |  | 34982820 |  | Scaffold | 06/03/2024 | JAXQKR01 | 196172 | 196172 | 609 | Illumina HiSeq | Dalian Medical University | PRJNA833221 | SAMN38094958 |  |  |  |
| *Aspergillus sp. F3601* | GCA_023619735.1 | ASM2361973v1 | 2940397 | F3601 |  |  |  | 33620165 |  | Scaffold | 01/06/2022 | JALZYP01 | 1035468 | 1997738 | 151 | Illumina HiSeq | Dalian Medical University | PRJNA833221 | SAMN27963254 |  |  |  |
| *Aspergillus sp. F3603* | GCA_023627655.1 | ASM2362765v1 | 2940329 | S1902Y |  |  |  | 33644265 |  | Scaffold | 01/06/2022 | JAMAHS01 | 325389 | 379174 | 249 | Illumina HiSeq | Dalian Medical University | PRJNA833221 | SAMN27963367 |  |  |  |
| *Aspergillus sp. F3604* | GCA_023619485.1 | ASM2361948v1 | 2940409 | F3604 |  |  |  | 34895846 |  | Scaffold | 01/06/2022 | JALZZB01 | 709258 | 954997 | 220 | Illumina HiSeq | Dalian Medical University | PRJNA833221 | SAMN27963266 |  |  |  |
| *Aspergillus sp. F3605* | GCA_023619445.1 | ASM2361944v1 | 2940411 | F3605 |  |  |  | 34734933 |  | Scaffold | 01/06/2022 | JALZZD01 | 939363 | 1257850 | 347 | Illumina HiSeq | Dalian Medical University | PRJNA833221 | SAMN27963268 |  |  |  |
| *Aspergillus sp. GBTC2 WXH* | GCA_026119255.1 | ASM2611925v1 | 2973950 | GBTC2 WXH |  |  |  | 37875941 |  | Contig | 10/11/2022 | JANZLR01 | 4075796 |  |  | PacBio Sequel | Central South University | PRJNA870667 | SAMN30399843 |  |  |  |
| *Aspergillus sp. GbtcF1* | GCA_019176365.1 | ASM1917636v1 | 2824782 | GbtcF1 |  |  |  | 28831586 |  | Contig | 08/07/2021 | JAGXKD01 | 3752118 |  |  | BGISEQ-500; PacBio Sequel | Central South University | PRJNA720764 | SAMN18679182 |  |  |  |
| *Aspergillus sp. GbtcF2* | GCA_019176375.1 | ASM1917637v1 | 2824783 | GbtcF2 |  |  |  | 37875941 |  | Contig | 08/07/2021 | JAGXKE01 | 4075796 |  |  | BGISEQ-500; PacBio Sequel | Central South University | PRJNA720764 | SAMN18679183 |  |  |  |
| *Aspergillus sp. HF37* | GCA_003698115.1 | ASM369811v1 | 1960876 | HF37 |  |  | Annotation submitted by University of Natural Resources and Life Sciences, Vienna | 21930979 |  | Contig | 30/10/2018 | RAQS01 | 38782 |  |  | IonTorrent | University of Natural Resources and Life Sciences, Vienna | PRJNA374997 | SAMN06341106 | 8895 | 8895 |  |
| *Aspergillus sp. JS3-R5* | GCA_019775315.1 | ASM1977531v1 | 2756248 | JS3-R5 |  |  |  | 25520748 |  | Scaffold | 26/08/2021 | JACWGA01 | 1284660 | 1625449 | 69 | Illumina | Jet Propulsion Laboratory, California Institute of Technology | PRJNA644637 | SAMN15543690 |  |  |  |
| *Aspergillus sp. M3605* | GCA_023619345.1 | ASM2361934v1 | 2940416 | M3605 |  |  |  | 35891085 |  | Scaffold | 01/06/2022 | JALZZI01 | 443640 | 539287 | 354 | Illumina HiSeq | Dalian Medical University | PRJNA833221 | SAMN27963273 |  |  |  |
| *Aspergillus sp. M3606* | GCA_023619275.1 | ASM2361927v1 | 2940420 | M3606 |  |  |  | 35917545 |  | Scaffold | 01/06/2022 | JALZZM01 | 458149 | 535809 | 345 | Illumina HiSeq | Dalian Medical University | PRJNA833221 | SAMN27963278 |  |  |  |
| *Aspergillus sp. MA 6037* | GCA_003138035.1 | ASM313803v1 | 2153246 | MA 6037 |  |  |  | 32952336 |  | Contig | 18/05/2018 | QAGH01 | 26272 |  |  | IonTorrent | University of Natural Resources and Life Sciences, Vienna | PRJNA432315 | SAMN08819844 |  |  |  |
| *Aspergillus sp. MA 6041* | GCA_025768905.1 | ASM2576890v1 | 2153248 | Y3614 |  |  |  | 33343035 |  | Scaffold | 20/10/2022 | JAMFNV01 | 890588 | 1820991 | 277 | Illumina HiSeq | Dalian Medical University | PRJNA833221 | SAMN27963347 |  |  |  |
| *Aspergillus sp. MEXU 27854* | GCA_019721355.1 | Asp_MEXU27854_1.0 | 2801328 | MEXU 27854 |  |  |  | 30756112 |  | Contig | 23/08/2021 | JAGMTT01 | 3946678 |  |  | PacBio Sequel II | UNIVERSIDAD NACIONAL AUTONOMA DE MEXICO | PRJNA689854 | SAMN17220881 |  |  |  |
| *Aspergillus sp. P7301S-1* | GCA_037042525.1 | ASM3704252v1 | 3092718 | P7301S-1 |  |  |  | 25457646 |  | Scaffold | 06/03/2024 | JAXQKN01 | 172932 | 172932 | 402 | Illumina HiSeq | Dalian Medical University | PRJNA833221 | SAMN38095022 |  |  |  |
| *Aspergillus sp. PA2101* | GCA_023619705.1 | ASM2361970v1 | 2940399 | PA2101 |  |  |  | 34494428 |  | Scaffold | 01/06/2022 | JALZYR01 | 792993 | 1391688 | 279 | Illumina HiSeq | Dalian Medical University | PRJNA833221 | SAMN27963256 |  |  |  |
| *Aspergillus sp. PA3604* | GCA_023624935.1 | ASM2362493v1 | 2940440 | PA3604 |  |  |  | 35261291 |  | Scaffold | 01/06/2022 | JAMABJ01 | 643727 | 835375 | 564 | Illumina HiSeq | Dalian Medical University | PRJNA833221 | SAMN27963362 |  |  |  |
| *Aspergillus sp. PA40-03* | GCA_023618985.1 | ASM2361898v1 | 2940422 | PA40-03 |  |  |  | 33564663 |  | Scaffold | 01/06/2022 | JALZZO01 | 834636 | 1353186 | 244 | Illumina HiSeq | Dalian Medical University | PRJNA833221 | SAMN27963280 |  |  |  |
| *Aspergillus sp. PB2407* | GCA_023619755.1 | ASM2361975v1 | 2940396 | PB2407 |  |  |  | 33528687 |  | Scaffold | 01/06/2022 | JALZYO01 | 1629627 | 2240214 | 52 | Illumina HiSeq | Dalian Medical University | PRJNA833221 | SAMN27963253 |  |  |  |
| *Aspergillus sp. PB4102* | GCA_023619575.1 | ASM2361957v1 | 2940404 | PB4102 |  |  |  | 34477307 |  | Scaffold | 01/06/2022 | JALZYW01 | 774752 | 1489079 | 218 | Illumina HiSeq | Dalian Medical University | PRJNA833221 | SAMN27963261 |  |  |  |
| *Aspergillus sp. PB4105* | GCA_023618955.1 | ASM2361895v1 | 2940424 | PB4105 |  |  |  | 34164506 |  | Scaffold | 01/06/2022 | JALZZQ01 | 827402 | 1295238 | 388 | Illumina HiSeq | Dalian Medical University | PRJNA833221 | SAMN27963282 |  |  |  |
| *Aspergillus sp. PB4106* | GCA_023619455.1 | ASM2361945v1 | 2940410 | PB4106 |  |  |  | 34486824 |  | Scaffold | 01/06/2022 | JALZZC01 | 887985 | 2430512 | 189 | Illumina HiSeq | Dalian Medical University | PRJNA833221 | SAMN27963267 |  |  |  |
| *Aspergillus sp. PG119-06* | GCA_037042605.1 | ASM3704260v1 | 3092727 | PG119-06 |  |  |  | 25255761 |  | Scaffold | 06/03/2024 | JAXQKT01 | 232214 | 232214 | 251 | Illumina HiSeq | Dalian Medical University | PRJNA833221 | SAMN38095039 |  |  |  |
| *Aspergillus sp. PG3603* | GCA_023619615.1 | ASM2361961v1 | 2940402 | PG3603 |  |  |  | 33458877 |  | Scaffold | 01/06/2022 | JALZYU01 | 896637 | 1722966 | 147 | Illumina HiSeq | Dalian Medical University | PRJNA833221 | SAMN27963259 |  |  |  |
| *Aspergillus sp. PG3605* | GCA_023625635.1 | ASM2362563v1 | 2940388 | 2NP920 |  |  |  | 10640328 |  | Scaffold | 01/06/2022 | JAMAFM01 | 379293 | 885471 | 25 | Illumina HiSeq | Dalian Medical University | PRJNA833221 | SAMN27963589 |  |  |  |
| *Aspergillus sp. PG3606C* | GCA_023625255.1 | ASM2362525v1 | 2940427 | PG3606C |  |  |  | 33751973 |  | Scaffold | 01/06/2022 | JAMAAS01 | 1004141 | 1673420 | 239 | Illumina HiSeq | Dalian Medical University | PRJNA833221 | SAMN27963343 |  |  |  |
| *Aspergillus sp. PG3606D* | GCA_023619545.1 | ASM2361954v1 | 2940406 | PG3606D |  |  |  | 33454618 |  | Scaffold | 01/06/2022 | JALZYY01 | 899779 | 2484065 | 133 | Illumina HiSeq | Dalian Medical University | PRJNA833221 | SAMN27963263 |  |  |  |
| *Aspergillus sp. PG3608* | GCA_023619555.1 | ASM2361955v1 | 2940405 | PG3608 |  |  |  | 34896949 |  | Scaffold | 01/06/2022 | JALZYX01 | 1039770 | 1263560 | 247 | Illumina HiSeq | Dalian Medical University | PRJNA833221 | SAMN27963262 |  |  |  |
| *Aspergillus sp. PH2503* | GCA_023619285.1 | ASM2361928v1 | 2940419 | PH2503 |  |  |  | 34275684 |  | Scaffold | 01/06/2022 | JALZZL01 | 900593 | 2181589 | 137 | Illumina HiSeq | Dalian Medical University | PRJNA833221 | SAMN27963277 |  |  |  |
| *Aspergillus sp. PH3601* | GCA_023618865.1 | ASM2361886v1 | 2940425 | PH3601 |  |  |  | 33837860 |  | Scaffold | 01/06/2022 | JALZZR01 | 752856 | 1453242 | 132 | Illumina HiSeq | Dalian Medical University | PRJNA833221 | SAMN27963283 |  |  |  |
| *Aspergillus sp. PK2410* | GCA_023619325.1 | ASM2361932v1 | 2940417 | PK2410 |  |  |  | 34397072 |  | Scaffold | 01/06/2022 | JALZZJ01 | 651860 | 988032 | 164 | Illumina HiSeq | Dalian Medical University | PRJNA833221 | SAMN27963275 |  |  |  |
| *Aspergillus sp. PM3601* | GCA_023625295.1 | ASM2362529v1 | 2940426 | PM3601 |  |  |  | 34849926 |  | Scaffold | 01/06/2022 | JAMAAR01 | 1136876 | 1433906 | 205 | Illumina HiSeq | Dalian Medical University | PRJNA833221 | SAMN27963342 |  |  |  |
| *Aspergillus sp. PM3602* | GCA_023618995.1 | ASM2361899v1 | 2940421 | PM3602 |  |  |  | 33489266 |  | Scaffold | 01/06/2022 | JALZZN01 | 955300 | 1878732 | 58 | Illumina HiSeq | Dalian Medical University | PRJNA833221 | SAMN27963279 |  |  |  |
| *Aspergillus sp. PM3604* | GCA_025768815.1 | ASM2576881v1 | 2940327 | PG3605 |  |  |  | 34720046 |  | Scaffold | 20/10/2022 | JAMFNW01 | 327848 | 441586 | 526 | Illumina HiSeq | Dalian Medical University | PRJNA833221 | SAMN27963365 |  |  |  |
| *Aspergillus sp. PYS2105* | GCA_023619375.1 | ASM2361937v1 | 2940414 | PYS2105 |  |  |  | 34565978 |  | Scaffold | 01/06/2022 | JALZZG01 | 725943 | 1141547 | 200 | Illumina HiSeq | Dalian Medical University | PRJNA833221 | SAMN27963271 |  |  |  |
| *Aspergillus sp. PYS4201* | GCA_023627435.1 | ASM2362743v1 | 2940339 | S3505 |  |  |  | 25547250 |  | Scaffold | 01/06/2022 | JAMAHI01 | 164370 | 178399 | 349 | Illumina HiSeq | Dalian Medical University | PRJNA833221 | SAMN27963377 |  |  |  |
| *Aspergillus sp. PYS4240* | GCA_023627495.1 | ASM2362749v1 | 2940335 | S1802E |  |  |  | 25992996 |  | Scaffold | 01/06/2022 | JAMAHM01 | 261288 | 353686 | 208 | Illumina HiSeq | Dalian Medical University | PRJNA833221 | SAMN27963373 |  |  |  |
| *Aspergillus sp. PYS4241* | GCA_023627515.1 | ASM2362751v1 | 2940336 | PYS4240 |  |  |  | 25806729 |  | Scaffold | 01/06/2022 | JAMAHL01 | 197060 | 224678 | 357 | Illumina HiSeq | Dalian Medical University | PRJNA833221 | SAMN27963374 |  |  |  |
| *Aspergillus sp. R109-04A* | GCA_037042665.1 | ASM3704266v1 | 3092733 | R109-04A |  |  |  | 34175828 |  | Scaffold | 06/03/2024 | JAXQKU01 | 10718 | 10718 | 5547 | Illumina HiSeq | Dalian Medical University | PRJNA833221 | SAMN38095056 |  |  |  |
| *Aspergillus sp. R1430* | GCA_023625135.1 | ASM2362513v1 | 2940437 | R1430 |  |  |  | 35367792 |  | Scaffold | 01/06/2022 | JAMABC01 | 760713 | 1719712 | 235 | Illumina HiSeq | Dalian Medical University | PRJNA833221 | SAMN27963355 |  |  |  |
| *Aspergillus sp. R2302* | GCA_023627595.1 | ASM2362759v1 | 2940333 | R2407 |  |  |  | 35579069 |  | Scaffold | 01/06/2022 | JAMAHO01 | 433670 | 770397 | 395 | Illumina HiSeq | Dalian Medical University | PRJNA833221 | SAMN27963371 |  |  |  |
| *Aspergillus sp. R2305* | GCA_023627615.1 | ASM2362761v1 | 2940331 | R3607 |  |  |  | 34494604 |  | Scaffold | 01/06/2022 | JAMAHQ01 | 130552 | 159194 | 766 | Illumina HiSeq | Dalian Medical University | PRJNA833221 | SAMN27963369 |  |  |  |
| *Aspergillus sp. R2407* | GCA_023627575.1 | ASM2362757v1 | 2940332 | R2305 |  |  |  | 36382558 |  | Scaffold | 01/06/2022 | JAMAHP01 | 414552 | 831277 | 294 | Illumina HiSeq | Dalian Medical University | PRJNA833221 | SAMN27963370 |  |  |  |
| *Aspergillus sp. R2504* | GCA_023627395.1 | ASM2362739v1 | 2940341 | S1107B |  |  |  | 25588752 |  | Scaffold | 01/06/2022 | JAMAHG01 | 172469 | 185920 | 473 | Illumina HiSeq | Dalian Medical University | PRJNA833221 | SAMN27963379 |  |  |  |
| *Aspergillus sp. R2506* | GCA_023625075.1 | ASM2362507v1 | 2940434 | R2506 |  |  |  | 35264879 |  | Scaffold | 01/06/2022 | JAMAAZ01 | 843878 | 1114530 | 332 | Illumina HiSeq | Dalian Medical University | PRJNA833221 | SAMN27963352 |  |  |  |
| *Aspergillus sp. R2804* | GCA_023619725.1 | ASM2361972v1 | 2940398 | R2804 |  |  |  | 34532075 |  | Scaffold | 01/06/2022 | JALZYQ01 | 752255 | 1092826 | 249 | Illumina HiSeq | Dalian Medical University | PRJNA833221 | SAMN27963255 |  |  |  |
| *Aspergillus sp. R3602* | GCA_023618935.1 | ASM2361893v1 | 2940423 | R3602 |  |  |  | 33975846 |  | Scaffold | 01/06/2022 | JALZZP01 | 874560 | 1583862 | 72 | Illumina HiSeq | Dalian Medical University | PRJNA833221 | SAMN27963281 |  |  |  |
| *Aspergillus sp. R3607* | GCA_023627635.1 | ASM2362763v1 | 2940330 | F3603 |  |  |  | 34023850 |  | Scaffold | 01/06/2022 | JAMAHR01 | 739452 | 1192403 | 183 | Illumina HiSeq | Dalian Medical University | PRJNA833221 | SAMN27963368 |  |  |  |
| *Aspergillus sp. S1107B* | GCA_023627455.1 | ASM2362745v1 | 2940340 | PYS4201 |  |  |  | 25786915 |  | Scaffold | 01/06/2022 | JAMAHH01 | 187131 | 237520 | 348 | Illumina HiSeq | Dalian Medical University | PRJNA833221 | SAMN27963378 |  |  |  |
| *Aspergillus sp. S1702* | GCA_023619415.1 | ASM2361941v1 | 2940412 | S1702 |  |  |  | 34757653 |  | Scaffold | 01/06/2022 | JALZZE01 | 757185 | 1223947 | 131 | Illumina HiSeq | Dalian Medical University | PRJNA833221 | SAMN27963269 |  |  |  |
| *Aspergillus sp. S1801* | GCA_023627555.1 | ASM2362755v1 | 2940337 | PYS4241 |  |  |  | 25781678 |  | Scaffold | 01/06/2022 | JAMAHK01 | 202483 | 237522 | 343 | Illumina HiSeq | Dalian Medical University | PRJNA833221 | SAMN27963375 |  |  |  |
| *Aspergillus sp. S1802E* | GCA_023627535.1 | ASM2362753v1 | 2940334 | R2302 |  |  |  | 36431568 |  | Scaffold | 01/06/2022 | JAMAHN01 | 419866 | 762985 | 225 | Illumina HiSeq | Dalian Medical University | PRJNA833221 | SAMN27963372 |  |  |  |
| *Aspergillus sp. S1902Y* | GCA_023627675.1 | ASM2362767v1 | 2940328 | PM3604 |  |  |  | 33680840 |  | Scaffold | 01/06/2022 | JAMAHT01 | 917687 | 1307579 | 137 | Illumina HiSeq | Dalian Medical University | PRJNA833221 | SAMN27963366 |  |  |  |
| *Aspergillus sp. S1903S* | GCA_023619515.1 | ASM2361951v1 | 2940407 | S1903S |  |  |  | 33643979 |  | Scaffold | 01/06/2022 | JALZYZ01 | 573670 | 813688 | 259 | Illumina HiSeq | Dalian Medical University | PRJNA833221 | SAMN27963264 |  |  |  |
| *Aspergillus sp. S1905* | GCA_023619655.1 | ASM2361965v1 | 2940400 | S1905 |  |  |  | 33839119 |  | Scaffold | 01/06/2022 | JALZYS01 | 656476 | 860095 | 108 | Illumina HiSeq | Dalian Medical University | PRJNA833221 | SAMN27963257 |  |  |  |
| *Aspergillus sp. S2101G* | GCA_023625215.1 | ASM2362521v1 | 2940432 | S2101G |  |  |  | 35026519 |  | Scaffold | 01/06/2022 | JAMAAX01 | 964005 | 1476828 | 274 | Illumina HiSeq | Dalian Medical University | PRJNA833221 | SAMN27963350 |  |  |  |
| *Aspergillus sp. S2103* | GCA_023619405.1 | ASM2361940v1 | 2940413 | S2103 |  |  |  | 33613780 |  | Scaffold | 01/06/2022 | JALZZF01 | 513821 | 825362 | 326 | Illumina HiSeq | Dalian Medical University | PRJNA833221 | SAMN27963270 |  |  |  |
| *Aspergillus sp. S2105* | GCA_023619605.1 | ASM2361960v1 | 2940403 | S2105 |  |  |  | 33789765 |  | Scaffold | 01/06/2022 | JALZYV01 | 1437540 | 2385318 | 46 | Illumina HiSeq | Dalian Medical University | PRJNA833221 | SAMN27963260 |  |  |  |
| *Aspergillus sp. S2109* | GCA_023619645.1 | ASM2361964v1 | 2940401 | S2109 |  |  |  | 33541015 |  | Scaffold | 01/06/2022 | JALZYT01 | 1103419 | 1612122 | 205 | Illumina HiSeq | Dalian Medical University | PRJNA833221 | SAMN27963258 |  |  |  |
| *Aspergillus sp. S2111G.1* | GCA_023625175.1 | ASM2362517v1 | 2940431 | S2111G.1 |  |  |  | 34609756 |  | Scaffold | 01/06/2022 | JAMAAW01 | 829314 | 1267459 | 301 | Illumina HiSeq | Dalian Medical University | PRJNA833221 | SAMN27963349 |  |  |  |
| *Aspergillus sp. S2111Y* | GCA_023625095.1 | ASM2362509v1 | 2940436 | S2111Y |  |  |  | 35315504 |  | Scaffold | 01/06/2022 | JAMABB01 | 589884 | 1008974 | 276 | Illumina HiSeq | Dalian Medical University | PRJNA833221 | SAMN27963354 |  |  |  |
| *Aspergillus sp. S2805* | GCA_023627695.1 | ASM2362769v1 | 2940326 | S2805 |  |  |  | 33953665 |  | Scaffold | 01/06/2022 | JAMAHU01 | 607689 | 1156594 | 258 | Illumina HiSeq | Dalian Medical University | PRJNA833221 | SAMN27963364 |  |  |  |
| *Aspergillus sp. S3505* | GCA_023627475.1 | ASM2362747v1 | 2940338 | S1801 |  |  |  | 26022687 |  | Scaffold | 01/06/2022 | JAMAHJ01 | 310814 | 427903 | 169 | Illumina HiSeq | Dalian Medical University | PRJNA833221 | SAMN27963376 |  |  |  |
| *Aspergillus sp. S3602* | GCA_023625055.1 | ASM2362505v1 | 2940438 | S3602 |  |  |  | 33811123 |  | Scaffold | 01/06/2022 | JAMABD01 | 934052 | 1466719 | 227 | Illumina HiSeq | Dalian Medical University | PRJNA833221 | SAMN27963356 |  |  |  |
| *Aspergillus sp. S3605* | GCA_023625235.1 | ASM2362523v1 | 2940429 | S3605 |  |  |  | 33776036 |  | Scaffold | 01/06/2022 | JAMAAU01 | 1034186 | 1542327 | 217 | Illumina HiSeq | Dalian Medical University | PRJNA833221 | SAMN27963346 |  |  |  |
| *Aspergillus sp. S3606* | GCA_023619495.1 | ASM2361949v1 | 2940408 | S3606 |  |  |  | 33818669 |  | Scaffold | 01/06/2022 | JALZZA01 | 1038156 | 1807989 | 58 | Illumina HiSeq | Dalian Medical University | PRJNA833221 | SAMN27963265 |  |  |  |
| *Aspergillus sp. S4401N* | GCA_023619365.1 | ASM2361936v1 | 2940415 | S4401N |  |  |  | 32947211 |  | Scaffold | 01/06/2022 | JALZZH01 | 490031 | 716835 | 263 | Illumina HiSeq | Dalian Medical University | PRJNA833221 | SAMN27963272 |  |  |  |
| *Aspergillus sp. S4402* | GCA_023619295.1 | ASM2361929v1 | 2940418 | S4402 |  |  |  | 33331040 |  | Scaffold | 01/06/2022 | JALZZK01 | 605863 | 739088 | 135 | Illumina HiSeq | Dalian Medical University | PRJNA833221 | SAMN27963276 |  |  |  |
| *Aspergillus sp. Z5* | GCA_025765985.1 | ASM2576598v1 | 1662659 | PG40-03 |  |  |  | 33801708 |  | Scaffold | 20/10/2022 | JAMFMN01 | 1055577 | 2238380 | 59 | Illumina HiSeq | Dalian Medical University | PRJNA833221 | SAMN27963274 |  |  |  |
| *Aspergillus sparsus* | GCA_027569235.1 | ASM2756923v1 | 37232 | NRRL 1933 |  |  |  | 38868395 |  | Contig | 04/01/2023 | JAPDES01 | 356457 |  |  | Illumina NovaSeq | Northwestern University | PRJNA852164 | SAMN31355050 |  |  |  |
| *Aspergillus spinulosporus* | GCA_003574815.1 | ASM357481v1 | 1810908 | NRRL 2395 |  |  |  | 33169175 |  | Scaffold | 18/09/2018 | QPHH01 | 312007 | 939600 | 5527 | Illumina HiSeq | Vanderbilt University | PRJNA481010 | SAMN09654975 |  |  |  |
| *Aspergillus steynii IBT 23096* | GCF_002849105.1 | Aspste1 | 1392250 | IBT 23096 |  |  | Annotation submitted by DOE Joint Genome Institute | 37847960 |  | Contig | 30/12/2017 | MSFO01 | 3921250 |  |  | PacBio | DOE Joint Genome Institute | PRJNA217208 | SAMN02744649 | 13148 | 12919 | 11 |
| *Aspergillus subversicolor* | GCA_037044295.1 | ASM3704429v1 | 1220208 | LH_A137 |  |  |  | 43917974 |  | Contig | 06/03/2024 | JAWLTM01 | 1206464 |  |  | Illumina | Qingdao Agricultural University | PRJNA1025352 | SAMN37864985 |  |  |  |
| *Aspergillus sydowii CBS 593.65* | GCF_001890705.1 | Aspsy1 | 1036612 | CBS 593.65 |  |  | Annotation submitted by DOE Joint Genome Institute | 34381026 |  | Scaffold | 08/12/2016 | MRCH01 | 123783 | 2288531 | 97 | Illumina | DOE Joint Genome Institute | PRJNA207689 | SAMN02744090 | 13717 | 13579 | 1 |
| *Aspergillus taichungensis* | GCA_002850765.1 | Asptaic1 | 482145 | IBT 19404 |  |  | Annotation submitted by DOE Joint Genome Institute | 27121736 |  | Scaffold | 03/01/2018 | PKFW01 | 156925 | 207690 | 310 | Illumina | DOE Joint Genome Institute | PRJNA334013 | SAMN05446796 | 9852 | 9689 | 1 |
| *Aspergillus tamarii* | GCA_030556085.1 | ASM3055608v1 | 41984 | TPD11 |  |  |  | 38807518 |  | Contig | 31/07/2023 | JARVTJ01 | 5335885 |  |  | PacBio | Yunnan Agricultural University | PRJNA939967 | SAMN33550032 |  |  |  |
| *Aspergillus tanneri* | GCF_003426965.1 | ASM342696v1 | 1220188 | NIH1004 |  |  | Annotation submitted by JCVI | 38719388 |  | Contig | 24/08/2018 | QUQM01 | 4499170 |  |  | Oxford Nanopore MinION | JCVI | PRJNA239015 | SAMN09761659 | 11845 | 11682 |  |
| *Aspergillus tennesseensis* | GCA_037043975.1 | ASM3704397v1 | 1220210 | LH_A417 |  |  |  | 33471102 |  | Contig | 06/03/2024 | JAWLSW01 | 627416 |  |  | Illumina | Qingdao Agricultural University | PRJNA1025352 | SAMN37865001 |  |  |  |
| *Aspergillus terreus NIH2624* | GCF_000149615.1 | ASM14961v1 | 341663 | NIH2624 |  |  | Annotation submitted by Broad Institute | 29331195 |  | Scaffold | 08/09/2006 | AAJN01 | 224147 | 1912493 | 26 |  | Broad Institute | PRJNA15631 | SAMN02953635 | 10551 | 10401 | 3 |
| *Aspergillus texensis* | GCA_024668715.1 | ASM2466871v1 | 2495910 | TX-A-6-1-S |  |  |  | 38950007 |  | Scaffold | 15/08/2022 | JANAXO01 | 188864 | 188864 | 670 | Illumina | University of Wisconsin Madison | PRJNA639008 | SAMN15220958 |  |  |  |
| *Aspergillus thermomutatus* | GCF_002237265.1 | ASM223726v2 | 41047 | HMR AF 39 |  |  | Annotation submitted by University of Montreal | 30945974 |  | Contig | 12/09/2018 | NKHU02 | 93306 |  |  | Illumina MiSeq | University of Montreal | PRJNA388488 | SAMN07177963 | 9701 | 9701 |  |
| *Aspergillus transmontanensis* | GCA_009193505.1 | Asptra1 | 1034304 | CBS 130015 |  |  | Annotation submitted by DOE Joint Genome Institute | 39338775 |  | Scaffold | 18/10/2019 | STFK01 | 257683 | 342792 | 293 | Illumina | DOE Joint Genome Institute | PRJNA333913 | SAMN05446753 | 14465 | 14215 | 2 |
| *Aspergillus tubingensis* | GCF_013340325.1 | ASM1334032v1 | 5068 | WU-2223L |  |  | Annotation submitted by Waseda University | 35047229 |  | Scaffold | 09/06/2020 | BLWE01 | 3153774 | 3153774 | 15 | HiSeq X; Nanopore MinION | Waseda University | PRJDB9560 | SAMD00216538 | 11809 | 11476 |  |
| *Aspergillus turcosus* | GCA_002234965.2 | ASM223496v2 | 1245748 | HMR AF 23 |  |  | Annotation submitted by University of Montreal | 32352653 |  | Contig | 12/09/2018 | NKHV02 | 110871 |  |  | Illumina MiSeq | University of Montreal | PRJNA388488 | SAMN07177962 | 9223 | 9223 |  |
| *Aspergillus udagawae* | GCF_001078395.1 | Aud_assembly02 | 91492 | IFM 46973 |  |  | Annotation submitted by Medical Mycology Research Center | 32252303 |  | Scaffold | 12/03/2021 | BBXM02 | 893916 | 4123300 | 17 | Illumina HiSeq 2500 | Medical Mycology Research Center | PRJDB3949 | SAMD00031778 | 11010 | 10796 |  |
| *Aspergillus unguis* | GCA_018408605.1 | ASM1840860v1 | 40381 | F6_8S_P_4A |  |  |  | 25892532 |  | Scaffold | 19/05/2021 | JAGUQC01 | 1808513 | 2741542 | 19 | Illumina NovaSeq | Jet Propulsion Laboratory, California Institute of Technology | PRJNA723004 | SAMN18883804 |  |  |  |
| *Aspergillus ustus* | GCA_040285355.1 | ASM4028535v1 | 40382 | AUSTUSMSU | Russia |  |  | 42011276 |  | Contig | 24/06/2024 | JBCHKC01 | 405810 |  |  | Illumina NovaSeq | Lomonosov MSU | PRJNA1089602 | SAMN40546144 |  |  |  |
| *Aspergillus uvarum CBS 121591* | GCF_003184745.1 | Aspuva1 | 1448315 | CBS 121591 |  |  | Annotation submitted by DOE Joint Genome Institute | 35850703 |  | Scaffold | 04/06/2018 | MSFT01 | 276704 | 396097 | 172 | Illumina | DOE Joint Genome Institute | PRJNA235077 | SAMN05660759 | 12347 | 12014 | 4 |
| *Aspergillus vadensis CBS 113365* | GCF_003184925.1 | Aspvad1 | 1448311 | CBS 113365 |  |  | Annotation submitted by DOE Joint Genome Institute | 35663374 |  | Scaffold | 04/06/2018 | MSFS01 | 673643 | 1025125 | 60 | Illumina | DOE Joint Genome Institute | PRJNA235071 | SAMN05661105 | 12404 | 12132 | 7 |
| *Aspergillus verrucosus* | GCA_039955225.1 | Aver_NBRC115547_1.0 | 3033834 | NBRC 115547 |  |  |  | 27279305 |  | Scaffold | 24/02/2024 | BAABNM01 | 137840 | 141380 | 411 | DNBSEQ-G400 sequencer | School of Pharmacy, Nihon University | PRJDB17030 | SAMD00657602 |  |  |  |
| *Aspergillus versicolor CBS 583.65* | GCF_001890125.1 | Aspve1 | 1036611 | CBS 583.65 |  |  | Annotation submitted by DOE Joint Genome Institute | 33126810 |  | Scaffold | 06/12/2016 | MRBN01 | 695565 | 2487993 | 51 | Illumina | DOE Joint Genome Institute | PRJNA207687 | SAMN02744095 | 13364 | 13222 | 1 |
| *Aspergillus violaceofuscus CBS 115571* | GCA_003184705.1 | Aspvio1 | 1450538 | CBS 115571 |  |  | Annotation submitted by DOE Joint Genome Institute | 36012865 |  | Scaffold | 04/06/2018 | PSTA01 | 197877 | 288343 | 209 | Illumina | DOE Joint Genome Institute | PRJNA235060 | SAMN05660314 | 12415 | 12080 | 6 |
| *Aspergillus viridinutans* | GCF_018404265.1 | Aspvir_assembly01 | 75553 | IFM 47045 |  |  | Annotation submitted by Medical Mycology Research Center | 34888349 |  | Scaffold | 12/03/2021 | BOPL01 | 509684 | 2861404 | 47 | Illumina HiSeq 2500 | Medical Mycology Research Center | PRJDB8156 | SAMD00275554 | 10238 | 10039 |  |
| *Aspergillus welwitschiae* | GCF_003344945.1 | Aspwel1 | 1341132 | CBS 139.54b |  |  | Annotation submitted by DOE Joint Genome Institute | 37511876 |  | Scaffold | 30/07/2018 | QQZQ01 | 365683 | 757052 | 396 | Illumina | DOE Joint Genome Institute | PRJNA333467 | SAMN05446521 | 13957 | 13684 | 5 |
| *Aspergillus wentii DTO 134E9* | GCF_001890725.1 | Aspwe1 | 1073089 | DTO 134E9 |  |  | Annotation submitted by DOE Joint Genome Institute | 31350635 |  | Scaffold | 08/12/2016 | LJSE01 | 679749 | 4128426 | 27 | Illumina | DOE Joint Genome Institute | PRJNA170886 | SAMN02744089 | 12658 | 12434 | 3 |
| *Aspergillus westerdijkiae* | GCA_019976475.1 | ASM1997647v1 | 357447 | IBT 35663 |  |  |  | 36038104 |  | Contig | 14/09/2021 | JAFBMQ01 | 4855597 |  |  | Oxford Nanopore MinION | Aalborg university | PRJNA698789 | SAMN17736565 |  |  |  |
| *Byssochlamys sp. AF001* | GCA_002914405.1 | ByssAF2.0 | 2066500 | AF001 |  |  |  | 35878077 |  | Contig | 02/02/2018 | PNEM01 | 4599098 |  |  | Illumina; Oxford Nanopore | University of Oklahoma | PRJNA427363 | SAMN08235907 |  |  |  |
| *Byssochlamys sp. BYSS01* | GCA_002242795.1 | ASM224279v1 | 2059436 | BYSS01 |  |  |  | 29074643 |  | Scaffold | 07/08/2017 | NIXA01 | 456333 | 463366 | 394 | Illumina HiSeq | University of Dayton Research Institute | PRJNA390431 | SAMN07234396 |  |  |  |
| *Byssochlamys sp. IMV 00236* | GCA_001931875.2 | ASM193187v2 | 2562441 | IMV 00236 |  |  |  | 47573060 |  | Scaffold | 15/02/2017 | MSJH02 | 57106 | 186555 | 843 | Illumina HiSeq | Jet Propulsion Laboratory, California Institute of Technology | PRJNA355122 | SAMN06076682 |  |  |  |
| *Elaphomyces granulatus* | GCA_002240705.1 | PX439 | 519963 | OSC145934 |  |  | Annotation submitted by Oregon State University | 54153615 |  | Scaffold | 04/08/2017 | NPHW01 | 10248 | 11798 | 8670 | Illumina HiSeq | Oregon State University | PRJNA248240 | SAMN02796050 | 7171 | 7171 |  |
| *Evansstolkia leycettana* | GCA_000787455.1 | ASM78745v1 | 196907 | CBS 398.68 |  |  |  | 25946254 |  | Contig | 26/11/2014 | JSYV01 | 58797 |  |  | Illumina HiSeq | none | PRJNA261108 | SAMN03068951 |  |  |  |
| *Hamigera avellanea* | GCA_027569715.1 | ASM2756971v1 | 39317 | NRRL 1938 |  |  |  | 28294013 |  | Contig | 04/01/2023 | JAPDKU01 | 243105 |  |  | Illumina NovaSeq | Northwestern University | PRJNA852164 | SAMN31355054 |  |  |  |
| *Monascus pilosus* | GCA_018806995.1 | ASM1880699v1 | 89488 | MS-1 |  |  |  | 26196030 |  | Contig | 09/06/2021 | JAGHLQ01 | 3510661 |  |  | Illumina NovaSeq; PacBio Sequel | Huazhong Agricultural University | PRJNA718072 | SAMN18521630 |  |  |  |
| *Monascus purpureus* | GCA_025999795.1 | ASM2599979v1 | 5098 | KUPM5 |  |  | Annotation submitted by The United Graduate School of Agricultural Sciences Kagoshima University | 24475985 | 7 | Chromosome | 06/10/2022 |  | 3075987 | 3075987 | 10 | MinION; Illumina Novaseq 6000 | The United Graduate School of Agricultural Sciences Kagoshima University | PRJDB11993 | SAMD00393899 | 9270 | 9151 |  |
| *Monascus ruber* | GCA_002976275.1 | ASM297627v1 | 89489 | FWB13 |  |  |  | 26294404 |  | Contig | 07/03/2018 | PSNO01 | 3364862 |  |  | Illumina HiSeq | Fujian Institute of Microbiology | PRJNA433431 | SAMN08493478 |  |  |  |
| *Paecilomyces dactylethromorphus* | GCA_029606265.1 | ASM2960626v1 | 644134 | FRR 5262 |  |  |  | 34555934 |  | Scaffold | 06/04/2023 | JAPVCE01 | 145927 | 2439234 | 43 | Illumina | University of Melbourne | PRJNA604095 | SAMN32135774 |  |  |  |
| *Paecilomyces fulvus* | GCA_029606305.1 | ASM2960630v1 | 89137 | FRR 3794 |  |  |  | 31519628 |  | Scaffold | 06/04/2023 | JAPVCG01 | 82363 | 835778 | 76 | Illumina | University of Melbourne | PRJNA604095 | SAMN32135772 |  |  |  |
| *Paecilomyces lecythidis* | GCA_029606225.1 | ASM2960622v1 | 3004212 | FRR 4481 |  |  |  | 32040345 |  | Scaffold | 06/04/2023 | JAPVCC01 | 176878 | 3687581 | 31 | Illumina | University of Melbourne | PRJNA604095 | SAMN32135776 |  |  |  |
| *Paecilomyces maximus* | GCA_029606185.1 | ASM2960618v1 | 644133 | FRR 3793 |  |  |  | 29561917 |  | Scaffold | 06/04/2023 | JAPVCB01 | 113799 | 1721955 | 42 | Illumina | University of Melbourne | PRJNA604095 | SAMN32135777 |  |  |  |
| *Paecilomyces niveus* | GCA_003116535.1 | ASM311653v1 | 5093 | Cornell orchards no. 7 |  |  |  | 36018796 |  | Scaffold | 11/05/2018 | QEIL01 | 185295 | 185295 | 584 | Illumina MiSeq | Cornell University | PRJNA454024 | SAMN08996309 |  |  |  |
| *Paecilomyces variotii* | GCF_004022145.1 | Paevar1 | 264951 | CBS 101075 |  |  | Annotation submitted by DOE Joint Genome Institute | 30105809 |  | Contig | 14/01/2019 | RCNU01 | 1732371 |  |  | PacBio | DOE Joint Genome Institute | PRJNA444022 | SAMN08778357 | 9415 | 9270 | 3 |
| *Penicilliopsis zonata CBS 506.65* | GCF_001890105.1 | Aspzo1 | 1073090 | CBS 506.65 |  |  | Annotation submitted by DOE Joint Genome Institute | 26088231 |  | Scaffold | 08/12/2016 | MRBM01 | 188319 | 1215459 | 246 | Illumina | DOE Joint Genome Institute | PRJNA207688 | SAMN02194405 | 10027 | 9869 | 3 |
| *Penicillium alfredii* | GCF_028826965.1 | ASM2882696v1 | 1506179 | IBT 34128 |  |  | Annotation submitted by Aalborg University | 27430394 |  | Contig | 23/02/2023 | JAPMSZ01 | 3327006 |  |  | Oxford Nanopore MinION | Aalborg University | PRJNA867151 | SAMN30185292 | 10161 | 10161 |  |
| *Penicillium angulare* | GCF_028827245.1 | ASM2882724v1 | 116970 | IBT 27051 |  |  | Annotation submitted by Aalborg University | 37782843 |  | Contig | 23/02/2023 | JAPZBL01 | 5070470 |  |  | Oxford Nanopore MinION | Aalborg University | PRJNA867151 | SAMN30185293 | 13390 | 13389 | 1 |
| *Penicillium antarcticum* | GCF_028974205.1 | ASM2897420v1 | 416450 | IBT 31339 |  |  | Annotation submitted by Aalborg University | 30836278 |  | Contig | 23/02/2023 | JAPZBM01 | 3813293 |  |  | Oxford Nanopore MinION | Aalborg University | PRJNA867151 | SAMN30185295 | 11212 | 11212 |  |
| *Penicillium argentinense* | GCF_028826775.1 | ASM2882677v1 | 1131581 | IBT 30761 |  |  | Annotation submitted by Aalborg University | 33838379 |  | Contig | 23/02/2023 | JAPQKI01 | 5062079 |  |  | Oxford Nanopore MinION | Aalborg University | PRJNA867151 | SAMN30185296 | 12143 | 12143 |  |
| *Penicillium arizonense* | GCF_001773325.1 | ASM177332v1 | 1835702 | CBS 141311 |  |  | Annotation submitted by Chalmers University of Technology | 33729052 |  | Scaffold | 18/10/2016 | LXJU01 | 681260 | 1033882 | 396 | Illumina HiSeq | Chalmers University of Technology | PRJNA318735 | SAMN04884538 | 12200 | 12200 |  |
| *Penicillium atrosanguineum* | GCF_028827265.1 | ASM2882726v1 | 1132637 | IBT 20685 |  |  | Annotation submitted by Aalborg University | 28612168 |  | Contig | 23/02/2023 | JAPZBN01 | 3894271 |  |  | Oxford Nanopore MinION | Aalborg University | PRJNA867151 | SAMN30185297 | 10999 | 10997 | 2 |
| *Penicillium aurantiogriseum* | GCA_019977855.1 | ASM1997785v1 | 36655 | IBT 35659 |  |  |  | 32600911 |  | Contig | 14/09/2021 | JAFCIW01 | 9159201 |  |  | Oxford Nanopore MinION | Aalborg university | PRJNA698798 | SAMN17736664 |  |  |  |
| *Penicillium bialowiezense* | GCA_026122735.1 | ASM2612273v1 | 293381 | A30 |  |  |  | 31166888 |  | Contig | 10/11/2022 | JAKZFB01 | 4926553 |  |  | Illumina; Oxford Nanopore | Shanghai Jiao Tong University | PRJNA811710 | SAMN26350203 |  |  |  |
| *Penicillium biforme FM169* | GCA_000577785.1 | PBIFFM169_20131217 | 1439349 | FM169 |  |  |  | 34874608 |  | Scaffold | 20/02/2014 | CBXO01 | 195600 | 277312 | 582 |  | INRA-LIPM | PRJEB5118 | SAMEA3138938 |  |  |  |
| *Penicillium bovifimosum* | GCF_028826915.1 | ASM2882691v1 | 126998 | IBT 22155 |  |  | Annotation submitted by Aalborg University | 27188871 |  | Contig | 23/02/2023 | JAPQKL01 | 3348542 |  |  | Oxford Nanopore MinION | Aalborg University | PRJNA867151 | SAMN30185302 | 10403 | 10402 | 1 |
| *Penicillium brasilianum* | GCA_001048715.1 | Pbras_Allpaths-LG | 104259 |  |  |  | Annotation submitted by HKI JENA | 35888622 |  | Scaffold | 01/07/2015 | CDHK01 | 653379 | 3363074 | 87 |  | HKI JENA | PRJEB7514 | SAMEA2820536 | 11432 | 11432 |  |
| *Penicillium brefeldianum* | GCA_040333145.1 | ASM4033314v1 | 1131482 | F032 |  |  |  | 33894595 |  | Contig | 26/06/2024 | JBEBNH01 | 3974671 |  |  | BGISEQ | BGI-Qingdao | PRJNA1116794 | SAMN41560906 |  |  |  |
| *Penicillium brevicompactum* | GCF_028827555.1 | ASM2882755v1 | 5074 | IBT 35665 |  |  | Annotation submitted by Aalborg University | 34726155 |  | Contig | 23/02/2023 | JAPZBP01 | 6130730 |  |  | Oxford Nanopore MinION | Aalborg University | PRJNA867151 | SAMN30185303 | 12371 | 12367 | 4 |
| *Penicillium camemberti* | GCA_014839975.1 | PcamFM013r2_polished | 5075 | FM013=LCP06093 |  |  |  | 35346490 |  | Contig | 30/09/2020 | JACXYV01 | 1535773 |  |  | PacBio RSII | Universite Paris Saclay / CNRS | PRJNA655754 | SAMN16094528 |  |  |  |
| *Penicillium canariense* | GCF_028826845.1 | ASM2882684v1 | 189055 | IBT 26290 |  |  | Annotation submitted by Aalborg University | 31521451 |  | Contig | 23/02/2023 | JAPQKN01 | 4795020 |  |  | Oxford Nanopore MinION | Aalborg University | PRJNA867151 | SAMN30185312 | 10805 | 10805 |  |
| *Penicillium canescens* | GCF_028828765.1 | ASM2882876v1 | 5083 | IBT 15451 |  |  | Annotation submitted by Aalborg University | 46284494 |  | Contig | 23/02/2023 | JAQJZN01 | 4366774 |  |  | Oxford Nanopore MinION | Aalborg University | PRJNA867151 | SAMN30185308 | 14207 | 14180 |  |
| *Penicillium capsulatum* | GCA_028828875.1 | ASM2882887v1 | 69766 | IBT 29712 |  |  | Annotation submitted by Aalborg University | 27651227 |  | Contig | 23/02/2023 | JAQJZR01 | 4707077 |  |  | Oxford Nanopore MinION | Aalborg University | PRJNA867151 | SAMN30185350 | 10633 | 10633 |  |
| *Penicillium carneum LCP05634* | GCA_000577495.1 | PCARLCP05634_20131217 | 1439351 | LCP05634 |  |  |  | 25936550 |  | Scaffold | 20/02/2014 | CBXS01 | 11757 | 24373 | 2090 |  | INRA-LIPM | PRJEB5124 | SAMEA3138930 |  |  |  |
| *Penicillium caseifulvum* | GCA_022813165.1 | ASM2281316v1 | 293374 | FKI-L3-CM-P1 |  |  |  | 35881231 |  | Scaffold | 04/04/2022 | JAKLNB01 | 154820 | 221716 | 824 | Illumina NovaSeq | Jet Propulsion Laboratory, California Institute of Technology | PRJNA800051 | SAMN25226808 |  |  |  |
| *Penicillium cataractarum* | GCF_028827025.1 | ASM2882702v1 | 2100454 | IBT 29864 |  |  | Annotation submitted by Aalborg University | 37446941 |  | Contig | 23/02/2023 | JAPZBS01 | 4659271 |  |  | Oxford Nanopore MinION | Aalborg University | PRJNA867151 | SAMN30185314 | 12828 | 12825 | 3 |
| *Penicillium cf. chrysogenum* | GCA_019827475.1 | ASM1982747v1 | 3062267 | S/N-308-OC-P1 |  |  |  | 68790985 |  | Scaffold | 31/08/2021 | JACVQR01 | 386041 | 692322 | 893 | Illumina | Jet Propulsion Laboratory, California Institute of Technology | PRJNA644637 | SAMN15793546 |  |  |  |
| *Penicillium cf. griseofulvum* | GCA_028974015.1 | ASM2897401v1 | 2972120 | IBT 16849 |  |  | Annotation submitted by Aalborg University | 29823847 |  | Contig | 23/02/2023 | JAPQKP01 | 5598030 |  |  | Oxford Nanopore MinION | Aalborg University | PRJNA867151 | SAMN30185357 | 11336 | 11334 | 2 |
| *Penicillium cf. viridicatum* | GCA_028974045.1 | ASM2897404v1 | 2972119 | IBT 20477 |  |  | Annotation submitted by Aalborg University | 35226502 |  | Contig | 23/02/2023 | JAPQKQ01 | 4582560 |  |  | Oxford Nanopore MinION | Aalborg University | PRJNA867151 | SAMN30185316 | 12613 | 12613 |  |
| *Penicillium chermesinum* | GCF_028974085.1 | ASM2897408v1 | 63820 | IBT 19713 |  |  | Annotation submitted by Aalborg University | 27581382 |  | Contig | 23/02/2023 | JAPQKS01 | 4118209 |  |  | Oxford Nanopore MinION | Aalborg University | PRJNA867151 | SAMN30185315 | 10821 | 10820 | 1 |
| *Penicillium chrysogenum* | GCF_028827035.1 | ASM2882703v1 | 5076 | IBT 35668 |  |  | Annotation submitted by Aalborg University | 32379777 |  | Contig | 23/02/2023 | JAPQKU01 | 9490807 |  |  | Oxford Nanopore MinION | Aalborg University | PRJNA867151 | SAMN30185319 | 11975 | 11974 | 1 |
| *Penicillium cinerascens* | GCF_028974065.1 | ASM2897406v1 | 70096 | IBT 15544 |  |  | Annotation submitted by Aalborg University | 29467041 |  | Contig | 23/02/2023 | JAPQKR01 | 3994343 |  |  | Oxford Nanopore MinION | Aalborg University | PRJNA867151 | SAMN30185320 | 11023 | 11022 | 1 |
| *Penicillium citrinum* | GCF_028827155.1 | ASM2882715v1 | 5077 | IBT 23319 |  |  | Annotation submitted by Aalborg University | 31396546 |  | Contig | 23/02/2023 | JAPQKT01 | 3834662 |  |  | Oxford Nanopore MinION | Aalborg University | PRJNA867151 | SAMN30185321 | 11664 | 11664 |  |
| *Penicillium coffeae* | GCA_035985395.1 | ASM3598539v1 | 310288 |  |  | ANU01 |  | 30909372 | 7 | Complete Genome | 23/01/2024 |  | 4566672 | 4566672 | 7 | Oxford Nanopore MinION | Australia National University | PRJNA1063332 | SAMN39335299 |  |  |  |
| *Penicillium concentricum* | GCF_028827145.1 | ASM2882714v1 | 293559 | IBT 3081 |  |  | Annotation submitted by Aalborg University | 29963931 |  | Contig | 23/02/2023 | JAPZBT01 | 9135964 |  |  | Oxford Nanopore MinION | Aalborg University | PRJNA867151 | SAMN30185322 | 11702 | 11702 |  |
| *Penicillium coprophilum* | GCF_028826855.1 | ASM2882685v1 | 36646 | IBT 35676 |  |  | Annotation submitted by Aalborg University | 29014640 |  | Contig | 23/02/2023 | JAPQKM01 | 4172608 |  |  | Oxford Nanopore MinION | Aalborg University | PRJNA867151 | SAMN30185323 | 10983 | 10982 | 1 |
| *Penicillium corylophilum* | GCA_018410145.1 | ASM1841014v1 | 70792 | F5_1S_1A_F |  |  |  | 28229796 |  | Scaffold | 19/05/2021 | JAGUQL01 | 1383961 | 1725911 | 53 | Illumina NovaSeq | Jet Propulsion Laboratory, California Institute of Technology | PRJNA723004 | SAMN18883757 |  |  |  |
| *Penicillium cosmopolitanum* | GCF_028827165.1 | ASM2882716v1 | 1131564 | IBT 29677 |  |  | Annotation submitted by Aalborg University | 40034523 |  | Contig | 23/02/2023 | JAPZBU01 | 4365462 |  |  | Oxford Nanopore MinION | Aalborg University | PRJNA867151 | SAMN30185324 | 14288 | 14273 | 15 |
| *Penicillium crustosum* | GCF_028827405.1 | ASM2882740v1 | 36656 | IBT 35664 |  |  | Annotation submitted by Aalborg University | 32953279 |  | Contig | 23/02/2023 | JAPZBV01 | 9362940 |  |  | Oxford Nanopore MinION | Aalborg University | PRJNA867151 | SAMN30185325 | 12343 | 12313 | 30 |
| *Penicillium crystallinum* | GCA_027569675.1 | ASM2756967v1 | 69804 | NRRL 5082 |  |  |  | 27069658 |  | Contig | 04/01/2023 | JAPDKR01 | 334678 |  |  | Illumina NovaSeq | Northwestern University | PRJNA852164 | SAMN31355041 |  |  |  |
| *Penicillium daleae* | GCF_028827525.1 | ASM2882752v1 | 63821 | IBT 16125 |  |  | Annotation submitted by Aalborg University | 39657045 |  | Contig | 23/02/2023 | JAPVEA01 | 4752356 |  |  | Oxford Nanopore MinION | Aalborg University | PRJNA867151 | SAMN30185326 | 12824 | 12821 | 3 |
| *Penicillium decumbens* | GCA_027569385.1 | ASM2756938v1 | 69771 | NRRL 741 |  |  |  | 24142420 |  | Contig | 04/01/2023 | JAPDEZ01 | 831129 |  |  | Illumina NovaSeq | Northwestern University | PRJNA852164 | SAMN31355059 |  |  |  |
| *Penicillium desertorum* | GCA_028827375.1 | ASM2882737v1 | 1303715 | IBT 17660 |  |  | Annotation submitted by Aalborg University | 33986870 |  | Contig | 23/02/2023 | JAPWDO01 | 5473808 |  |  | Oxford Nanopore MinION | Aalborg University | PRJNA867151 | SAMN30185327 | 13049 | 13047 | 2 |
| *Penicillium diatomitis* | GCF_028827545.1 | ASM2882754v1 | 2819901 | IBT 30728 |  |  | Annotation submitted by Aalborg University | 33965862 |  | Contig | 23/02/2023 | JAPWDQ01 | 3372961 |  |  | Oxford Nanopore MinION | Aalborg University | PRJNA867151 | SAMN30185328 | 9582 | 9580 | 2 |
| *Penicillium digitatum* | GCF_016767815.1 | ASM1676781v1 | 36651 | PdW03 |  |  | Annotation submitted by Hangzhou Normal University | 26336737 | 6 | Complete Genome | 28/01/2021 |  | 4123518 | 4123518 | 6 | PacBio; Illumina | Hangzhou Normal University | PRJNA658435 | SAMN04018713 | 9238 | 9002 |  |
| *Penicillium dipodomyicola* | GCA_015585785.1 | ASM1558578v1 | 254876 | IIF7SW-F2 |  |  |  | 32111153 |  | Scaffold | 18/11/2020 | JACSOR01 | 546983 | 842068 | 431 | Illumina NovaSeq | Jet Propulsion Laboratory, California Institute of Technology | PRJNA659567 | SAMN15915653 |  |  |  |
| *Penicillium egyptiacum* | GCA_911456345.1 | Pegy_LCP06446 | 1303716 |  |  |  | Annotation submitted by University Paris-Sud | 32674410 |  | Scaffold | 22/09/2022 | CAJVRC01 | 81332 | 1200179 | 909 |  | University Paris-Sud | PRJEB44534 | SAMEA8618056 | 11062 | 10635 |  |
| *Penicillium expansum* | GCF_000769745.1 | ASM76974v1 | 27334 | MD-8 |  |  | Annotation submitted by Center for Genomic Regulation (CRG) | 32356048 |  | Contig | 28/10/2014 | JQFZ01 | 301353 |  |  | Illumina HiSeq | Center for Genomic Regulation (CRG) | PRJNA255747 | SAMN02928573 | 11060 | 11060 |  |
| *Penicillium fimorum* | GCA_028828255.1 | ASM2882825v1 | 1882269 | IBT 29495 |  |  | Annotation submitted by Aalborg University | 28556969 |  | Contig | 23/02/2023 | JAPWDS01 | 8023479 |  |  | Oxford Nanopore MinION | Aalborg University | PRJNA867151 | SAMN30185330 | 10990 | 10989 | 1 |
| *Penicillium flavigenum* | GCA_002072365.1 | ASM207236v1 | 254877 | IBT 14082 |  |  | Annotation submitted by Chalmers University of Technology | 32903267 |  | Scaffold | 24/03/2017 | MLQL01 | 305944 | 607994 | 260 | Illumina HiSeq | Chalmers University of Technology | PRJNA318735 | SAMN05200882 | 10994 | 10994 |  |
| *Penicillium freii* | GCA_028827385.1 | ASM2882738v1 | 48697 | IBT 34325 |  |  | Annotation submitted by Aalborg University | 35356366 |  | Contig | 23/02/2023 | JAQIZX01 | 8219394 |  |  | Oxford Nanopore MinION | Aalborg University | PRJNA867151 | SAMN30185331 | 12256 | 12256 |  |
| *Penicillium frequentans* | GCA_028828275.1 | ASM2882827v1 | 3151616 | IBT 35677 |  |  | Annotation submitted by Aalborg University | 38045409 |  | Contig | 23/02/2023 | JAQIZY01 | 4680361 |  |  | Oxford Nanopore MinION | Aalborg University | PRJNA867151 | SAMN30185332 | 13411 | 13411 |  |
| *Penicillium fructuariae-cellae* | GCA_037044005.1 | ASM3704400v1 | 2819925 | LH_A412 |  |  |  | 34656616 |  | Contig | 06/03/2024 | JAWLSY01 | 295896 |  |  | Illumina | Qingdao Agricultural University | PRJNA1025352 | SAMN37864999 |  |  |  |
| *Penicillium fuscoglaucum* | GCA_040250155.1 | Pf_T2 | 463681 |  |  | Pf_T2 |  | 35121114 | 5 | Complete Genome | 19/06/2024 |  | 9118168 | 9118168 | 5 | Oxford Nanopore MinION | USDA ARS | PRJNA1097562 | SAMN40872828 |  |  |  |
| *Penicillium glabrum* | GCA_037040985.1 | ASM3704098v1 | 69773 | DX136-07W |  |  |  | 34082500 |  | Scaffold | 06/03/2024 | JAXQHR01 | 703988 | 740274 | 436 | Illumina HiSeq | Dalian Medical University | PRJNA833221 | SAMN38094951 |  |  |  |
| *Penicillium glycyrrhizacola* | GCA_025586815.1 | ASM2558681v1 | 1389956 | CGMCC 3.15273 |  |  |  | 30945552 |  | Scaffold | 05/10/2022 | JANFQT01 | 134063 | 194311 | 629 | Illumina HiSeq | Institute of Food Science and Technology, CAAS | PRJNA765789 | SAMN29827634 |  |  |  |
| *Penicillium goetzii* | GCA_037043885.1 | ASM3704388v1 | 1304712 | LH_A469 |  |  |  | 35921107 |  | Contig | 06/03/2024 | JAWLST01 | 386905 |  |  | Illumina | Qingdao Agricultural University | PRJNA1025352 | SAMN37865004 |  |  |  |
| *Penicillium griseofulvum* | GCF_001561935.1 | ASM156193v1 | 5078 | PG3 |  |  | Annotation submitted by University of Turin | 29140916 |  | Contig | 16/02/2016 | LHQR01 | 2267136 |  |  | Illumina MiSeq | University of Turin | PRJNA289974 | SAMN03860337 | 9629 | 9629 |  |
| *Penicillium griseoroseum* | GCA_015586035.1 | ASM1558603v1 | 84562 | IF3SW-F1 |  |  |  | 32029139 |  | Scaffold | 18/11/2020 | JACSPC01 | 475714 | 577257 | 559 | Illumina NovaSeq | Jet Propulsion Laboratory, California Institute of Technology | PRJNA659567 | SAMN15915641 |  |  |  |
| *Penicillium herquei* | GCA_039634405.1 | ASM3963440v1 | 69774 | HGN12.1C (12C) |  |  |  | 34932529 | 8 | Complete Genome | 14/05/2024 |  | 6172464 | 6172464 | 8 | PacBio Sequel | Institute of Biotechnology | PRJNA837748 | SAMN28229866 |  |  |  |
| *Penicillium hetheringtonii* | GCA_028827645.1 | ASM2882764v1 | 911720 | IBT 29057 |  |  | Annotation submitted by Aalborg University | 31629319 |  | Contig | 23/02/2023 | JAQJAC01 | 4061132 |  |  | Oxford Nanopore MinION | Aalborg University | PRJNA867151 | SAMN30185339 | 11892 | 11892 |  |
| *Penicillium hispanicum* | GCF_028827665.1 | ASM2882766v1 | 1080232 | IBT 35686 |  |  | Annotation submitted by Aalborg University | 27868179 |  | Contig | 23/02/2023 | JAQJAD01 | 3706033 |  |  | Oxford Nanopore MinION | Aalborg University | PRJNA867151 | SAMN30185340 | 10111 | 10111 |  |
| *Penicillium hordei* | GCF_028827395.1 | ASM2882739v1 | 40994 | IBT 12815 |  |  | Annotation submitted by Aalborg University | 33792161 |  | Contig | 23/02/2023 | JAQJAE01 | 5075260 |  |  | Oxford Nanopore MinION | Aalborg University | PRJNA867151 | SAMN30185341 | 12337 | 12337 |  |
| *Penicillium italicum* | GCA_002116305.1 | ASM211630v1 | 40296 | GL-Gan1 |  |  |  | 31026583 |  | Scaffold | 01/05/2017 | LWEC01 | 196538 | 316588 | 358 | Illumina HiSeq | BGI | PRJNA317511 | SAMN04620534 |  |  |  |
| *Penicillium janthinellum* | GCA_027569415.1 | ASM2756941v1 | 5079 | NRRL 35451 |  |  |  | 34457699 |  | Contig | 04/01/2023 | JAPDFA01 | 269323 |  |  | Illumina NovaSeq | Northwestern University | PRJNA852164 | SAMN31355061 |  |  |  |
| *Penicillium lagena* | GCF_028827675.1 | ASM2882767v1 | 94218 | IBT 129212 |  |  | Annotation submitted by Aalborg University | 27859125 |  | Contig | 23/02/2023 | JAQJAF01 | 3555488 |  |  | Oxford Nanopore MinION | Aalborg University | PRJNA867151 | SAMN30185342 | 11858 | 11858 |  |
| *Penicillium lividum* | GCA_028828245.1 | ASM2882824v1 | 70099 | IBT 13676 |  |  | Annotation submitted by Aalborg University | 34532832 |  | Contig | 23/02/2023 | JAQJAG01 | 5167816 |  |  | Oxford Nanopore MinION | Aalborg University | PRJNA867151 | SAMN30185344 | 12299 | 12299 |  |
| *Penicillium longicatenatum* | GCF_028827895.1 | ASM2882789v1 | 1561947 | IBT 33135 |  |  | Annotation submitted by Aalborg University | 32457776 |  | Contig | 23/02/2023 | JAQJAH01 | 4747519 |  |  | Oxford Nanopore MinION | Aalborg University | PRJNA867151 | SAMN30185345 | 11980 | 11980 |  |
| *Penicillium maclennaniae* | GCF_028827695.1 | ASM2882769v1 | 1343394 | IBT 15551 |  |  | Annotation submitted by Aalborg University | 27935234 |  | Contig | 23/02/2023 | JAQJAJ01 | 3554904 |  |  | Oxford Nanopore MinION | Aalborg University | PRJNA867151 | SAMN30185347 | 10267 | 10267 |  |
| *Penicillium macrosclerotiorum* | GCF_028827735.1 | ASM2882773v1 | 303699 | IBT 26536 |  |  | Annotation submitted by Aalborg University | 33645934 |  | Contig | 23/02/2023 | JAQJAK01 | 3913342 |  |  | Oxford Nanopore MinION | Aalborg University | PRJNA867151 | SAMN30185348 | 11713 | 11713 |  |
| *Penicillium majusculum* | GCA_028828285.1 | ASM2882828v1 | 2972122 | IBT 35410 |  |  | Annotation submitted by Aalborg University | 33834515 |  | Contig | 23/02/2023 | JAQJAL01 | 9515948 |  |  | Oxford Nanopore MinION | Aalborg University | PRJNA867151 | SAMN30185349 | 12624 | 12624 |  |
| *Penicillium malachiteum* | GCF_028827825.1 | ASM2882782v1 | 1324776 | IBT 17515 |  |  | Annotation submitted by Aalborg University | 35880773 |  | Contig | 23/02/2023 | JAQJAO01 | 8186553 |  |  | Oxford Nanopore MinION | Aalborg University | PRJNA867151 | SAMN30185353 | 13213 | 13212 | 1 |
| *Penicillium manginii* | GCF_028828005.1 | ASM2882800v1 | 203109 | IBT 31320 |  |  | Annotation submitted by Aalborg University | 38623079 |  | Contig | 23/02/2023 | JAQJAP01 | 4165902 |  |  | Oxford Nanopore MinION | Aalborg University | PRJNA867151 | SAMN30185354 | 13795 | 13794 | 1 |
| *Penicillium mononematosum* | GCF_028829835.1 | ASM2882983v1 | 268346 | IBT 11891 |  |  | Annotation submitted by Aalborg University | 30158902 |  | Contig | 23/02/2023 | JAQJZX01 | 8177327 |  |  | Oxford Nanopore MinION | Aalborg University | PRJNA867151 | SAMN30185355 | 11810 | 11810 |  |
| *Penicillium nalgiovense* | GCA_911456355.1 | Pnal_ESE00252 | 60175 |  |  |  | Annotation submitted by University Paris-Sud | 34560068 |  | Contig | 22/09/2022 | CAJVRB01 | 3176469 |  |  |  | University Paris-Sud | PRJEB44534 | SAMEA8618069 | 12159 | 11827 |  |
| *Penicillium nordicum* | GCA_000733025.2 | PnBFE487-1.0 | 229535 | UASWS BFE487 |  |  |  | 30392820 |  | Contig | 10/07/2015 | JNNR01 | 122793 |  |  | Illumina HiSeq | University of Applied Sciences of Western Switzerland | PRJNA239658 | SAMN02666716 |  |  |  |
| *Penicillium nucicola* | GCF_028828085.1 | ASM2882808v1 | 1850975 | IBT 29836 |  |  | Annotation submitted by Aalborg University | 31087567 |  | Contig | 23/02/2023 | JAQJAQ01 | 4788641 |  |  | Oxford Nanopore MinION | Aalborg University | PRJNA867151 | SAMN30185359 | 11519 | 11518 | 1 |
| *Penicillium occitanis (nom. inval.)* | GCA_002382855.1 | ASM238285v1 | 290292 | CL100 |  |  | Annotation submitted by Center for Genomic Regulation (CRG) | 36251488 |  | Contig | 27/09/2017 | NPFK01 | 67126 |  |  | Illumina HiSeq | Center for Genomic Regulation (CRG) | PRJNA377437 | SAMN06461484 | 11231 | 11231 |  |
| *Penicillium ochrochloron* | GCA_022985105.1 | UFV_RLS11_1.0 | 69780 | RLS11 |  |  |  | 38188369 |  | Scaffold | 14/04/2022 | JAAVMA01 | 602663 | 795198 | 724 | Illumina NovaSeq | Universidade Federal de Vicosa | PRJNA614650 | SAMN14438278 |  |  |  |
| *Penicillium odoratum* | GCF_028828045.1 | ASM2882804v1 | 1167516 | IBT 22623 |  |  | Annotation submitted by Aalborg University | 32405987 |  | Contig | 23/02/2023 | JAQJAR01 | 4898923 |  |  | Oxford Nanopore MinION | Aalborg University | PRJNA867151 | SAMN30185360 | 12001 | 12000 | 1 |
| *Penicillium olsonii* | GCA_911174995.1 | Pols_LCP05357 | 99116 |  |  |  | Annotation submitted by University Paris-Sud | 29373279 |  | Scaffold | 22/09/2022 | CAJVNO01 | 740193 | 1148184 | 95 |  | University Paris-Sud | PRJEB44534 | SAMEA8618089 | 9896 | 9538 |  |
| *Penicillium oxalicum* | GCF_001723175.1 | ASM172317v3 | 69781 | HP7-1 |  |  | Annotation submitted by Guangxi University | 30795872 | 8 | Chromosome | 25/04/2022 | JRVD02 | 3811184 | 4068681 | 18 | PacBio Sequel | Guangxi University | PRJNA772803 | SAMN03106237 | 9964 | 9718 |  |
| *Penicillium palitans* | GCA_019190355.1 | ASM1919035v1 | 293373 | F6_7S_1C_F |  |  |  | 36471119 |  | Scaffold | 09/07/2021 | JAHAQQ01 | 211839 | 303786 | 794 | Illumina NovaSeq | Jet Propulsion Laboratory, California Institute of Technology | PRJNA723004 | SAMN18883792 |  |  |  |
| *Penicillium pancosmium* | GCA_031761475.1 | ASM3176147v1 | 1131562 |  |  | MUM 23.27 |  | 34824057 |  | Scaffold | 20/09/2023 | JAVMSB01 | 88330 | 89064 | 766 | Illumina NextSeq | Centre for Functional Ecology - Science for People & the Planet | PRJNA1017377 | SAMN37394757 |  |  |  |
| *Penicillium paneum* | GCA_023627375.1 | ASM2362737v1 | 68879 | M1707 |  |  |  | 27179947 |  | Scaffold | 01/06/2022 | JAMAFK01 | 501797 | 562714 | 256 | Illumina HiSeq | Dalian Medical University | PRJNA833221 | SAMN27963413 |  |  |  |
| *Penicillium paradoxum* | GCF_028828445.1 | ASM2882844v1 | 176176 | IBT 22861 |  |  | Annotation submitted by Aalborg University | 28923777 |  | Contig | 23/02/2023 | JAQJAS01 | 5331884 |  |  | Oxford Nanopore MinION | Aalborg University | PRJNA867151 | SAMN30185361 | 9859 | 9857 | 2 |
| *Penicillium parvum* | GCA_030035635.1 | ASM3003563v1 | 70113 | 4-14b |  |  |  | 25766470 |  | Contig | 18/05/2023 | JAPJNL01 | 3921554 |  |  | PacBio Sequel | Nanjing Forestry University | PRJNA901676 | SAMN31726342 |  |  |  |
| *Penicillium paxilli ATCC 26601* | GCA_000347475.1 | Ppaxilli_v.1.0 | 1292256 | ATCC 26601 |  |  |  | 34802463 |  | Scaffold | 19/03/2013 | AOTG01 | 106755 | 189821 | 420 | Illumina MiSeq | Massey University | PRJNA189173 | SAMN02981508 |  |  |  |
| *Penicillium polonicum* | GCA_034423695.1 | ASM3442369v1 | 60169 | KACC_93368 |  |  |  | 33750493 | 4 | Chromosome | 17/12/2023 |  | 9523896 | 9523896 | 4 | Oxford Nanopore GridION; Illumina NovaSeq | National Institute of Agricultural Sciences | PRJNA1047029 | SAMN38511568 |  |  |  |
| *Penicillium psychrosexuale* | GCF_028828465.1 | ASM2882846v1 | 1002107 | IBT 29551 |  |  | Annotation submitted by Aalborg University | 27827857 |  | Contig | 23/02/2023 | JAQJAT01 | 7382026 |  |  | Oxford Nanopore MinION | Aalborg University | PRJNA867151 | SAMN30185363 | 10487 | 10487 |  |
| *Penicillium pulvis* | GCF_028828015.1 | ASM2882801v1 | 1562058 | IBT 33274 |  |  | Annotation submitted by Aalborg University | 32647626 |  | Contig | 23/02/2023 | JAQJAU01 | 4649848 |  |  | Oxford Nanopore MinION | Aalborg University | PRJNA867151 | SAMN30185364 | 12165 | 12165 |  |
| *Penicillium raperi* | GCA_040333315.1 | ASM4033331v1 | 70100 | F027 |  |  |  | 35587923 |  | Scaffold | 26/06/2024 | JBEBNJ01 | 754312 | 754312 | 285 | BGISEQ | BGI-Qingdao | PRJNA1116794 | SAMN41560904 |  |  |  |
| *Penicillium riverlandense* | GCF_028828495.1 | ASM2882849v1 | 1903569 | IBT 135883 |  |  | Annotation submitted by Aalborg University | 27919989 |  | Contig | 23/02/2023 | JAQJAV01 | 3470598 |  |  | Oxford Nanopore MinION | Aalborg University | PRJNA867151 | SAMN30185365 | 11255 | 11255 |  |
| *Penicillium robsamsonii* | GCF_028829455.1 | ASM2882945v1 | 1792511 | IBT 29466 |  |  | Annotation submitted by Aalborg University | 29159420 |  | Contig | 23/02/2023 | JAQJAW01 | 7219582 |  |  | Oxford Nanopore MinION | Aalborg University | PRJNA867151 | SAMN30185366 | 11254 | 11254 |  |
| *Penicillium rolfsii* | GCA_011392555.1 | CTC_F1880_1.0 | 69785 | F1880 |  |  | Annotation submitted by Embrapa Agroenergia | 32363941 |  | Scaffold | 17/03/2020 | QMFL01 | 944064 | 3147738 | 27 | Illumina Hiseq2000 | Embrapa Agroenergia | PRJNA477323 | SAMN09464826 | 9955 | 9955 |  |
| *Penicillium roqueforti* | GCF_015533775.1 | ASM1553377v1 | 5082 | LCP96 04111 |  |  | Annotation submitted by Utrecht University | 26941230 |  | Scaffold | 17/11/2020 | JABCSE01 | 418688 | 683325 | 83 | Illumina NextSeq500 | Utrecht University | PRJNA627438 | SAMN14669941 | 9810 | 9781 |  |
| *Penicillium rubens* | GCF_028828025.1 | ASM2882802v1 | 1108849 | IBT 27055 |  |  | Annotation submitted by Aalborg University | 30336503 |  | Contig | 23/02/2023 | JAQKAF01 | 9664872 |  |  | Oxford Nanopore MinION | Aalborg University | PRJNA867151 | SAMN30185368 | 11626 | 11626 |  |
| *Penicillium salamii* | GCA_911197225.1 | Psal_DTO309-F9 | 1612424 |  |  |  | Annotation submitted by University Paris-Sud | 31295745 |  | Scaffold | 22/09/2022 | CAJVOY01 | 312816 | 652201 | 164 |  | University Paris-Sud | PRJEB44534 | SAMEA8618106 | 10803 | 10410 |  |
| *Penicillium samsonianum* | GCF_028829775.1 | ASM2882977v1 | 1882272 | IBT 33392 |  |  | Annotation submitted by Aalborg University | 38201884 |  | Contig | 23/02/2023 | JAQJZT01 | 5129283 |  |  | Oxford Nanopore MinION | Aalborg University | PRJNA867151 | SAMN30185370 | 14061 | 14061 |  |
| *Penicillium sclerotigenum* | GCA_928213465.1 | Penicillium_sclerotigenum_IBT15061 | 69787 |  |  |  |  | 29850579 |  | Contig | 09/02/2022 | CAKMRK01 | 101531 |  |  |  | DTU Bioengineering | PRJEB50413 | SAMEA12583987 |  |  |  |
| *Penicillium sclerotiorum* | GCA_911649655.1 | wengan_M_CN111 | 69788 |  |  |  |  | 34772153 |  | Contig | 14/09/2021 | CAJVRJ01 | 4340266 |  |  |  | EGCE | PRJEB46500 | SAMEA8990553 |  |  |  |
| *Penicillium silybi* | GCA_027569585.1 | ASM2756958v1 | 2863086 | G85 |  |  |  | 26976508 |  | Contig | 04/01/2023 | JAPDFI01 | 537536 |  |  | Illumina NovaSeq | Northwestern University | PRJNA852164 | SAMN31355071 |  |  |  |
| *Penicillium simplicissimum* | GCA_024706585.1 | ASM2470658v1 | 69488 | A4 |  |  |  | 38963051 |  | Contig | 17/08/2022 | JAMZTW01 | 3969503 |  |  | PacBio | University of the Western Cape | PRJNA855613 | SAMN29498895 |  |  |  |
| *Penicillium solitum* | GCF_028829755.1 | ASM2882975v1 | 60172 | IBT 25940 |  |  | Annotation submitted by Aalborg University | 33256298 |  | Contig | 23/02/2023 | JAQKAH01 | 4764641 |  |  | Oxford Nanopore MinION | Aalborg University | PRJNA867151 | SAMN30185373 | 12720 | 12720 |  |
| *Penicillium soppii* | GCF_028829465.1 | ASM2882946v1 | 69789 | IBT 18220 |  |  | Annotation submitted by Aalborg University | 32085433 |  | Contig | 23/02/2023 | JAQKAI01 | 3962502 |  |  | Oxford Nanopore MinION | Aalborg University | PRJNA867151 | SAMN30185374 | 12167 | 12167 |  |
| *Penicillium sp. 32 TS-2023* | GCA_039702135.1 | ASM3970213v1 | 3040894 | 32 TS-2023 |  |  |  | 30237888 |  | Contig | 20/05/2024 | JARWGO01 | 75385 |  |  | Oxford Nanopore MinION | Wroclaw University of Environmental and Life Sciences | PRJNA953954 | SAMN34128560 |  |  |  |
| *Penicillium sp. 91 TS-2023* | GCA_030710325.1 | ASM3071032v1 | 3040895 | 91 TS-2023 |  |  |  | 35300634 |  | Contig | 11/08/2023 | JARWGP01 | 5291131 |  |  | Oxford Nanopore MinION | Wroclaw University of Environmental and Life Sciences | PRJNA953954 | SAMN34128561 |  |  |  |
| *Penicillium sp. B98-03* | GCA_037042535.1 | ASM3704253v1 | 3092686 | B98-03 |  |  |  | 28184015 |  | Scaffold | 06/03/2024 | JAXQKP01 | 3558 | 3558 | 10791 | Illumina HiSeq | Dalian Medical University | PRJNA833221 | SAMN38094941 |  |  |  |
| *Penicillium sp. BM32* | GCA_029142745.1 | NB24_v1 | 3027964 | BM32 |  |  |  | 28722057 |  | Contig | 10/03/2023 | JARFLR01 | 4507644 |  |  | Oxford Nanopore GridION | Universite Laval | PRJNA932827 | SAMN33213502 |  |  |  |
| *Penicillium sp. BW_12* | GCA_008931925.1 | ASM893192v1 | 1931375 | BW_12 |  |  |  | 35605011 |  | Scaffold | 09/10/2019 | MUGI01 | 104988 | 132049 | 2019 | Illumina HiSeq | Tufts University | PRJNA354965 | SAMN06054419 |  |  |  |
| *Penicillium sp. BW_162_3FA* | GCA_008931945.1 | ASM893194v1 | 1931376 | BW_162_3FA |  |  |  | 36367533 |  | Scaffold | 09/10/2019 | MUGJ01 | 131027 | 180341 | 1741 | Illumina HiSeq | Tufts University | PRJNA354965 | SAMN06054420 |  |  |  |
| *Penicillium sp. BW_MB* | GCA_008931935.1 | ASM893193v1 | 1931374 | BW_MB |  |  |  | 31635141 |  | Scaffold | 09/10/2019 | MUGH01 | 130753 | 185725 | 905 | Illumina HiSeq | Tufts University | PRJNA354965 | SAMN06054418 |  |  |  |
| *Penicillium sp. C7(2024)* | GCA_037356175.1 | NIH_C7_1.1 | 3115218 | C7(2024) |  |  |  | 32299038 |  | Contig | 19/03/2024 | JAYXSD01 | 2387517 |  |  | Oxford Nanopore MinION; Illumina NextSeq | NLM/NCBI | PRJNA767328 | SAMN39472515 |  |  |  |
| *Penicillium sp. CF01* | GCA_005250745.2 | ASM525074v2 | 2066501 | CF01 |  |  |  | 29669991 |  | Scaffold | 30/11/2022 | RFFF02 | 122551 | 2351384 | 38 | Illumina | INAIL-Research Area | PRJNA427105 | SAMN08223156 |  |  |  |
| *Penicillium sp. CF05* | GCA_002916455.1 | ASM291645v1 | 2066127 | CF05 |  |  |  | 34938837 |  | Scaffold | 05/02/2018 | PKQL01 | 39227 | 39262 | 2289 | Illumina MiSeq | INAIL-Research Area | PRJNA427201 | SAMN08225489 |  |  |  |
| *Penicillium sp. CMV-2018d* | GCA_028827225.1 | ASM2882722v1 | 2184411 | IBT 12396 |  |  | Annotation submitted by Aalborg University | 32244276 |  | Contig | 23/02/2023 | JAPZBW01 | 6566590 |  |  | Oxford Nanopore MinION | Aalborg University | PRJNA867151 | SAMN30185338 | 12024 | 12024 |  |
| *Penicillium sp. D2Mb* | GCA_025768115.1 | ASM2576811v1 | 2940345 | R2202 |  |  |  | 30505107 |  | Scaffold | 20/10/2022 | JAMFOT01 | 172538 | 485078 | 287 | Illumina HiSeq | Dalian Medical University | PRJNA833221 | SAMN27963482 |  |  |  |
| *Penicillium sp. D9* | GCA_037356155.1 | NIH_D9_1.1 | 3115219 | D9 |  |  |  | 35073033 |  | Contig | 19/03/2024 | JAYXSE01 | 3056891 |  |  | Oxford Nanopore MinION; Illumina NextSeq | NLM/NCBI | PRJNA767328 | SAMN39472516 |  |  |  |
| *Penicillium sp. DT28* | GCA_036712075.1 | ASM3671207v1 | 3044587 | DT28 |  |  |  | 23419689 |  | Scaffold | 20/02/2024 | JAZHLO01 | 2619040 | 3945298 | 9 | Illumina MiSeq | Institut hospitalo-universitaire Mediterranee infection | PRJNA967511 | SAMN34598658 |  |  |  |
| *Penicillium sp. DV-2018c* | GCA_028827535.1 | ASM2882753v1 | 2086405 | IBT 19332 |  |  | Annotation submitted by Aalborg University | 25359523 |  | Contig | 23/02/2023 | JAQJAA01 | 4658598 |  |  | Oxford Nanopore MinION | Aalborg University | PRJNA867151 | SAMN30185334 | 9637 | 9636 | 1 |
| *Penicillium sp. E22* | GCA_035048945.1 | UMS_E22 | 3045934 | E22 |  |  |  | 37475709 |  | Scaffold | 03/01/2024 | JASJUN01 | 53337 | 53495 | 2688 | Illumina MiSeq | Universidad de Antofagasta | PRJNA970415 | SAMN35003752 |  |  |  |
| *Penicillium sp. F2(2024)* | GCA_037414295.1 | NIH_F2_1.1 | 3115217 | F2(2024) |  |  |  | 32922503 |  | Contig | 19/03/2024 | JAYXSC01 | 557274 |  |  | Oxford Nanopore MinION; Illumina NextSeq | NLM/NCBI | PRJNA767328 | SAMN39472514 |  |  |  |
| *Penicillium sp. F50* | GCA_037356195.1 | NIH_F50_1.1 | 3115215 | F50 |  |  |  | 33024260 |  | Scaffold | 19/03/2024 | JAYXSA01 | 2686751 | 2686751 | 83 | Oxford Nanopore MinION; Illumina NextSeq | NLM/NCBI | PRJNA767328 | SAMN39472512 |  |  |  |
| *Penicillium sp. F51* | GCA_037356495.1 | NIH_F51_1.1 | 3115216 | F51 |  |  |  | 32984314 |  | Contig | 19/03/2024 | JAYXSB01 | 2762151 |  |  | Oxford Nanopore MinION; Illumina NextSeq | NLM/NCBI | PRJNA767328 | SAMN39472513 |  |  |  |
| *Penicillium sp. G339* | GCA_027569595.1 | ASM2756959v1 | 1569994 | G339 |  |  |  | 32620809 |  | Contig | 04/01/2023 | JAPDFJ01 | 705762 |  |  | Illumina NovaSeq | Northwestern University | PRJNA852164 | SAMN31355074 |  |  |  |
| *Penicillium sp. G342* | GCA_027569895.1 | ASM2756989v1 | 1569995 | G342 |  |  |  | 26531990 |  | Contig | 04/01/2023 | JAPDLA01 | 526838 |  |  | Illumina NovaSeq | Northwestern University | PRJNA852164 | SAMN31355076 |  |  |  |
| *Penicillium sp. HKF2* | GCA_002000375.1 | ASM200037v1 | 887910 |  |  | HKF2 |  | 31484772 |  | Scaffold | 15/02/2017 | MUXA01 | 10874 | 3449782 | 94 | Illumina HiSeq 2500 | CSIR-NEERI | PRJNA371405 | SAMN06298664 |  |  |  |
| *Penicillium sp. IBT 16267x* | GCA_028828865.1 | ASM2882886v1 | 3003415 | IBT 16267x |  |  | Annotation submitted by Aalborg University | 33465021 |  | Contig | 23/02/2023 | JAQJZQ01 | 5071699 |  |  | Oxford Nanopore MinION | Aalborg University | PRJNA867151 | SAMN30185343 | 11479 | 11477 | 2 |
| *Penicillium sp. IBT 18751x* | GCA_028828935.1 | ASM2882893v1 | 3003417 | IBT 18751x |  |  | Annotation submitted by Aalborg University | 30736580 |  | Contig | 23/02/2023 | JAQJZS01 | 3327370 |  |  | Oxford Nanopore MinION | Aalborg University | PRJNA867151 | SAMN30185367 | 11165 | 11164 | 1 |
| *Penicillium sp. IBT 31633x* | GCA_028827885.1 | ASM2882788v1 | 3003414 | IBT 31633x |  |  | Annotation submitted by Aalborg University | 30347723 |  | Contig | 23/02/2023 | JAPWDP01 | 4421608 |  |  | Oxford Nanopore MinION | Aalborg University | PRJNA867151 | SAMN30185300 | 10603 | 10603 |  |
| *Penicillium sp. IBT 35674x* | GCA_028828895.1 | ASM2882889v1 | 3003416 | IBT 35674x |  |  | Annotation submitted by Aalborg University | 37375558 |  | Contig | 23/02/2023 | JAQKAT01 | 4253496 |  |  | Oxford Nanopore MinION | Aalborg University | PRJNA867151 | SAMN30185362 | 12925 | 12925 |  |
| *Penicillium sp. M121-12C* | GCA_037042505.1 | ASM3704250v1 | 3092703 | M121-12C |  |  |  | 31126928 |  | Scaffold | 06/03/2024 | JAXQKM01 | 6312 | 6316 | 8372 | Illumina HiSeq | Dalian Medical University | PRJNA833221 | SAMN38094993 |  |  |  |
| *Penicillium sp. MA 6036* | GCA_003138045.1 | ASM313804v1 | 2153245 | MA 6036 |  |  |  | 33375507 |  | Contig | 18/05/2018 | QAGG01 | 163222 |  |  | IonTorrent | University of Natural Resources and Life Sciences, Vienna | PRJNA432315 | SAMN08819843 |  |  |  |
| *Penicillium sp. MA 6040* | GCA_003138025.1 | ASM313802v1 | 2153247 | MA 6040 |  |  |  | 32104534 |  | Contig | 18/05/2018 | QAGI01 | 123523 |  |  | IonTorrent | University of Natural Resources and Life Sciences, Vienna | PRJNA432315 | SAMN08819845 |  |  |  |
| *Penicillium sp. MBC 424* | GCA_030779445.1 | ASM3077944v1 | 2993620 | MBC 424 |  |  |  | 28473004 |  | Contig | 17/08/2023 | JAPFSX01 | 370403 |  |  | Illumina NovaSeq | USDA-ARS-NCAUR | PRJNA879330 | SAMN31590320 |  |  |  |
| *Penicillium sp. MBC 428* | GCA_030779285.1 | ASM3077928v1 | 2993621 | MBC 428 |  |  |  | 28053325 |  | Contig | 17/08/2023 | JAPFSY01 | 260194 |  |  | Illumina NovaSeq | USDA-ARS-NCAUR | PRJNA879330 | SAMN31590321 |  |  |  |
| *Penicillium sp. MT2 MMC-2018* | GCA_003852855.1 | ASM385285v1 | 2138087 | MT2 MMC-2018 |  |  |  | 33839364 |  | Contig | 30/11/2018 | PZKB01 | 188525 |  |  | Illumina NextSeq | Florida A&M University | PRJNA445729 | SAMN08797514 |  |  |  |
| *Penicillium sp. MT45* | GCA_029142755.1 | NB21_v1 | 3027962 | MT45 |  |  |  | 28661144 |  | Contig | 10/03/2023 | JARFLT01 | 4507709 |  |  | Oxford Nanopore GridION | Universite Laval | PRJNA932827 | SAMN33213500 |  |  |  |
| *Penicillium sp. MYA5* | GCA_037074905.1 | ASM3707490v1 | 3113205 | MYA5 |  |  |  | 32800485 |  | Contig | 08/03/2024 | JAYRCP01 | 2374055 |  |  | Illumina | PLA Naval Medical University | PRJNA1063192 | SAMN39289853 |  |  |  |
| *Penicillium sp. OUCMDZ-019* | GCA_011750695.1 | ASM1175069v1 | 2687245 | OUCMDZ-019 |  |  |  | 31451881 |  | Scaffold | 25/03/2020 | WTTZ01 | 235013 | 267027 | 580 | Illumina HiSeq | ocean university of China | PRJNA595811 | SAMN13567729 |  |  |  |
| *Penicillium sp. PF2412A* | GCA_023626555.1 | ASM2362655v1 | 2940348 | R1202D |  |  |  | 31141126 |  | Scaffold | 01/06/2022 | JAMAHA01 | 167302 | 311384 | 570 | Illumina HiSeq | Dalian Medical University | PRJNA833221 | SAMN27963485 |  |  |  |
| *Penicillium sp. PG106-07D* | GCA_037042255.1 | ASM3704225v1 | 3092724 | PG106-07D |  |  |  | 32269330 |  | Scaffold | 06/03/2024 | JAXQJV01 | 16905 | 16905 | 3418 | Illumina HiSeq | Dalian Medical University | PRJNA833221 | SAMN38095035 |  |  |  |
| *Penicillium sp. PG113-03A* | GCA_037042135.1 | ASM3704213v1 | 3092725 | PG113-03A |  |  |  | 22673652 |  | Scaffold | 06/03/2024 | JAXQJW01 | 357165 | 357165 | 167 | Illumina HiSeq | Dalian Medical University | PRJNA833221 | SAMN38095036 |  |  |  |
| *Penicillium sp. PG115-01* | GCA_037042625.1 | ASM3704262v1 | 3092726 | PG115-01 |  |  |  | 31322363 |  | Scaffold | 06/03/2024 | JAXQKS01 | 128830 | 129580 | 1618 | Illumina HiSeq | Dalian Medical University | PRJNA833221 | SAMN38095037 |  |  |  |
| *Penicillium sp. PH3801* | GCA_023626615.1 | ASM2362661v1 | 2940343 | S1316 |  |  |  | 30790414 |  | Scaffold | 01/06/2022 | JAMAHE01 | 105815 | 139466 | 720 | Illumina HiSeq | Dalian Medical University | PRJNA833221 | SAMN27963480 |  |  |  |
| *Penicillium sp. PT4103* | GCA_023626495.1 | ASM2362649v1 | 2940347 | D2Mb |  |  |  | 29949327 |  | Scaffold | 01/06/2022 | JAMAHB01 | 231638 | 421598 | 401 | Illumina HiSeq | Dalian Medical University | PRJNA833221 | SAMN27963484 |  |  |  |
| *Penicillium sp. R1202D* | GCA_023626515.1 | ASM2362651v1 | 2940346 | S1126A |  |  |  | 30951873 |  | Scaffold | 01/06/2022 | JAMAHC01 | 177303 | 497993 | 364 | Illumina HiSeq | Dalian Medical University | PRJNA833221 | SAMN27963483 |  |  |  |
| *Penicillium sp. R2202* | GCA_023625675.1 | ASM2362567v1 | 2940389 | 2NP930A |  |  |  | 10640586 |  | Scaffold | 01/06/2022 | JAMAFL01 | 550810 | 743676 | 23 | Illumina HiSeq | Dalian Medical University | PRJNA833221 | SAMN27963590 |  |  |  |
| *Penicillium sp. S103-07* | GCA_037042055.1 | ASM3704205v1 | 3092740 | S103-07 |  |  |  | 29774733 |  | Scaffold | 06/03/2024 | JAXQJQ01 | 330500 | 409534 | 516 | Illumina HiSeq | Dalian Medical University | PRJNA833221 | SAMN38095080 |  |  |  |
| *Penicillium sp. S1126A* | GCA_023626535.1 | ASM2362653v1 | 2940344 | PH3801 |  |  |  | 30421393 |  | Scaffold | 01/06/2022 | JAMAHD01 | 204709 | 503016 | 433 | Illumina HiSeq | Dalian Medical University | PRJNA833221 | SAMN27963481 |  |  |  |
| *Penicillium sp. S1316* | GCA_023627405.1 | ASM2362740v1 | 2940342 | R2504 |  |  |  | 41245004 |  | Scaffold | 01/06/2022 | JAMAHF01 | 318432 | 391880 | 701 | Illumina HiSeq | Dalian Medical University | PRJNA833221 | SAMN27963380 |  |  |  |
| *Penicillium sp. SPG-F1* | GCA_003800495.2 | ASM380049v2 | 2488753 | SPG-F1 |  |  |  | 32629321 |  | Contig | 26/04/2023 | PUXE02 | 1348856 |  |  | Illumina HiSeq; PacBio RSII | Texas A&M University Corpus Christi | PRJNA435890 | SAMN08606007 |  |  |  |
| *Penicillium sp. SPG-F15* | GCA_003800485.2 | ASM380048v2 | 2488754 | SPG-F15 |  |  |  | 36414411 |  | Contig | 26/04/2023 | PUHX02 | 3205559 |  |  | Illumina HiSeq; PacBio RSII | Texas A&M University - Corpus Christi | PRJNA435885 | SAMN08605975 |  |  |  |
| *Penicillium sp. str. #12* | GCA_013138035.1 | ASM1313803v1 | 2720512 | #12 |  |  | Annotation submitted by None | 37990757 |  | Contig | 21/05/2020 | JAASRZ01 | 2900369 |  |  | Oxford Nanopore MinION; Illumina | None | PRJNA612335 | SAMN14369290 | 13258 | 13098 | 160 |
| *Penicillium sp. VS AB III KN* | GCA_019775275.1 | ASM1977527v1 | 2756251 | VS AB III KN |  |  |  | 24864245 |  | Scaffold | 26/08/2021 | JACWGB01 | 420200 | 597985 | 296 | Illumina | Jet Propulsion Laboratory, California Institute of Technology | PRJNA644637 | SAMN15543691 |  |  |  |
| *Penicillium sp. VS AB III KN 1* | GCA_019775305.1 | ASM1977530v1 | 2756252 | VS AB III KN 1 |  |  |  | 24854136 |  | Scaffold | 26/08/2021 | JACWGC01 | 402187 | 606931 | 312 | Illumina | Jet Propulsion Laboratory, California Institute of Technology | PRJNA644637 | SAMN15543692 |  |  |  |
| *Penicillium sp. VS I D KN* | GCA_019775415.1 | ASM1977541v1 | 2756250 | VS I D KN |  |  |  | 35174464 |  | Scaffold | 26/08/2021 | JACWFT01 | 488658 | 995446 | 432 | Illumina | Jet Propulsion Laboratory, California Institute of Technology | PRJNA644637 | SAMN15543677 |  |  |  |
| *Penicillium sp. VS I D KN 3* | GCA_019775465.1 | ASM1977546v1 | 2756249 | VS I D KN 3 |  |  |  | 35127538 |  | Scaffold | 26/08/2021 | JACWFS01 | 651115 | 870778 | 452 | Illumina | Jet Propulsion Laboratory, California Institute of Technology | PRJNA644637 | SAMN15543675 |  |  |  |
| *Penicillium sp. WT45* | GCA_029142805.1 | NB22_v1 | 3027963 | WT45 |  |  |  | 28758677 |  | Contig | 10/03/2023 | JARFLS01 | 4507813 |  |  | Oxford Nanopore GridION | Universite Laval | PRJNA932827 | SAMN33213501 |  |  |  |
| *Penicillium steckii* | GCA_018340795.1 | PS_2648 | 303698 | P2648 |  |  |  | 33404627 |  | Scaffold | 11/05/2021 | JADDUG01 | 225211 | 1400824 | 952 | Illumina HiSeq | Minjiang University | PRJNA671565 | SAMN16534233 |  |  |  |
| *Penicillium subrubescens* | GCF_028828155.1 | ASM2882815v1 | 1316194 | IBT 31985 |  |  | Annotation submitted by Aalborg University | 39968067 |  | Contig | 23/02/2023 | JAQKAJ01 | 4156290 |  |  | Oxford Nanopore MinION | Aalborg University | PRJNA867151 | SAMN30185375 | 13458 | 13458 |  |
| *Penicillium sumatraense* | GCA_020086695.1 | ASM2008669v1 | 70558 | AQ67100 |  |  |  | 36393065 |  | Scaffold | 21/09/2021 | JAGIKU01 | 923193 | 1455188 | 4510 | Illumina NovaSeq | University of L'Aquila | PRJNA716633 | SAMN18441718 |  |  |  |
| *Penicillium tannophilum* | GCA_028829675.1 | ASM2882967v1 | 2972123 | IBT 21756 |  |  | Annotation submitted by Aalborg University | 32107691 |  | Contig | 23/02/2023 | JAQKAK01 | 4205002 |  |  | Oxford Nanopore MinION | Aalborg University | PRJNA867151 | SAMN30185377 | 12216 | 12214 | 2 |
| *Penicillium taxi* | GCF_028828555.1 | ASM2882855v1 | 168475 | IBT 34144 |  |  | Annotation submitted by Aalborg University | 27925195 |  | Contig | 23/02/2023 | JAQKAL01 | 5061136 |  |  | Oxford Nanopore MinION | Aalborg University | PRJNA867151 | SAMN30185378 | 10052 | 10052 |  |
| *Penicillium thymicola* | GCA_030142185.1 | ASM3014218v1 | 293382 | DAOM 180753 |  |  | Annotation submitted by Agriculture and Agri-Food Canada | 33877935 |  | Scaffold | 31/05/2023 | LACB01 | 44290 | 45351 | 2167 | Illumina HiSeq | Agriculture and Agri-Food Canada | PRJNA277835 | SAMN03397375 | 12921 | 12921 |  |
| *Penicillium turbatum* | GCA_030762825.1 | ASM3076282v1 | 70105 | BLH34 |  |  |  | 28026804 |  | Contig | 16/08/2023 | JAUJYX01 | 2706266 |  |  | Oxford Nanopore MinION; Illumina NextSeq | West Anhui University | PRJNA992653 | SAMN36368430 |  |  |  |
| *Penicillium ucsense* | GCA_014839625.1 | UCS_PECM_1.0 | 2839758 | S1M29 |  |  | Annotation submitted by Universidade de Caxias do Sul | 30406053 |  | Scaffold | 02/10/2020 | WIWV01 | 161556 | 185175 | 673 | Illumina HiSeq | Universidade de Caxias do Sul | PRJNA521489 | SAMN10889014 | 8375 | 8173 |  |
| *Penicillium verhagenii* | GCF_028828195.1 | ASM2882819v1 | 1562060 | IBT 33310 |  |  | Annotation submitted by Aalborg University | 32288599 |  | Contig | 23/02/2023 | JAQKAM01 | 3526632 |  |  | Oxford Nanopore MinION | Aalborg University | PRJNA867151 | SAMN30185379 | 11673 | 11673 |  |
| *Penicillium verrucosum* | GCF_028828655.1 | ASM2882865v1 | 60171 | IBT 35672 |  |  | Annotation submitted by Aalborg University | 32831822 |  | Contig | 23/02/2023 | JAQKAO01 | 5850290 |  |  | Oxford Nanopore MinION | Aalborg University | PRJNA867151 | SAMN30185381 | 11555 | 11555 |  |
| *Penicillium viridicatum* | GCA_028828185.1 | ASM2882818v1 | 60134 | IBT 34249 |  |  | Annotation submitted by Aalborg University | 32834375 |  | Contig | 23/02/2023 | JAQKAP01 | 8985949 |  |  | Oxford Nanopore MinION | Aalborg University | PRJNA867151 | SAMN30185382 | 12237 | 12237 |  |
| *Penicillium vulpinum* | GCF_028829585.1 | ASM2882958v1 | 29845 | IBT 29486 |  |  | Annotation submitted by Aalborg University | 31511571 |  | Contig | 23/02/2023 | JAQKAQ01 | 8559827 |  |  | Oxford Nanopore MinION | Aalborg University | PRJNA867151 | SAMN30185383 | 11521 | 11521 |  |
| *Penicillium waksmanii* | GCF_028829765.1 | ASM2882976v1 | 69791 | IBT 27052 |  |  | Annotation submitted by Aalborg University | 37575496 |  | Contig | 23/02/2023 | JAQKAR01 | 4855768 |  |  | Oxford Nanopore MinION | Aalborg University | PRJNA867151 | SAMN30185384 | 13502 | 13502 |  |
| *Pseudotulostoma volvatum* | GCA_019804575.2 | ASM1980457v2 | 144541 | 6Q3QHG6JMA |  |  |  | 57179449 |  | Scaffold | 28/12/2022 | JAHQZX02 | 32298 | 33127 | 5628 | Illumina | Iridian Genomes | PRJNA328806 | SAMN05382276 |  |  |  |
| *Rasamsonia emersonii CBS 393.64* | GCF_000968595.1 | ASM96859v1 | 1408163 | CBS 393.64 |  |  | Annotation submitted by NCBI RefSeq | 28248805 |  | Contig | 03/04/2015 | LASV01 | 65650 |  |  | Sanger 3730XL; 454 GS-FLX | DSM Bio-based Products & Services B.V. | PRJNA222976 | SAMN03453569 | 9841 | 9841 |  |
| *Talaromyces adpressus* | GCA_002775195.1 | ASM277519v1 | 1974180 | CBS 142503 |  |  |  | 36107696 |  | Scaffold | 16/11/2017 | NHZS01 | 125648 | 187230 | 585 | Illumina HiSeq | University of Naples "Federico II" | PRJNA381192 | SAMN06671952 |  |  |  |
| *Talaromyces albobiverticillius* | GCA_023721895.2 | ASM2372189v2 | 1441468 | Tp-2 |  |  |  | 38354882 |  | Contig | 16/11/2022 | JAMBUR02 | 4594200 |  |  | Oxford Nanopore | Nanyang Normal University | PRJNA835810 | SAMN28111088 |  |  |  |
| *Talaromyces amestolkiae* | GCF_001896365.1 | ASM189636v1 | 1196081 | CIB |  |  | Annotation submitted by Centro de Investigaciones Biologicas | 33721883 |  | Scaffold | 09/12/2016 | MIKG01 | 936823 | 1486010 | 212 | Illumina | Centro de Investigaciones Biologicas | PRJNA342263 | SAMN05751675 | 10404 | 10403 | 1 |
| *Talaromyces atroroseus* | GCF_001907595.1 | ASM190759v1 | 1441469 | IBT 11181 |  |  | Annotation submitted by Technical University of Denmark | 30858562 |  | Scaffold | 16/12/2016 | LFMY01 | 376103 | 1577698 | 48 | Illumina HiSeq | Technical University of Denmark | PRJNA275056 | SAMN03339010 | 9523 | 9523 |  |
| *Talaromyces borbonicus* | GCA_002916415.1 | ASM291641v1 | 2077152 | SV-2017a |  |  |  | 27050413 |  | Scaffold | 05/02/2018 | NBSA01 | 521985 | 2199342 | 28 | Illumina HiSeq | University of Naples "Federico II" | PRJNA379116 | SAMN06579453 |  |  |  |
| *Talaromyces funiculosus* | GCA_004299765.1 | ASM429976v1 | 28572 | X33 |  |  |  | 28488493 | 21 | Complete Genome | 26/02/2019 |  | 2117970 | 2117970 | 21 | PacBio | Shandong Agricultural University | PRJNA508439 | SAMN10522600 |  |  |  |
| *Talaromyces fuscoviridis* | GCA_040333115.1 | ASM4033311v1 | 1702300 | F034 |  |  |  | 36102204 |  | Scaffold | 26/06/2024 | JBEBNF01 | 112740 | 112740 | 1977 | BGISEQ | BGI-Qingdao | PRJNA1116794 | SAMN41560908 |  |  |  |
| *Talaromyces islandicus* | GCA_000985935.1 | PIS | 28573 |  |  | WF-38-12 | Annotation submitted by CEBITEC | 34715840 |  | Scaffold | 29/04/2015 | CVMT01 | 63871 | 2353708 | 116 |  | CEBITEC | PRJEB8788 | SAMEA3344808 | 10094 | 9927 |  |
| *Talaromyces liani* | GCA_022814505.1 | ASM2281450v1 | 1295094 | FKII-L2-CM-DRAB3 |  |  |  | 35499968 |  | Scaffold | 04/04/2022 | JAKLNL01 | 436959 | 735697 | 385 | Illumina NovaSeq | Jet Propulsion Laboratory, California Institute of Technology | PRJNA800051 | SAMN25226818 |  |  |  |
| *Talaromyces marneffei* | GCF_009556855.1 | ASM955685v1 | 37727 |  |  | 11CN-20-091 | Annotation submitted by Broad Institute | 28198338 | 8 | Complete Genome | 05/11/2019 |  | 3704010 | 3704010 | 8 | Oxford Nanopore MiniION | Broad Institute | PRJNA522919 | SAMN10960796 | 10066 | 9994 | 22 |
| *Talaromyces nanjingensis* | GCA_031010415.1 | ASM3101041v1 | 2916467 | JP-NJ4 |  |  |  | 42526372 |  | Contig | 30/08/2023 | JARFPJ01 | 5083175 |  |  | Illumina NovaSeq 6000; DNBSEQ-T7; Oxford Nanopore | Nanjing Forestry University | PRJNA941179 | SAMN33591157 |  |  |  |
| *Talaromyces piceae* | GCA_001657655.1 | ASM165765v1 | 153982 | 09-mars |  |  |  | 26564319 |  | Scaffold | 03/06/2016 | JNNX01 | 997367 | 2854447 | 12 | Illumina HiSeq | Tianjin Institute of Biotechnology, CAS | PRJNA251507 | SAMN02834830 |  |  |  |
| *Talaromyces pinophilus* | GCA_001571465.2 | ASM157146v2 | 128442 | janv-95 |  |  |  | 36480443 | 8 | Complete Genome | 18/07/2017 |  | 4804168 | 4804168 | 8 | PacBio; Illumina HiSeq | Beijing Genomics Institute | PRJNA310372 | SAMN04451644 |  |  |  |
| *Talaromyces proteolyticus* | GCF_021365285.1 | Talpro1 | 1131652 | PMI_201 |  |  | Annotation submitted by DOE Joint Genome Institute | 37611490 |  | Contig | 05/01/2022 | JAJTJA01 | 2609059 |  |  | PacBio | DOE Joint Genome Institute | PRJNA340474 | SAMN05720779 | 13665 | 13585 |  |
| *Talaromyces purpureogenus* | GCA_019022425.1 | ASM1902242v1 | 1266744 | Q2 |  |  |  | 29077759 |  | Contig | 24/06/2021 | JABUIS01 | 1919420 |  |  | Illumina MiSeq; PacBio | Shandong Agricultural University | PRJNA635544 | SAMN15042938 |  |  |  |
| *Talaromyces ruber* | GCA_022813215.1 | ASM2281321v1 | 1266769 | FKI-L3-BK-DAB3 |  |  |  | 30034193 |  | Scaffold | 04/04/2022 | JAKLMW01 | 403914 | 473555 | 346 | Illumina NovaSeq | Jet Propulsion Laboratory, California Institute of Technology | PRJNA800051 | SAMN25226803 |  |  |  |
| *Talaromyces rugulosus* | GCF_013368755.1 | ASM1336875v1 | 121627 | W13939 |  |  | Annotation submitted by Xi'an Jiaotong University | 35758560 | 6 | Complete Genome | 23/06/2020 |  | 5896210 | 5896210 | 6 | PacBio RSII | Xi'an Jiaotong University | PRJNA639236 | SAMN15229550 | 11971 | 11901 |  |
| *Talaromyces sp. B14P1* | GCA_023626195.1 | ASM2362619v1 | 2940361 | PYS2902 |  |  |  | 34461311 |  | Scaffold | 01/06/2022 | JAMAGN01 | 816354 | 816354 | 294 | Illumina HiSeq | Dalian Medical University | PRJNA833221 | SAMN27963520 |  |  |  |
| *Talaromyces sp. DC2* | GCA_039119965.1 | ASM3911996v1 | 3142527 | DC2 |  |  |  | 34575287 |  | Contig | 02/05/2024 | JBCLNQ01 | 346458 |  |  | PacBio Sequel | Institute of Genome Research | PRJNA1002839 | SAMN36864296 |  |  |  |
| *Talaromyces sp. F31* | GCA_037356135.1 | NIH_F31_1.1 | 3115220 | F31 |  |  |  | 34784245 |  | Scaffold | 19/03/2024 | JAYXSF01 | 2597666 | 2606381 | 59 | Oxford Nanopore MinION; Illumina NextSeq | NLM/NCBI | PRJNA767328 | SAMN39472517 |  |  |  |
| *Talaromyces sp. F3606* | GCA_023625895.1 | ASM2362589v1 | 2940375 | PYS2103 |  |  |  | 29457564 |  | Scaffold | 01/06/2022 | JAMAFZ01 | 1302655 | 1942619 | 183 | Illumina HiSeq | Dalian Medical University | PRJNA833221 | SAMN27963534 |  |  |  |
| *Talaromyces sp. P118-02* | GCA_037040535.1 | ASM3704053v1 | 3092716 | P118-02 |  |  |  | 32425225 |  | Scaffold | 06/03/2024 | JAXQGY01 | 411705 | 411705 | 930 | Illumina HiSeq | Dalian Medical University | PRJNA833221 | SAMN38095013 |  |  |  |
| *Talaromyces sp. P2101* | GCA_023626015.1 | ASM2362601v1 | 2940371 | PF2101 |  |  |  | 27704390 |  | Scaffold | 01/06/2022 | JAMAGD01 | 344692 | 417544 | 384 | Illumina HiSeq | Dalian Medical University | PRJNA833221 | SAMN27963530 |  |  |  |
| *Talaromyces sp. PF2101* | GCA_023626055.1 | ASM2362605v1 | 2940369 | X2403 |  |  |  | 33878408 |  | Scaffold | 01/06/2022 | JAMAGF01 | 444093 | 723137 | 192 | Illumina HiSeq | Dalian Medical University | PRJNA833221 | SAMN27963528 |  |  |  |
| *Talaromyces sp. PF30-10* | GCA_023626295.1 | ASM2362629v1 | 2940358 | PF30-1C |  |  |  | 34746589 |  | Scaffold | 01/06/2022 | JAMAGQ01 | 977410 | 1440391 | 398 | Illumina HiSeq | Dalian Medical University | PRJNA833221 | SAMN27963517 |  |  |  |
| *Talaromyces sp. PF30-1C* | GCA_023626275.1 | ASM2362627v1 | 2940356 | PF30-5 |  |  |  | 34714567 |  | Scaffold | 01/06/2022 | JAMAGS01 | 917269 | 1528857 | 355 | Illumina HiSeq | Dalian Medical University | PRJNA833221 | SAMN27963515 |  |  |  |
| *Talaromyces sp. PF30-1D* | GCA_023626315.1 | ASM2362631v1 | 2940357 | PG4302 |  |  |  | 34879448 |  | Scaffold | 01/06/2022 | JAMAGR01 | 615832 | 975773 | 401 | Illumina HiSeq | Dalian Medical University | PRJNA833221 | SAMN27963516 |  |  |  |
| *Talaromyces sp. PF30-5* | GCA_023626375.1 | ASM2362637v1 | 2940354 | S1602 |  |  |  | 35401712 |  | Scaffold | 01/06/2022 | JAMAGU01 | 511630 | 1282550 | 342 | Illumina HiSeq | Dalian Medical University | PRJNA833221 | SAMN27963493 |  |  |  |
| *Talaromyces sp. PF3502* | GCA_023625995.1 | ASM2362599v1 | 2940370 | PM2405 |  |  |  | 34671233 |  | Scaffold | 01/06/2022 | JAMAGE01 | 588108 | 1547124 | 158 | Illumina HiSeq | Dalian Medical University | PRJNA833221 | SAMN27963529 |  |  |  |
| *Talaromyces sp. PG4302* | GCA_023626335.1 | ASM2362633v1 | 2940355 | PT3601 |  |  |  | 34770149 |  | Scaffold | 01/06/2022 | JAMAGT01 | 1282707 | 1548536 | 395 | Illumina HiSeq | Dalian Medical University | PRJNA833221 | SAMN27963514 |  |  |  |
| *Talaromyces sp. PH2104* | GCA_023625955.1 | ASM2362595v1 | 2940374 | PT2101 |  |  |  | 29383702 |  | Scaffold | 01/06/2022 | JAMAGA01 | 1087459 | 1566529 | 187 | Illumina HiSeq | Dalian Medical University | PRJNA833221 | SAMN27963533 |  |  |  |
| *Talaromyces sp. PL2406* | GCA_023626215.1 | ASM2362621v1 | 2940363 | B14P1 |  |  |  | 32900204 |  | Scaffold | 01/06/2022 | JAMAGL01 | 1015881 | 1598135 | 325 | Illumina HiSeq | Dalian Medical University | PRJNA833221 | SAMN27963522 |  |  |  |
| *Talaromyces sp. PM2405* | GCA_023626075.1 | ASM2362607v1 | 2940368 | R2405 |  |  |  | 35331725 |  | Scaffold | 01/06/2022 | JAMAGG01 | 576836 | 1079229 | 330 | Illumina HiSeq | Dalian Medical University | PRJNA833221 | SAMN27963527 |  |  |  |
| *Talaromyces sp. PT2101* | GCA_023626035.1 | ASM2362603v1 | 2940372 | PF3502 |  |  |  | 28879243 |  | Scaffold | 01/06/2022 | JAMAGC01 | 715212 | 1058348 | 508 | Illumina HiSeq | Dalian Medical University | PRJNA833221 | SAMN27963531 |  |  |  |
| *Talaromyces sp. PT3601* | GCA_023626395.1 | ASM2362639v1 | 2940353 | M1327 |  |  |  | 33633690 |  | Scaffold | 01/06/2022 | JAMAGV01 | 561028 | 1667699 | 199 | Illumina HiSeq | Dalian Medical University | PRJNA833221 | SAMN27963492 |  |  |  |
| *Talaromyces sp. PX1902* | GCA_023626175.1 | ASM2362617v1 | 2940362 | PX2802 |  |  |  | 35032352 |  | Scaffold | 01/06/2022 | JAMAGM01 | 689600 | 728479 | 321 | Illumina HiSeq | Dalian Medical University | PRJNA833221 | SAMN27963521 |  |  |  |
| *Talaromyces sp. PX2406* | GCA_023626115.1 | ASM2362611v1 | 2940365 | PL2406 |  |  |  | 34271254 |  | Scaffold | 01/06/2022 | JAMAGJ01 | 574036 | 1506329 | 182 | Illumina HiSeq | Dalian Medical University | PRJNA833221 | SAMN27963524 |  |  |  |
| *Talaromyces sp. PX2802* | GCA_023626255.1 | ASM2362625v1 | 2940360 | PF30-10 |  |  |  | 34594379 |  | Scaffold | 01/06/2022 | JAMAGO01 | 1273708 | 1557128 | 361 | Illumina HiSeq | Dalian Medical University | PRJNA833221 | SAMN27963519 |  |  |  |
| *Talaromyces sp. PYS2103* | GCA_023625975.1 | ASM2362597v1 | 2940373 | P2101 |  |  |  | 29277153 |  | Scaffold | 01/06/2022 | JAMAGB01 | 537766 | 683545 | 543 | Illumina HiSeq | Dalian Medical University | PRJNA833221 | SAMN27963532 |  |  |  |
| *Talaromyces sp. PYS2902* | GCA_023626235.1 | ASM2362623v1 | 2940359 | PF30-1D |  |  |  | 34806200 |  | Scaffold | 01/06/2022 | JAMAGP01 | 1192485 | 1632542 | 467 | Illumina HiSeq | Dalian Medical University | PRJNA833221 | SAMN27963518 |  |  |  |
| *Talaromyces sp. R2405* | GCA_023626155.1 | ASM2362615v1 | 2940366 | R2412 |  |  |  | 34240689 |  | Scaffold | 01/06/2022 | JAMAGI01 | 612250 | 1442526 | 214 | Illumina HiSeq | Dalian Medical University | PRJNA833221 | SAMN27963525 |  |  |  |
| *Talaromyces sp. R2412* | GCA_023626135.1 | ASM2362613v1 | 2940364 | PX1902 |  |  |  | 33681611 |  | Scaffold | 01/06/2022 | JAMAGK01 | 366618 | 721371 | 593 | Illumina HiSeq | Dalian Medical University | PRJNA833221 | SAMN27963523 |  |  |  |
| *Talaromyces sp. SJ9* | GCA_038993655.1 | ASM3899365v1 | 3134886 | SJ9 |  |  |  | 39765480 |  | Contig | 01/05/2024 | JBBVQN01 | 613789 |  |  | Illumina NovaSeq | Qilu University of Technology | PRJNA1090279 | SAMN40561778 |  |  |  |
| *Talaromyces sp. UPCC* | GCA_040669935.1 | ASM4066993v1 | 3161998 | UPCC |  |  |  | 31319356 |  | Contig | 12/07/2024 | JBEPII01 | 1003615 |  |  | Illumina Novaseq 6000 | University of the Philippines Diliman | PRJNA1109622 | SAMN41278738 |  |  |  |
| *Talaromyces sp. X2403* | GCA_023626095.1 | ASM2362609v1 | 2940367 | PX2406 |  |  |  | 35045590 |  | Scaffold | 01/06/2022 | JAMAGH01 | 659013 | 1241449 | 123 | Illumina HiSeq | Dalian Medical University | PRJNA833221 | SAMN27963526 |  |  |  |
| *Talaromyces stipitatus ATCC 10500* | GCF_000003125.1 | JCVI-TSTA1-3.0 | 441959 | ATCC 10500 |  |  | Annotation submitted by J. Craig Venter Institute | 35685443 |  | Scaffold | 22/12/2008 | ABAS01 | 897397 | 4363329 | 820 |  | J. Craig Venter Institute | PRJNA19557 | SAMN02953686 | 12572 | 12455 | 13 |
| *Talaromyces stollii* | GCA_037040825.1 | ASM3704082v1 | 1266822 | P8401 |  |  |  | 34321605 |  | Contig | 06/03/2024 | JAXQHJ01 | 931107 |  |  | Illumina HiSeq | Dalian Medical University | PRJNA833221 | SAMN38095030 |  |  |  |
| *Talaromyces thailandensis* | GCA_019828575.1 | ASM1982857v1 | 1297587 | OC-R06-P5 |  |  |  | 31813847 |  | Scaffold | 31/08/2021 | JACVQZ01 | 228729 | 278008 | 581 | Illumina | Jet Propulsion Laboratory, California Institute of Technology | PRJNA644637 | SAMN15793537 |  |  |  |
| *Talaromyces trachyspermus* | GCA_020137715.1 | BUMICRO_TalaroTrachy_1.1 | 28566 | 4014 |  |  |  | 32038095 |  | Contig | 05/10/2021 | JACGXQ01 | 3814881 |  |  | Oxford Nanopore | Barkatullah University | PRJNA650128 | SAMN15692527 |  |  |  |
| *Talaromyces variabilis* | GCA_040333175.1 | ASM4033317v1 | 28576 | HXQ-H-1 |  |  |  | 33780117 |  | Scaffold | 26/06/2024 | JBEBND01 | 2127226 | 5544665 | 7 | BGISEQ | BGI-Qingdao | PRJNA1116794 | SAMN41560910 |  |  |  |
| *Talaromyces verruculosus* | GCA_001305275.1 | ASM130527v1 | 198730 | TS63-9 |  |  | Annotation submitted by Penicillium verruculosum | 37629238 |  | Scaffold | 01/10/2015 | LHCL01 | 231731 | 353466 | 540 | Illumina HiSeq | Penicillium verruculosum | PRJNA291496 | SAMN03944927 | 11326 | 11326 |  |
| *Talaromyces wortmannii* | GCA_001939245.1 | ASM193924v1 | 28567 | LMB-HP14 |  |  |  | 38588669 |  | Scaffold | 09/01/2017 | MJVA01 | 522133 | 685936 | 1191 | Illumina HiSeq | Universidad Nacional Agraria La Molina, | PRJNA300345 | SAMN04219463 |  |  |  |
| *Thermoascaceae sp. COH1141* | GCA_003123655.1 | ASM312365v1 | 2562446 | COH1141 |  |  |  | 31851014 |  | Scaffold | 14/05/2018 | QBDR01 | 622563 | 1821855 | 40 | Illumina HiSeq | Jet Propulsion Laboratory, California Institute of Technology | PRJNA449516 | SAMN08905905 |  |  |  |
| *Thermoascaceae sp. M2901* | GCA_023624095.1 | ASM2362409v1 | 2940442 | PK2413 |  |  |  | 32052293 |  | Scaffold | 01/06/2022 | JAMADS01 | 1013457 | 1493533 | 34 | Illumina HiSeq | Dalian Medical University | PRJNA833221 | SAMN27963495 |  |  |  |
| *Thermoascaceae sp. M2902* | GCA_023624045.1 | ASM2362404v1 | 2940445 | PL2405 |  |  |  | 31979780 |  | Scaffold | 01/06/2022 | JAMADP01 | 988456 | 1538680 | 147 | Illumina HiSeq | Dalian Medical University | PRJNA833221 | SAMN27963498 |  |  |  |
| *Thermoascaceae sp. PK2413* | GCA_023624055.1 | ASM2362405v1 | 2940444 | M2901 |  |  |  | 32089604 |  | Scaffold | 01/06/2022 | JAMADQ01 | 1086975 | 1951565 | 52 | Illumina HiSeq | Dalian Medical University | PRJNA833221 | SAMN27963497 |  |  |  |
| *Thermoascaceae sp. PL2402* | GCA_023624085.1 | ASM2362408v1 | 2940443 | PL2402 |  |  |  | 32030737 |  | Scaffold | 01/06/2022 | JAMADR01 | 1044076 | 1582779 | 55 | Illumina HiSeq | Dalian Medical University | PRJNA833221 | SAMN27963496 |  |  |  |
| *Thermoascaceae sp. PL2405* | GCA_023624145.1 | ASM2362414v1 | 2940441 | M2902 |  |  |  | 31976459 |  | Scaffold | 01/06/2022 | JAMADT01 | 989569 | 1521794 | 168 | Illumina HiSeq | Dalian Medical University | PRJNA833221 | SAMN27963494 |  |  |  |
| *Thermoascus crustaceus* | GCA_001599835.1 | JCM_12817_assembly_v001 | 5088 | JCM 12817 |  |  |  | 31644316 |  | Scaffold | 01/03/2016 | BCIC01 | 290402 | 3953819 | 33 | HiSeq 2500 | RIKEN Center for Life Science Technologies, Division of Genomic Technologies | PRJDB3664 | SAMD00028384 |  |  |  |
| *Thermomyces lanuginosus SSBP* | GCA_000315935.1 | NOKS_genome_assembly_20110720 | 1158138 | SSBP |  |  |  | 19155516 |  | Contig | 29/11/2012 | ANHP01 | 113553 |  |  | 454; Illumina GAIIx | Durban University of Technology | PRJNA88097 | SAMN02981479 |  |  |  |
| *Trichocomaceae sp. PB3601* | GCA_023625835.1 | ASM2362583v1 | 2940378 | PB3603 |  |  |  | 34074703 |  | Scaffold | 01/06/2022 | JAMAFW01 | 988055 | 1347693 | 367 | Illumina HiSeq | Dalian Medical University | PRJNA833221 | SAMN27963537 |  |  |  |
| *Trichocomaceae sp. PB3603* | GCA_023625915.1 | ASM2362591v1 | 2940376 | PH2104 |  |  |  | 29540058 |  | Scaffold | 01/06/2022 | JAMAFY01 | 1118191 | 1403909 | 192 | Illumina HiSeq | Dalian Medical University | PRJNA833221 | SAMN27963535 |  |  |  |
| *Trichocomaceae sp. PYS4502* | GCA_023625875.1 | ASM2362587v1 | 2940377 | F3606 |  |  |  | 29264059 |  | Scaffold | 01/06/2022 | JAMAFX01 | 86989 | 91680 | 896 | Illumina HiSeq | Dalian Medical University | PRJNA833221 | SAMN27963536 |  |  |  |
| *Xeromyces bisporus* | GCA_900006255.1 | Xbisp_v1.0 | 89491 | FRR 0525 |  |  |  | 21962929 |  | Scaffold | 05/01/2015 | CCCX01 | 55318 | 410360 | 571 |  | UPPSALA UNIVERSITET | PRJEB6149 | SAMEA2500982 |  |  |  |

**Supplemental Table 2. Coordinates of complete MPA BGCs used and identified in this study.**

| Species | Contig | Start | End | Compl. contig | Start | End |
| --- | --- | --- | --- | --- | --- | --- |
| *Penicillium roqueforti* | NW_024067570.1 | 385000 | 440000 |  |  |  |
| *Penicillium psychrosexuale* | NW_026643191.1 | 130000 | 185000 |  |  |  |
| *Penicillium carneum* | HG816345.1 | HG817352.1 | HG818086.1 | /!\ all three as full contig ! |  |  |
| *Penicillium samsonianum* | NW_026643259.1 | 2025000 | 20780000 |  |  |  |
| *Penicillium E22* | JASJUN010000254.1 | 10000 | 44953 | JASJUN010000089.1 | 1 | 15000 |
| *Penicillium egyptiacum* | CAJVRC010000839.1 | 2005000 | 2060000 |  |  |  |
| *Penicillium brevicompactum* | NW_026623206.1 | 2085000 | 2140000 |  |  |  |
| *Penicillium bialowienzense* | JAKZFB010000004 | 2305000 | 2360000 |  |  |  |
| *Penicillium sp.MA6036* | QAGG01000163.1 | 1 | 40000 |  |  |  |
| *Penicillium sp.CF01* | RFFF02000036.1 | 1730000 | 1790000 |  |  |  |
| *Aspergillus pseudoglaucus* | JBDODC010000002.1 | 240000 | 295000 | JBDODC010000015.1 | 273000 | 284000 |
| *Aspergillus brunneus* | JAILXB010000055.1 | 1 | 40000 | JAILXB010000085.1 | 64000 | 75000 |
| *Paecilomyces niveus* | QEIL01000151.1 | 60000 | 110000 |  |  |  |
